# Effect of a person-centered care intervention on return and sustained reengagement after treatment interruptions from HIV care in Zambia: A post-hoc analysis of a stepped-wedged cluster randomized trial

**DOI:** 10.1371/journal.pmed.1005052

**Published:** 2026-09-01

**Authors:** Aaloke Mody, Kombatende Sikombe, Sandra Simbeza, Njekwa Mukamba, Laura K. Beres, Jake M. Pry, Anjali Sharma, Jacob Mutale, Alida Zulu Dube, Musunge Mulabe, Lloyd Mulenga, Suilanji Sivile, April Ratliff, Charles W. Goss, Charles B. Holmes, Carolyn Bolton Moore, Izukanji Sikazwe, Elvin H. Geng

**Affiliations:** 1 Department of Medicine, Washington University School of Medicine, St. Louis, Missouri, United States of America; 2 Centre for Infectious Disease Research in Zambia, Lusaka, Zambia; 3 Department of Public Health Environments and Society, Faculty of Public Health and Policy, London School of Hygiene and Tropical Medicine, London, United Kingdom; 4 Department of International Health, Johns Hopkins University Bloomberg School of Public Health, Baltimore, Maryland, United States of America; 5 Department of Public Health Sciences, School of Medicine, University of California, Davis, California, United States of America; 6 Zambian Ministry of Health, Lusaka, Zambia; 7 Center for Innovation in Global Health, Georgetown University, Washington, D.C., United States of America; 8 Division of Infectious Diseases, University of Alabama, Birmingham, Alabama, United States of America; 9 The Global Fund to Fight AIDS, Tuberculosis and Malaria, Geneva, Switzerland; Mass General Brigham, UNITED STATES OF AMERICA

## Abstract

**Background:**

Poor client-provider interactions lead to reinforcing cycles of treatment interruptions (TIs) from HIV care and fear of return, undermining sustained retention. We previously demonstrated a multi-component person-centered care intervention (PCC) targeting healthcare worker (HCW) behavior improved client experience and retention in HIV care. To understand mechanisms of improved retention, we evaluated the effect of the PCC intervention on return after TIs and sustained retention after return in a post-hoc analysis, hypothesizing that friendlier HCW attitudes enable successful reengagement into care.

**Methods and findings:**

We implemented the PCC intervention at 24 clinics in Zambia in a stepped-wedge, cluster randomized trial from Aug 2019 to Nov 2021. Clinics were allocated into 4 groups (8 clinics in Groups 1 and 4, and 4 each in Groups 2 and 3) and randomly assigned to crossover from control to intervention every 6 months. Under control, clinics provided routine HIV care without additional support. Afterwards, clinics received the PCC intervention targeting caring aspects of HCW behavior with 1) training and coaching on PCC practices, 2) measurement and feedback of client experience (via exit interviews), and 3) small facility-level incentives. We examined individuals living with HIV who became >30 days late to a scheduled visit (i.e., TI), categorizing intervention exposure based on the last visit prior to TI. We used electronic health records to assess the effect of the PCC intervention on 1) return to HIV care after a TI, and 2) repeat TIs among those who returned. We used multistate analytic methods to estimate crude incidence of return and repeat TIs and prevalence of being in care at 1-year. We used shared frailty Cox proportional hazards and mixed-effects Poisson models to obtain formal comparisons that accounted for the stepped-wedge design. Individuals were censored at intervention crossover or database closure. During the study period, 128,910 clients (*n* = 69,671 control, *n* = 59,239 intervention) became >30 days late (64.3% female, median age 38y [IQR 31,45], median years in care 2.5y [IQR 0.6,6.7]). The PCC intervention increased incidence of return after TI at 12-months (72.3% intervention versus 67.7% control, crude risk difference [RD] +4.6% [CI 4.1,5.2], adjusted hazard ratio [aHR] 1.16 [CI 1.12,1.20]; *p* < 0.001). Among returners, the intervention decreased incidence of repeat TIs (44.3% versus 55.6%, RD −11.4% [CI −12.2,−10.6], aHR 0.50 [CI 0.45,0.55]; *p* < 0.001) and increased the proportion in care (with or without repeat TIs) (82.7% versus 73.4%, RD +9.3% [CI 8.6,10.0], adjusted risk ratio [aRR] 1.14 [CI 1.05,1.25]; *p* = 0.002) at 12-months after return. Overall, the PCC intervention increased the proportion in care 12-months after a TI from 51.5% to 60.1% (RD +8.7% [CI 8.0,9.3], aRR 1.19 [CI 1.05,1.35]; *p* = 0.008). Effect sizes were similar in direction and magnitude in multiple sensitivity analyses with varying precision. Limitations included post-hoc analysis and those associated with stepped-wedge design.

**Conclusions:**

The PCC intervention likely improved rates of return to care after TIs, sustained reengagement after return, and overall retention among individuals with TIs. Strategies targeting caring aspects of HCW behavior and client experience may help lower barriers to and address persistent challenges with reengagement in care.

## Author summary

**Why was this study done?:**

- Treatment interruptions (TIs) remain one of the most enduring challenges to sustained viral suppression in HIV care, and most people who interrupt will go on to have repeat lapses.
- Poor, disrespectful client-provider interactions are a key but under-addressed barrier that can foster fear, guilt, and reluctance to return, yet there are still few evidence-based strategies to improve return to care and durable reengagement in this critical population.
- We previously showed that a person-centered care (PCC) intervention targeting healthcare worker behavior improved client experience and overall retention, and in this manuscript we sought to understand the mechanism of that improvement—whether it worked by helping people return to care, helping them stay in care after returning, or both.

**What did the researchers do and find?:**

- We implemented the PCC intervention—comprising healthcare worker training and coaching, systematic measurement and feedback of client experience, and a small facility-level incentive—at 24 public HIV facilities in Lusaka, Zambia, in a stepped-wedge, cluster randomized trial, and used electronic health records to study reengagement among 128,910 individuals who experienced a treatment interruption.
- The proportion of people who returned to care 1 year after a treatment interruption was greater in the PCC intervention arm (72.3% intervention versus 67.7% control) and, among those who returned, there were fewer repeat TIs (44.3% versus 55.6%) and a higher proportion remaining in care (82.7% versus 73.4%) in the intervention arm.
- Overall, the proportion of individuals in care 1 year after a treatment interruption was 60.1% in the PCC intervention arm and 51.5% in control.
- These results were robust in multiple sensitivity analyses that varied key design and analytic decisions, although time-to-event estimates restricted to Groups 1 and 4 were less precise and did not meet conventional significance thresholds.

**What do these findings mean?:**

- Targeting the caring aspects of healthcare worker behavior and improving client experience can lay the foundation for easier reengagement, facilitating people both returning to care and staying in care after a treatment interruption.
- These improvements were achieved among individuals with TIs—a population that has face persistent challenges with improving outcomes and represents a remaining frontier in achieving population-level viral suppression.
- Investing in client-centered care environments may be a feasible, sustainable, and effective public health strategy, with relevance not only for HIV care but for longitudinal primary and chronic disease care across low- and middle-income settings.
- Limitations included the post-hoc nature of the analysis, unequal observations times due to stepped-wedge design, and possibility of selection bias as the PCC intervention may have prevented TIs to begin with.

## Abstract 3

In a post-hoc analysis of an implementation trial, Aaloke Mody and colleagues show that a person-centered care strategy targeted to healthcare workers is linked to improve return to and subsequent retention in HIV care of persons who experience treatment interruptions.

## Introduction

Despite scale-up of universal testing and treatment for HIV and widespread access to ART, a substantial proportion of individuals diagnosed with HIV are not virally suppressed [1–7]. Lapses from HIV care remain one of the most enduring challenges to viral suppression among people living with HIV (PLWH), with 20%–30% of PLWH experiencing a treatment interruption from care within 2 years of initiation [8–12]. Retention in care is dynamic with PLWH frequently transitioning in and out of care over time [13–19]: even though a majority of individuals who experience an interruption eventually return to care, initial treatment interruptions (TIs) can often be prolonged and then 30%–50% of those who do return will also go on to have a repeat lapse [9,16,17,20,21] from care. This is due to the persistent and varied challenges, such as competing work and life obligations, travel/mobility, clinic-based barriers, or psychosocial barriers [22–28], that make sustained engagement elusive. PLWH experiencing TIs have higher rates of viremia (~70%) [9,13–15,18,19,24,29–32] and likely represent the largest pool of population-level viremia among those who have been diagnosed with HIV. Furthermore, the majority of individuals now hospitalized with advanced HIV and also those presenting as new diagnoses have previously been on ART and experienced a treatment interruption [16,33]. Despite being a well-established challenge, there are still few evidence-based strategies to promote durable reengagement after treatment lapses in this critical population segment [24,34–37].

Poor client-provider interactions represent a key but under-addressed barrier to sustained engagement in HIV care. Disrespectful or dismissive interactions can create reinforcing cycles of disengagement, with clients frequently describing feeling “uncared for” as a major reason for dropping out of care [25,38–42]. Theories on disengagement suggest that poor interpersonal experiences can both instigate a treatment interruption, but also prolong or exacerbate interruptions that may be triggered by other circumstances such as competing work, school, or family obligations [38]. Regardless of the initial trigger, negative provider interactions prior to the interruption can foster shame or guilt and instill fear, anxiety, and reluctance to return to care, thus preventing or delaying return [38,43]. If clients who do return encounter similarly unwelcoming environments, this could further make them feel undervalued and undermine their sense of connection at the facility, further reinforcing ongoing cycles of care lapses [38,43]. Healthcare workers themselves recognize and understand these principles, but also report the tension in maintaining their professional identities and responsibilities in the face of a less supportive working environment [44]. Thus, targeting provider behaviors to enable a more welcoming and client-centered care experience has the promise to strengthen reengagement by both reducing impediments to reentry and strengthening ongoing connection and engagement, and there is emerging evidence for such approaches [43].

To further address this gap, we developed a multi-component person-centered care (PCC) intervention targeting providers behaviors through a human-centered design process [44,45]. The PCC strategy included provider training and ongoing coaching on person-centered principles, systematic measurement and feedback of client experience, and a small facility-level incentive for performance [44,45]. We previously reported that the PCC intervention led to significant improvements in client experience, decreased missed visits, and improved overall retention in care [45]. In this manuscript, we evaluate the effect of the PCC strategy on reengagement among individuals who experienced TIs to better understand the different mechanisms by which retention improved (e.g., improved reengagement versus preventing initial TIs) in a post-hoc analysis. We use multistate analytic approaches to assess transitions into and out of care over time and identify the pathways that influence reengagement outcomes [46–48]. We hypothesized that, by improving client-provider interactions, the PCC strategy would enhance both the rates of return after care lapse as well as sustained engagement in care among those who return (Fig A in S1 Appendix).

## Methods

### Study design

We carried out a type II hybrid implementation effectiveness trial of the PCC strategy using a stepped-wedge, cluster randomized design at 24 public sector facilities in Lusaka Province, Zambia between 12th August 2019 and 30th November 2021. Clinics were operated by the Zambian Ministry of Health (MOH) and supported by the Centre for Infectious Disease Research in Zambia (CIDRZ), a Zambian nongovernmental organization (NGO). The study objective was to assess how a multi-component PCC strategy comprising (1) HCW training and coaching (i.e., practice facilitation), (2) systematic measurement of client experience and feedback to HCWs, and (3) small performance-based facility-level incentives would change HCW behavior and the client experience, and then ultimately how these changes impacted client outcomes under real world service delivery conditions in Zambia [45]. The primary analysis showed improvements in client experience (with consistent results when restricted to returners), decreased missed visits, and increased retention in care at 15-months, but did not demonstrate effects on viral suppression in a small nested subcohort [45]. In this manuscript, we examine the effects of the PCC strategy on reengagement in care among those who experienced TIs during the study to better understand the mechanisms underlying the demonstrated impacts on retention in a post-hoc analysis (Fig A in S1 Appendix).

The PCC trial received approval (including a waiver of consent to utilize electronic health records [EHR] records) from the Institutional Review Boards (IRB) of the University of Zambia (008-03-19) and the University of Alabama at Birmingham (UAB) (IRB-300003282) and the Zambian MOH, with other US institutions using a formal IRB reliance agreement with UAB. The trial was registered with the Pan African Trial Registry (PACTR202101847907585). This manuscript represents a non-prespecified secondary analysis to better understand effects seen in primary analysis. This study is reported as per CONSORT guideline for stepped-wedge cluster randomized trials (CONSORT S1 Checklist).

### Study participants

For this analysis, we used EHR data to assess reengagement among clients who 1) had made at least one visit to one of the 24 study clinics during the study period and 2) then subsequently became >30 days late to a scheduled visit (i.e., treatment interruption). Thus, all included individuals had exposure to either intervention or control conditions during the study prior to having a treatment interruption. We defined exposure to intervention or control conditions using clinic intervention status at the last made visit immediately prior to the TIs as the study was conducted at the facility-level and exposures to study procedures only occurred during visits. We only included clients at the time of their first treatment interruption during the study (i.e., we did not include individuals again in the intervention arm if they previously contributed observation time for a treatment interruption during control periods).

### Randomization

A statistician not otherwise involved in the study randomly assigned clinics into four groups stratified by clinic size and viral load monitoring coverage. We allocated eight clinics to Groups 1 and 4 and four each to Groups 2 and 3 to place more clinics (an important driver of statistical power) in groups with longer continuous periods of exposure in either the treatment or control conditions to better assess longitudinal outcomes. We introduced the intervention sequentially to the four groups every 6-months but due to study interruption caused by the COVID-19 pandemic, period 2 was extended from 6 to 9 months. Investigators and providers were not masked to intervention status, but clients were masked (Fig B in S1 Appendix).

### Intervention procedures

The PCC strategy was a multi-component approach targeting HCW behavior based on formative work highlighting the importance of friendliness, respect, dignity, adequate communication, and involving clients in decision-making. We conceptualized these ideas using a mechanistic theory of change [49–51] based on Green’s PRECEDE-PROCEED approach (which advocates for interventions that predispose, enable and reinforce behavior change) and Michie’s Capability—Opportunity—Motivation segmentation of mechanisms of behavior change [52,53] (Fig C in S1 Appendix), and developed the actual intervention protocol through a week-long human-centered design workshop in 2018 with 20 HCWs [44]. The intervention comprised of three components. First, we delivered a 2-day interactive, off-site training led by nurses to build knowledge and skills for PCC, targeting all clinic staff at the facility across departments, although with specific attention to reaching everyone in the ART department. Content focused on communication, stress management, teamwork, and person-centered principles (e.g., empathy, shared decision-making). Training was followed by weekly to monthly facilitation meetings to help translate concepts into practical steps in day-to-day care. Second, we systematically measured client experience using 12-item exit surveys adapted from validated instrument [54,55]. These results were routinely fed back to staff during quarterly meetings where other quality metrics (e.g., viral load suppression) were also reviewed. Third, we provided biannual clinic-level incentives up to $75 based on performance on client experience metrics. Additional details on implementation of study procedures are described in further detail in the Supplemental Appendix and reports of the parent PCC trial findings [45].

### Measurements

We used data from the national electronic health record and laboratory systems for routine HIV care in Zambia to obtain sociodemographic (e.g., age, sex, and clinic site), visit history (e.g., HIV clinic enrollment date, ART initiation date, follow-up visits), clinical (e.g., viral loads, ART regimen, enrollment CD4 count, WHO Stage), and facility-level (e.g., size) measurements. At the time of the study, the electronic health record was populated by providers completing standardized electronic clinical forms: laboratory results were printed at the main laboratory, sent to the clinic, and inputted in the electronic health record. Trained MOH data clerks entered all information into the electronic database on an ongoing basis, and CIDRZ performed routine data quality audits and updates at least quarterly to ensure relatively high-quality data through its engagement in programmatic technical assistance. Providers were masked to outcome ascertainment, and procedures were identical for all outcomes across all sites.

### Outcomes

**Fig 1:**
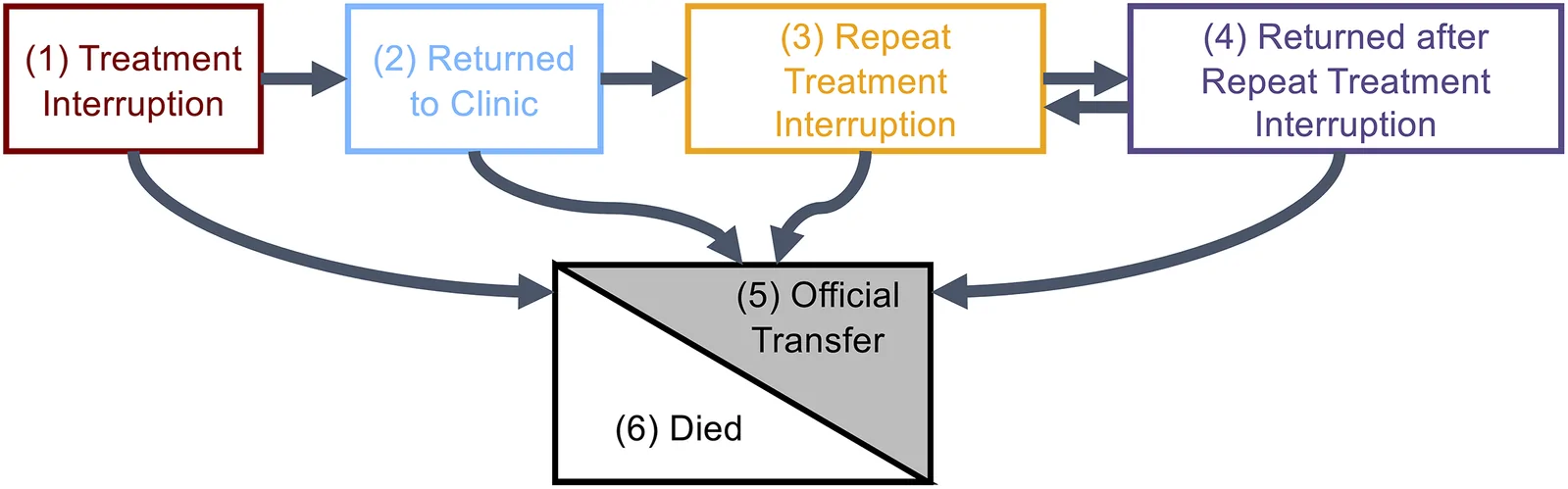
State transition flowchart for multi-state analysis. This figure depicts all possible transitions between care states over time. At each time point after cohort entry (i.e., time of initial treatment interruption, clients were categorized into 1 of 6 mutually exclusive and exhaustive states: (1) Treatment Interruption, (2) Returned to Clinic without Repeat Treatment Interruption, (3) Repeat Treatment Interruption, (4) Return after Repeat Treatment Interruption, (5) Official Transfer, and (6) Died.

We used multistate framework to characterize reengagement dynamics and track individuals’ transitions in and out of care over time. We first used EHR data to categorize individuals care status into 6 mutually exclusive and exhaustive states at each time point after their initial treatment interruption: (1) initial treatment interruption (i.e., >30 days late for scheduled visit) (2) return to care, (3) repeat treatment interruption, (4) return after a repeat treatment interruption, (5) documented transfer, and (6) death. These care states were defined specifically to incorporate prior history (e.g., initial versus repeat treatment interruption) (Fig 1) to address limitations stemming from the Markov assumption used in multistate analyses.

Using this framework, we defined four main outcomes of interest to evaluate return after a treatment interruption and also sustained retention after return. Among everyone with a treatment interruption, we examined (1) time to any return visit (i.e., time to transition to state 2), and (2) the proportion who were in care at 1-year after the initial treatment interruption (i.e., defined as being in either state 2 or state 4 at the 365 day time point). Among individuals who did return after the initial treatment interruption (i.e., entered state 2), we also examined (3) time to a repeat treatment interruption (i.e., transitioned from state 2 to state 3), and (4) the proportion in care 1-year after returning (i.e., in either state 2 or state 4 at the 365 day time point after return).

### Statistical analysis

We used nonparametric, time-to-event multistate analytic methods based on the Aalen-Johansen method to first describe crude reengagement outcomes that accounted for individuals’ transitioning in and out of care over time [46–48]. Time zero was either the date of initial treatment interruption (time to return, proportion in care 1-year after TI), or, for analyses specifically examining outcomes after return (time to repeat TI, proportion in care 1-year after return), time zero was reset to the date of return. Clients were followed until entering an absorbable state (i.e., official transfer or death) or being censored either at the end of the observation period on 10 Jan 2022 or when crossing over from control to intervention conditions (only applicable to individuals in control). The timing of intervention crossover was defined at an individuals’ first clinic visit after the clinic transitioned from control to intervention conditions (i.e., their first exposure to intervention conditions). To define outcomes for all visits, we imputed any appointment dates that were not captured in the EHR by using the mean appointment interval for the clinic that day (and mean over period to address any residual missingness). The assumptions underlying this approach are based on the observation that facilities often distribute the same duration of prepackaged drugs to all established clients each day. For multistate analyses, individuals could transition between nonabsorbable states an unlimited number of times based on the Markov assumption until they transitioned into an absorbable state or were censored [46–48]. We estimated the crude absolute risk for each outcome and crude risk differences (RD) between intervention and control conditions over time, and used bootstrapping (5,000 iterations) to obtain 95% confidence intervals (CIs).

Multistate analyses provide nuanced descriptive estimates, but, importantly, the crude comparisons do not fully account for the stepped-wedge study design. To obtain formal tests of the effect of the PCC strategy, we used standard regression models that could appropriately account for the study design. For the two time-to-event outcomes of return after a treatment interruption and repeat treatment interruption after return, we used cause-specific Cox proportional hazards regression among the full population with censoring as described above. For analyses of binary outcomes of being in care 1-year after the initial treatment interruption and, among returners, in care 1-year after return, we needed to restrict participants to only include individuals that had sufficient uninterrupted observation time in either control or intervention conditions for the 1-year outcome to unfold without clinic crossover or censoring, and also fix crossover at time of clinic transition (rather than an individual’s first visit after transition). These modifications were necessary to avoid bias in outcome ascertainment of binary outcomes and create an epidemiologically robust albeit restricted sample for these analyses. We then used mixed-effects Poisson models with robust variances for binary outcomes [56]. For all regression models, we included facility as either a shared frailty (cause-specific Cox models) or random effect (Poisson models) and study period as fixed-effects according to the Hussey-Hughes method for analyzing stepped-wedge trials [57], and also adjusted for sex, age, time since ART initiation, scheduled appointment interval at last visit, and facility type to account for any imbalances between intervention and control arms and limit any bias from selection (covariates had no missingness except for <0.1% missingness in age) (Fig D in S1 Appendix). We performed overall analyses as well as analyses stratified by sex and age, time on ART, scheduled appointment interval at time of initial treatment interruption, enrollment WHO stage, marital status, education status, and facility type (hospital-based versus nonhospital-based clinic) to examine consistency of effects. We evaluated proportional hazards assumption using scaled Schoenfeld residual plots.

We also performed several sensitivity analyses to evaluate the robustness of our results. First, we restricted analyses to Groups 1 and 4 in a nested parallel-cluster randomized design to evaluate for potential biases from the stepped-wedge design. These groups had the longest contemporaneous and continuous periods of exposure to either intervention or control conditions without crossover. All individuals in both Groups 1 and 4 were censored at the time of their first made visit after Period 3 had ended (i.e., when Group 4 transitioned to intervention status). Second, we performed analyses excluding any individuals who had any imputed appointment intervals due to missing appointment dates and may have had inclusion TIs or subsequent reengagement based on imputed values. Lastly, we performed a sensitivity analysis defining a treatment interruption as being greater than either 60 or 90 days late.

All analyses were conducted with R 4.3.1 software (R Foundation for Statistical Computing, Vienna, Austria) using the *mstate* package [46,47] and Stata MP 17.0 (Stata Corp LLC, College Station, Texas). Sample size considerations for this post-hoc analysis given known parameters are discussed in Supplemental Appendix (Table A in S1 Appendix).

## Results

**Fig 2:**
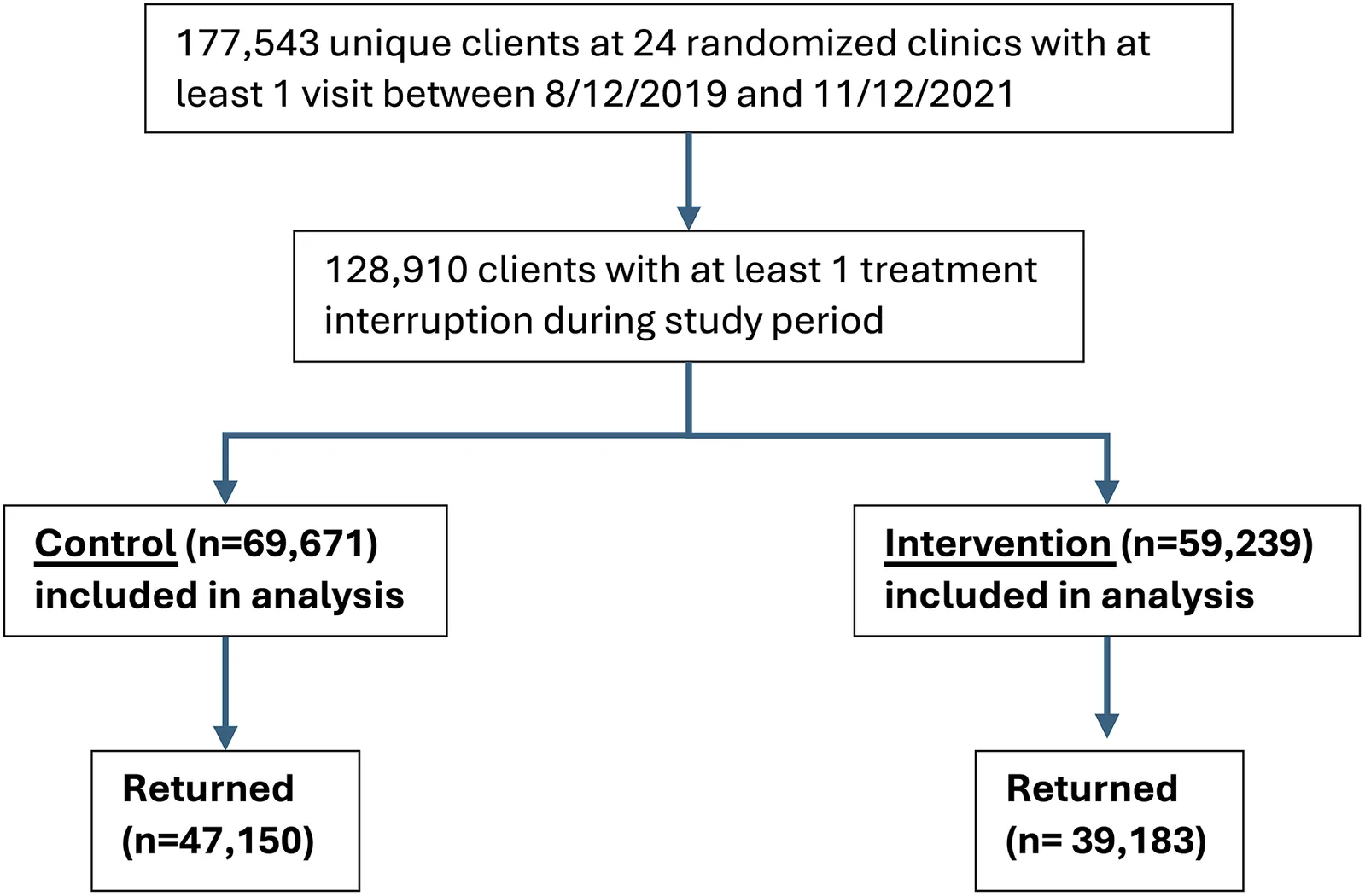
Flow diagram of inclusion criteria for analysis of outcomes after treatment interruption. 177,543 unique clients made at least one visit during the study period at one of the 24 clinics and were at some point exposed to either intervention or control periods (or both), and 128,910 clients experienced at least 1 treatment interruption (*n* = 69,671 in Control Arm and *n* = 59,239 in Intervention Arm). Among participants with a treatment interruption, 86,333 eventually returned to care (*n* = 47,150 in Control Arm and *n* = 39,183 in Intervention Arm) and were included in analyses examining outcomes after return.

Among 177,543 unique clients who made a visit to one of the 24 study clinics between August 2019 and November 2021, 128,910 experienced at least one treatment interruption (*n* = 69,671 control, *n* = 59,239 intervention) (Fig 2, Table B in S1 Appendix). Overall, 82,890 (64.3%) were female, median age at treatment interruption was 38 years (IQR 31,45), and 30,800 (23.9%) experienced a treatment interruption within 6 months of starting ART. Client characteristics were generally balanced across intervention and control arms (except for small imbalances in years on ART and scheduled appointment interval) and were representative of typical client mix in Lusaka province (Table 1). Missing appointment dates reduced over time, and were most frequent during Period 2 during the COVID-19 pandemic (Table C in S1 Appendix).

**Table 1:** Participant characteristics.

		After treatment interruption			Among returners		
**Control (*n* = 69,671)**	**Intervention (*n* = 59,239)**	**Standardized Difference**	**Control (*n* = 47,150)**	**Intervention (*n* = 39,183)**	**Standardized difference**
Sex, *n* (%)	Female	44,994 (64.6%)	37,896 (64.0%)	0.01	30,427 (64.5%)	24,983 (63.8%)	0.02
	Male	24,677 (35.4%)	21,343 (36.0%)		16,723 (35.5%)	14,200 (36.2%)	
Median age at treatment interruption, years (IQR)		38 (31, 45) (*n* = 69,664)	38 (30, 45) (*n* = 59,230)	0.02	39 (32, 46) (*n* = 47,143)	39 (32, 46) (*n* = 39,174)	0.01
Age at treatment interruption, *n* (%)	<25 y	5,935 (8.5%)	5,386 (9.1%)	0.02	3,223 (6.8%)	2,723 (6.9%)	0.01
	25–44 y	46,025 (66.1%)	39,080 (66.0%)		30,605 (64.9%)	25,430 (64.9%)	
	>45 y	17,704 (25.4%)	14,764 (24.9%)		13,315 (28.2%)	11,021 (28.1%)	
	Missing	7 (<1%)	9 (<1%)		7 (<1%)	9 (<1%)	
Years on ART prior to treatment interruption, *n* (%)	<6 m	15,210 (21.8%)	15,590 (26.3%)	0.11	6,577 (13.9%)	5,809 (14.8%)	0.04
	6 m to 2y	14,554 (20.9%)	12,012 (20.3%)		10,022 (21.3%)	8,523 (21.8%)	
	2–5 y	15,691 (22.5%)	12,898 (21.8%)		11,526 (24.4%)	9,775 (24.9%)	
	>5 y	24,216 (34.8%)	18,739 (31.6%)		19,025 (40.3%)	15,076 (38.5%)	
Scheduled appointment interval*, *n* (%)	14 days	4,203 (6.0%)	3,300 (5.6%)	0.14	914 (1.9%)	609 (1.6%)	0.09
	30 days	7,642 (11.0%)	7,753 (13.1%)		3,710 (7.9%)	2,383 (6.1%)	
	60 days	2,166 (3.1%)	1,745 (2.9%)		1,327 (2.8%)	953 (2.4%)	
	90 days	38,382 (55.1%)	34,712 (58.6%)		21,895 (46.4%)	18,797 (48.0%)	
	120 days	4,976 (7.1%)	3,905 (6.6%)		2,749 (5.8%)	2,776 (7.1%)	
	180 days	12,302 (17.7%)	7,824 (13.2%)		16,555 (35.1%)	13,665 (34.9%)	
WHO stage at enrollment, *n* (%)	Stage 1	37,293 (53.5%)	31,376 (53.0%)	0.04	23,621 (50.1%)	20,209 (51.6%)	0.06
	Stage 2	8,247 (11.8%)	6,592 (11.1%)		6,221 (13.2%)	4,845 (12.4%)	
	Stage 3	10,014 (14.4%)	7,733 (13.1%)		8,097 (17.2%)	5,934 (15.1%)	
	Stage 4	748 (1.1%)	677 (1.1%)		609 (1.3%)	539 (1.4%)	
	Missing	13,369 (19.2%)	12,861 (21.7%)		8,602 (18.2%)	7,656 (19.5%)	
Marital status, *n* (%)	Single	8,682 (12.5%)	6,976 (11.8%)	0.02	5,509 (11.7%)	4,087 (10.4%)	0.03
	Married	37,424 (53.7%)	31,534 (53.2%)		26,092 (55.3%)	21,425 (54.7%)	
	Divorced	7,928 (11.4%)	6,538 (11.0%)		5,178 (11.0%)	4,085 (10.4%)	
	Widowed	5,200 (7.5%)	4,238 (7.2%)		3,764 (8.0%)	3,032 (7.7%)	
	Missing	10,437 (15.0%)	9,953 (16.8%)		6,607 (14.0%)	6,554 (16.7%)	
Education, *n* (%)	None	3,364 (4.8%)	2,702 (4.6%)	0.02	2,463 (5.2%)	1854 (4.7%)	0.03
	Primary	17,252 (24.8%)	14,510 (24.5%)		12,027 (25.5%)	9,953 (25.4%)	
	Secondary	34,260 (49.2%)	29,896 (50.5%)		23,093 (49.0%)	19,366 (49.4%)	
	University	3,756 (5.4%)	3,323 (5.6%)		2,614 (5.5%)	2,229 (5.7%)	
	Missing	11,039 (15.8%)	8,808 (14.9%)		6,953 (14.7%)	5,781 (14.8%)	
Facility type, *n* (%)	Not hospital-based	38,398 (55.1%)	30,267 (51.1%)	0.08	25,127 (53.3%)	21,099 (53.8%)	0.01
	Hospital-based	31,273 (44.9%)	28,972 (48.9%)		22,023 (46.7%)	18,084 (46.2%)	
Group, *n* (%)	1	0 (0.0%)	42,084 (71.0%)	–	0 (0.0%)	30,142 (76.9%)	–
	2	7,934 (11.4%)	10,326 (17.4%)		6,315 (13.4%)	7,294 (18.6%)	
	3	20,721 (29.7%)	4,523 (7.6%)		14,762 (31.3%)	1,570 (4.0%)	
	4	41,016 (58.9%)	2,306 (3.9%)		26,073 (55.3%)	177 (0.5%)	
Period, *n* (%)	1	36,926 (53.0%)	19,248 (32.5%)	–	27,535 (58.4%)	15,047 (38.4%)	–
	2	24,950 (35.8%)	22,599 (38.1%)		17,955 (38.1%)	18,243 (46.6%)	
	3	7,795 (11.2%)	9,697 (16.4%)		1,660 (3.5%)	5,053 (12.9%)	
	4	0 (0.0%)	7,695 (13.0%)		0 (0.0%)	840 (2.1%)	

* Scheduled Appointment Interval either at the last visit prior to treatment interruption (After Treatment Interruption) or at the return visit (Among Returners). ART, antiretroviral therapy; m, month; y, year. Groups represent the clusters of clinics randomly assigned for intervention rollout at different time periods; Period represents the study period for the stepped-wedge design. Standardized differences not presented for Group and Period as these are imbalanced by design.

At the start of intervention periods, we provided training to 2,567 HCWs and ancillary staff (e.g., security guards, cleaners) across all clinics, which provided training coverage for ~90% of HCWs working in ART Departments and 65% of staff across all departments (Table D in S1 Appendix). Clinics received a median of 3 coaching visits per month (IQR 2–4) (1,234 total coaching visits) during intervention periods (Fig E in S1 Appendix), but frequencies did vary over time (Table E in S1 Appendix). Additionally, all facilities had meetings every 3 months to review client experience measurements and prioritize indicators, and reputational incentives were delivered on a biannual basis.

**Fig 3:**
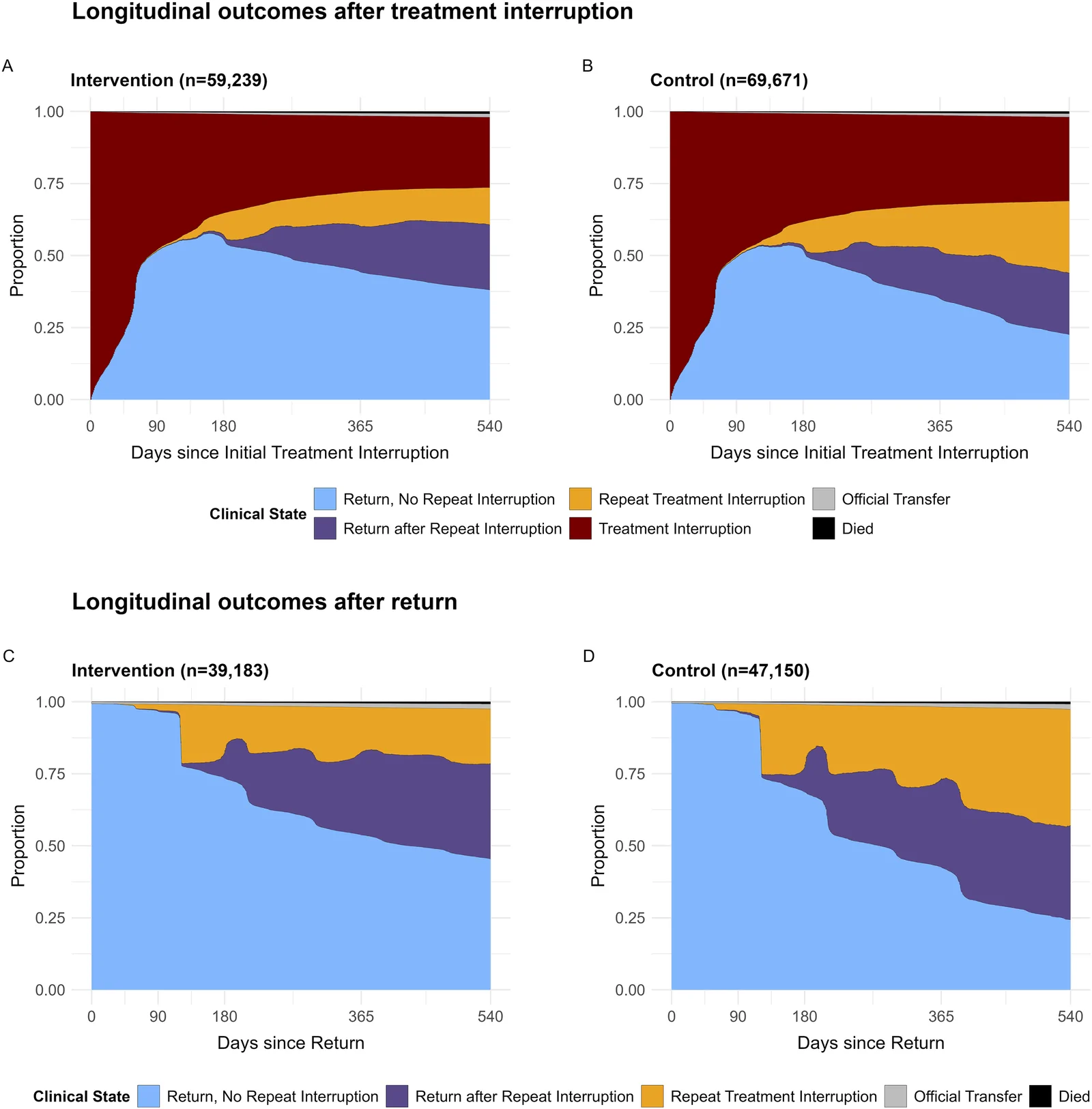
Longitudinal outcomes by intervention status after initial treatment interruption and after return. This figure shows the crude proportion of clients estimated to be in each care state at any given time after an individual experiences either their initial treatment interruption (Panels **A** and **B**) or after returning to care (Panels **C** and **D)**, stratified by intervention status. See Tables F and G in S1 Appendix.

**Fig 4:**
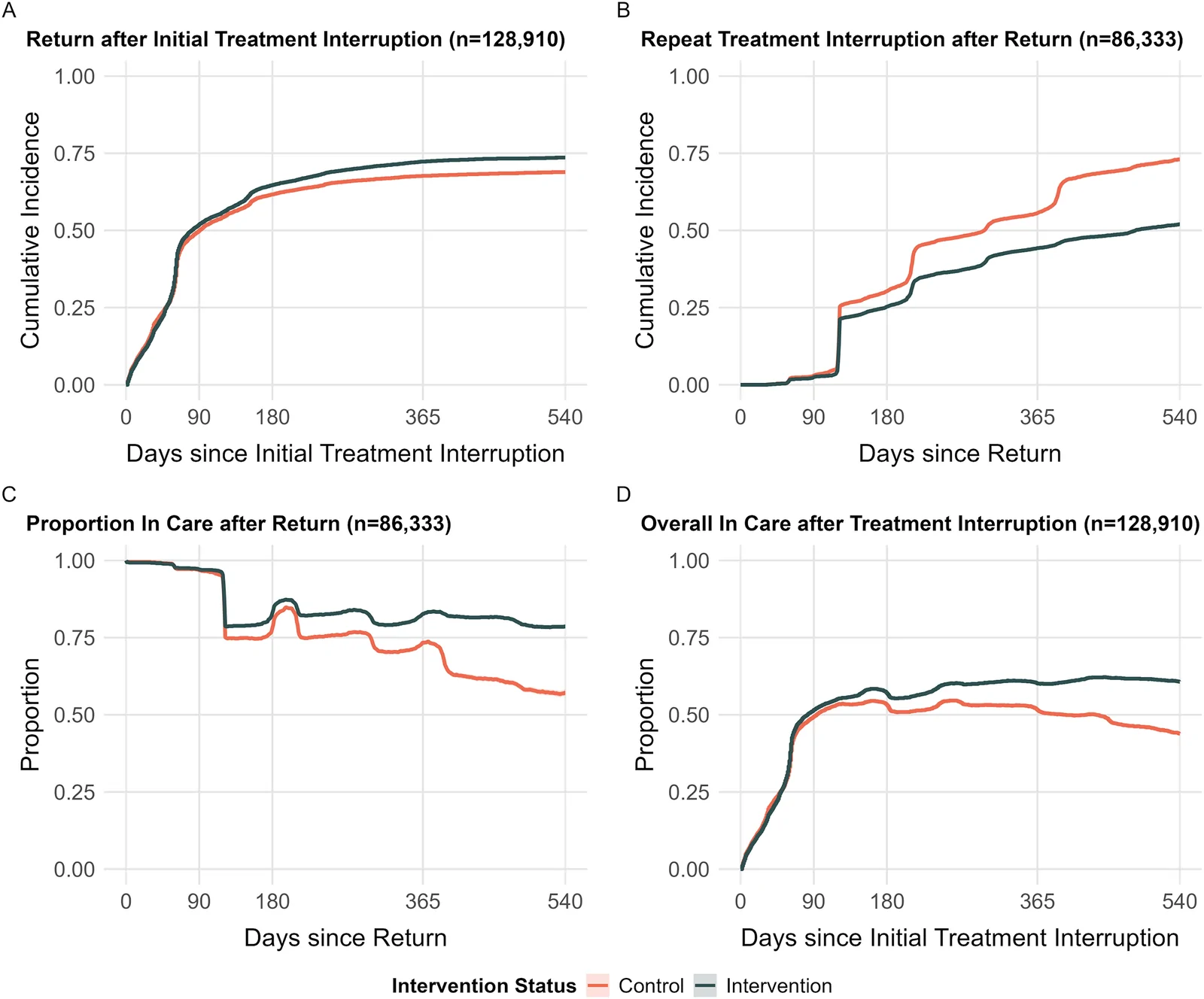
Effect of PCC intervention on return to care and retention after return after treatment interruption. This figure shows the effect of the PCC intervention over time with 95% confidence intervals on our four main outcomes of interest: **(A)** Return to Care after treatment interruption, **(B)** Repeat Treatment Interruption after Return, **(C)** Proportion in Care after Return, and **(D)** Overall Proportion in Care after Initial Treatment Interruption. All outcomes estimated as composites of individual care states from multistate analyses using bootstrapping to estimate 95% confidence intervals.

Among all individuals who experienced a treatment interruption (*n* = 128,910), a crude cumulative incidence of a return visit by 6-months after the interruption was 61.7% (95% CI 61.3, 62.0%) in control arms and 64.7% (95% CI 64.3, 65.1%) in the PCC intervention (crude RD 3.0% [95% CI 2.5, 3.6%]). This difference persisted at 12-months, with 67.7% (95% CI 67.3, 68.0%) in control and 72.3% (95% CI 71.9, 72.7%) in the intervention arms having returned (RD 4.6% [95% CI 4.1, 5.2%]) (Figs 3 and 4, Tables F and G in S1 Appendix).

Among individuals who did return to clinic (*n* = 86,333), the crude cumulative incidence of repeat TIs went from 30.3% (95% CI 29.8, 30.8%) in control to 25.3% (95% CI 24.9, 25.8%) in intervention at 6-months after return (RD −5.0% [95% CI −5.6, −4.3%]), with the difference increasing to a 11.4% reduction in repeat TIs (95% CI −12.2, −10.6%) by 12 months (55.6% [95% CI 55.0, 56.2%] in control versus 44.3% [95% CI 43.7, 44.8%] in intervention). Additionally, the proportion of individuals who were in care after return (i.e., not in a treatment interruption) increased by 4.9% (95% CI 4.4, 5.5%) at 6-months after return (78.2% [95% CI 77.7, 78.6%] in control versus 83.1% [95% CI 82.7, 83.5%] in intervention) and by 9.3% (95% CI 8.6, 10.0%) at 12-months (73.4% [95% CI 72.8, 74.0%] in control versus 82.7% [95% CI 82.2, 83.1%] in intervention) (Figs 3 and 4, Tables F and G in S1 Appendix).

Lastly, among all individuals who experienced a treatment interruption (*n* = 128,910), the crude overall proportion in care at 6-months after the initial interruption also increased from 53.1% (95% CI 52.7, 53.5%) in control versus 56.9% (95% CI 56.5, 57.3%) in intervention (RD 3.8% [95% CI 3.2, 4.4%]). This increased further from 51.5% (95% CI 51.0, 51.9%) to 60.1% (95% CI 59.7, 60.6%) at 12-months (RD 8.7% [95% CI 8.0, 9.3%]) and from 43.9% (95% CI 43.3, 44.6%) to 60.7% (95% CI 60.2, 61.2%) at 18-months (RD 16.7% [95% CI 15.9, 17.6%]) (Figs 3 and 4, Tables F and G in S1 Appendix).

In adjusted analyses that appropriately accounted for stepped-wedge cluster randomized design, exposure to the PCC intervention led to increased rates of return (adjusted cause-specific hazard ratio [aHR] 1.16 [95% CI 1.12,1.20]; *p* < 0.001), and also an increased likelihood of being in care at 12, months after the treatment interruption (aRR 1.19 [95% CI 1.05, 1.35]; *p* = 0.008) (Tables 2 and H, Figs F and G in S1 Appendix). Similarly, among those who returned, the PCC intervention led to decreased rates of repeat TIs (aHR 0.50 [95% CI 0.45, 0.55]; *p* < 0.001) and also an increased likelihood of being in care 12-month after return (aRR 1.14 [95% CI 1.05, 1.25]; *p* = 0.002) (Tables 2 and H, Figs F and G in S1 Appendix). Characteristics were generally well balanced between individuals included in and restricted from analyses of binary outcomes (except for some imbalance in scheduled appointment dates) (Table I in S1 Appendix).

**Table 2:** Effect of PCC intervention on return to care and retention from adjusted regression models.

After initial treatment interruption			
	**Adjusted cause-specific hazard ratio** **(95% CI)**	***p*-value**	**ICC**
Time to return (*n* = 128,894)	1.16 (1.12, 1.20)	<0.001	0.033
	**Adjusted risk ratio** **(95% CI)**	***p*-value**	**ICC**
In care at 365 days (*n* = 51,583)	1.19 (1.05, 1.35)	0.008	0.031
After return			
	**Adjusted cause-specific hazard ratio** **(95% CI)**	***p*-value**	**ICC**
Time to repeat treatment interruption (*n* = 86,317)	0.50 (0.45, 0.55)	<0.001	0.058
	**Adjusted risk ratio** **(95% CI)**	***p*-value**	**ICC**
In care at 365 days after return (*n* = 30,085)	1.14 (1.05, 1.25)	0.002	0.036

ICC, Intraclass Correlation. Analyses adjusted for study facility as shared frailty (Cox Model) or random effect (mixed-effects Poisson model with robust variances) and study period as fixed-effects. Analyses also included adjustment for sex and age (including sex*age interaction), time since ART initiation, scheduled appointment interval at either last visit or time of return, and facility type. Individuals with missing age were excluded from these analyses.

Effect sizes for both crude and adjusted estimates were similar in direction and magnitude in multiple sensitivity analyses restricted to Group 1 and 4 clinics in a nested parallel-cluster randomized specification, excluding those with imputed appointment intervals, and alternative definition of treatment interruption (although time-to-event analyses for Group 1 and 4 did not meet conventional thresholds for statistical significance) (Tables J and K in S1 Appendix). The effects of PCC on return to care, repeat TIs, and proportions in care were also generally consistent in analyses that were stratified across age, sex, care history, and study period (Fig H and Table L in S1 Appendix).

## Discussion

This study demonstrates that a multi-component PCC intervention targeting friendlier and more welcoming provider behaviors likely improved outcomes among individuals with TIs, a population that represents a remaining frontier in achieving population-level viral suppression. After a treatment interruption, the PCC intervention led to a 4.6% point increase in return to care 1 year later (67.7% versus 72.3%), and, among those who did return, there was an 11.4 percentage point decrease in repeat TIs (55.6% versus 44.3%) and a 9.3 percentage point increase in the proportion in care after return (73.4% versus 82.7%). By improving both rates of return after TIs and retention in care after return, the PCC intervention increased the overall proportion of individuals who were in care 1 year following TIs by 8.7 percentage points (51.5% versus 60.1%). Effect sizes were consistent in crude and adjusted analyses accounting for study design as well as sensitivity analyses that examined effects with alternative cohort specifications. Considered in the context of previously reported findings on improved client experience and overall retention [45], this study advances the robust, growing evidence that targeting provider behaviors and their interpersonal interactions with clients is a vital public health strategy, particularly among populations facing greatest challenges.

The underlying hypothesis for this analysis was informed by existing theoretical frameworks on disengagement from HIV care [25,38–41], and its results now provide robust empirical evidence for the effect of the PCC intervention among those with TIs that aligns with and extends these theories [51]. Norma Ware’s theory of HIV disengagement suggests that negative healthcare experiences foster a reluctance to return to care, even if the initial reason for the treatment interruption was unrelated or unavoidable (e.g., travel for work or funeral), and ultimately lead to weakened sense of connection to HIV care [38]. Initial findings from the PCC study documented an adjusted 16.9% decrease in visits with a poor client experience (23.3% versus 3.5%) and an adjusted 5.9% increase in overall retention (80.2% versus 83.6%), but these estimates do not disentangle prevention of initial TIs and strengthening reengagement [45]. The current findings provide mechanistic insight into the parent PCC trial results and why improving the person-centeredness of care experience may lead to improved outcomes even after experiencing a treatment interruption, extending our current understanding of the mechanics of disengagement. By enhancing care experiences, rates of return may improve by reducing fear, stigma, or guilt for their care lapse, and a positive return experience and an ongoing caring environment can reduce subsequent TIs, fostering a virtuous cycle of sustained engagement [38,43]. Our results suggest that preventing repeat TIs may be the stronger driver of improved outcomes compared to improving return. Overall, these findings provide rigorous empirical quantitative evidence that strengthens our existing theoretical models of disengagement, and moves beyond simply being informed by theory to producing research that also informs and advances it [51].

This study offers crucial perspectives on strategies to improve outcomes among individuals with TIs, a population with enduring challenges to viral suppression despite improvements in HIV treatment programs over the past decade [8–17,20,24,30–34,36,37]. Existing evidence clearly identifies multiple structural, psychosocial, and clinic-based barriers that individuals encounter, including transportation difficulties, long wait times, and stigma [22–28]. Many of these barriers may be amenable to existing interventions such as restructuring care delivery to increase access (e.g., longer medication refills, differentiated service delivery) or implementing peer support and counseling programs [34,35]. However, there is also a high prevalence of existing or unexpected life circumstances—such as work or family obligations, geographic mobility, and social circumstances—that often make TIs unavoidable despite best efforts [26–28]. This reality underscores the critical importance of strategies that accommodate these circumstances to successfully reengage individuals after care lapses occur [34,35]. While tracking and tracing remain the mainstay of reengagement interventions and continue to be important, our evidence highlights the fundamental role of cultivating a friendly and safe healthcare environment to which individuals want to return. Importantly, the PCC approach is a generalized strategy that did not depend on extensively tailored structural interventions, but still had effects that were generally consistent across various subpopulations with evidence from the primary analysis also suggesting benefits may be greatest among those who have worse experiences [45]. By focusing on client-provider relationships, health systems can create supportive environments for clients even when underlying circumstances make some care lapses inevitable. Moreover, successful tracing is not always possible; individuals may be unreachable or highly mobile. PCC principles can synergize with and augment tracking and tracing efforts in two ways: first, by creating a welcoming environment that motivates autonomous return to care, and second, by facilitating connection and rapport with clients during tracing encounters. This study demonstrates that in public health settings, integrating person-centered behaviors to improve interpersonal dynamics is both a feasible and effective strategy to improve clinical outcomes after TIs, thus supporting the individuals who need it most.

Finally, these findings have particular salience given the current changes in HIV program funding and evolving priorities for health systems globally. As programs face resource constraints and shifting donor landscapes, reports indicate that support for traditional tracking and tracing activities is being particularly affected [58]. In this context, fostering feelings of trust and being valued in clients may help mitigate some of the impacts of compromised care delivery infrastructure, such as reduced outreach capacity, longer wait times, shorter medication refills, or delayed viral load monitoring. When structural aspects of care are constrained, the quality of interpersonal interactions becomes even more critical in maintaining patient engagement and therapeutic relationships. These findings suggest that investments to normalize PCC approaches could yield particularly high returns during periods of system strain, offering a potentially sustainable and cost-effective buffer against the disengagement that often accompanies deteriorating service conditions. Nevertheless, the PCC intervention procedures required sustained effort during the study, and the sustainability of effects and what the optimized package for integration into national programs is not yet known. By strengthening the client-provider relationship, health systems can build resilience that helps maintain engagement even as other programmatic resources become limited, but additional understanding on the cost-effectiveness and sustainability is still needed.

This study has several limitations. First, this was not a prespecified analysis, and, although the study design lends itself to a causal interpretation [59], there is possibility for increased type I error rate due to the post-hoc nature of the analysis and multiple outcomes. Still, the underlying hypothesis was driven by existing theoretical frameworks of disengagement, providing strong *a priori* justification. Second, the stepped-wedge design has inherent limitations for secular trends and outcomes that unfold over longer periods of time. In particular, the study design led to unequal observation time between groups, and also necessitated significant restriction of the sample to allow for unbiased analysis of binary outcomes. Importantly, each analysis cohort was epidemiologically robust and balanced, and multiple sensitivity analyses with various specifications, including one restricted to Groups 1 and 4 that allowed for concurrent and continuous periods under intervention or control conditions, yielded consistent results in terms of magnitude and direction. Not all sensitivity analyses met conventional thresholds for significance, but this is not unexpected given that the parallel-cluster randomized specification does not benefit from within-clinic comparison (i.e., pre- versus post-intervention), which substantially reduces precision and power particularly with higher intraclass correlations. Thus, the fact that there were consistent results despite different specifications indicates the robustness of the findings. Third, this analysis only includes individuals who had a treatment interruption and does not capture the intervention’s effect on preventing initial interruptions. If anything, this would likely bias results against the intervention, as individuals who still had TIs in intervention facilities may represent a more challenging population in whom interruptions were harder to prevent. Still, selection bias could remain despite adjustment. Furthermore, we adjusted for schedule appointment intervals to limit bias from selection, but there is also possibility that it has a mediating effect for the intervention (prior analyses would suggest this is not the case). Fourth, multistate analyses follow the Markov assumption that transition probabilities depend only on an individual’s current state and not prior care history. However, we addressed these limitations by defining care states that specifically incorporated prior care history (e.g., initial versus repeat TI), and also reset time zero for analyses specifically examining outcomes after return. Fifth, Schoenfeld residual plots indicated some violations of the proportional hazards assumption (particularly in the early periods), and as a result of this along with competing events (deaths, official transfers), Cox proportional hazards models estimate time-averaged cause-specific hazard ratios. Still, these results aligned with other analyses and should be interpreted in the context of cumulative risk from the multistate analysis. Sixth, electronic health record data may have limitations in identifying TIs due to incomplete data capture. We used imputation based on contextual knowledge of how appointment dates are generally assigned, but this could also lead to misclassification and reduced uncertainty. Given the randomized design, however, misclassification is unlikely to differ systematically across study arms, and results were also unchanged in a sensitivity analysis restricted to only those with no missing appointment data. Finally, there is limited understanding of transfers to other facilities, as some individuals may have sought care elsewhere. Even so, returning to the same clinic represents an endorsement of care quality, and preliminary evidence from the parent PCC trial suggests decreased transfers in intervention facilities [45], supporting the interpretation that the intervention improved care engagement rather than simply shifting care location.

This study demonstrates that targeting healthcare worker behaviors to create a warmer and more welcoming environment improved both return rates and retention after TIs, thereby increasing the overall proportion of individuals in care. Informed by theory-driven understanding of disengagement, our results demonstrate that changing the person-centeredness of the care environment can mitigate the impact of TIs, even where life circumstances make sustained care-seeking challenging. These impacts were implemented at essentially scale (i.e., conducted at clinics serving ~180,000 clients) and affected at least 56,000 individuals with TIs across 24 clinics in Zambia, potentially translating to ~5,000 more individuals in care after TIs. In an era of constrained resources and reduced support for tracking and tracing, PCC offers a sustainable, feasible, and effective strategy that creates healthcare environments to which individuals want to return—particularly important for individuals with TIs, a population that has proven difficult to serve. Future work should consider integrating these principles of person-centeredness across varying touchpoints where care disruptions can be mitigated, including outreach encounters, counseling sessions, and general care delivery, to maximize the transformative potential of person-centered approaches in HIV care.

## Supporting information

S1 Appendix Supplementary Appendix. (DOCX)

S1 Checklist CONSORT checklist - for more information please see: https://www.consort-spirit.org/ - CONSORT. (DOCX)

S1 Protocol PCC trial protocol. (DOCX)

S1 File Code PCC reengagement multistate data setup and analysis – stata. (DO)

S2 File Code run PCC reengage multistate – R. (R)

## Abbreviations

aHR: adjusted hazard ratio
aRR: adjusted risk ratio
CIs: confidence intervals
CIDRZ: Centre for Infectious Disease Research in Zambia
EHR: electronic health records
IRB: Institutional Review Boards
NGO: nongovernmental organization
MOH: Ministry of Health
PCC: person-centered care
PLWH: people living with HIV
RD: risk difference
TIs: treatment interruptions
UAB: University of Alabama at Birmingham.
