# Supplementary material for: Effect of a person-centered care intervention on return and sustained reengagement after treatment interruptions from HIV care in Zambia: A post-hoc analysis of a stepped-wedged cluster randomized trial
Source: PLoS Med. 2026 Sep 1;23(9):e1005052. doi: 10.1371/journal.pmed.1005052 (PMC13533374; doi:10.1371/journal.pmed.1005052)
Supplement: Supplementary file 1 [file pmed.1005052.s001.docx]

***Effect of a Person-Centered Care Intervention on Return and Sustained Reengagement After Treatment Interruptions from HIV Care in Zambia: A Post-Hoc Analysis of a Stepped-Wedged Cluster Randomized Trial***

**S1 APPENDIX**

**Fig A.** Theory Underlying Effect of PCC on Reengagement……………..…….………..………….………..………2

**Fig B.** Stepped wedge cluster randomized design ……………..…….…….……………..…………………..………3

**Fig C.** PCC Multicomponent Strategy.…………………….……….………….……………...……….………….…….4

**PCC Intervention description**………………………………………………………………….….……..………..…....5

**Fig D.** Direct Acyclic Graph underlying Analysis Plan. ………………...…………………….….……..………..…....8

**Table A.** Post-Hoc Sensitivity Analyses of Minimal Detectable Effect Sizes Based on Known Constraints……...9

**Table B.** Participants by Group and Period………………...……………….….……..………..….........................10

**Table C.** Percent of Visits with Missing Appointment Dates………….….……..………..…................................11

**Table D.** Healthcare Workers Trained in PCC Across Entire Facility ………………….………..………………....12

**Fig E.** Frequency of Mentor Visits………….….……..………..…........................................................................13

**Table E.** Top five most reported mentoring activities……………………………………...…………….……….....14

**Table F.** Crude Proportions in Individual and Composite Care States after Initial Treatment Interruption and after Return from Multistate Analyses…………………………………………………………………………………..15

**Table G.** Number under observation at varying timepoints in multistate analyses…………………….…………..17

**Fig F.** Transition Hazards for Return and Repeat Treatment Interruption by Intervention Status. …………......18

**Fig G.** Scaled Schoenfeld Residual Plots for Time-to-Event Analyses Hazards. ………………………….……..19

**Table H.** Event Rates by Cluster……………………………………………………………………………...………..20

**Table I.** Participant Characteristics of those Included and Restricted from Analyses for Binary Outcomes…21

**Table J.** Sensitivity Analyses of Crude Effects of PCC Intervention from Multistate Analyses……………….23

**Table K** Sensitivity Analyses of Effect of PCC Intervention on Return to Care and Retention from Adjusted Regression Models………………………..……………………...………………………….....…………………...…..24

**Fig H.** Forest Plot of Effect of PCC Intervention Stratified by Client Subgroups……………….………………….26

**Table L.** Effect of PCC Intervention on Return to Care and Retention Stratified by Study Period…..……….27

**Analysis Code Description**…..………………………………………………………………………………….…….28

**Fig A. Theory Underlying Effect of PCC on Reengagement.** Adapted from Norma Ware’s theory of disengagement, these figures demonstrate on baseline poor client-provider interactions can undermine both return to clinic and sustained reengagement after returning by fostering fear, guilt, disconnection, and, ultimately, reluctance to return to care. By strengthened person-centered provider behaviors prior to a treatment interruption, the PCC intervention can mitigate any fears of returning and foster ongoing connection with the clinic, thereby increasing both return to care and retention after return.

**Fig B. Stepped wedge cluster randomized design for PCC Trial.** The intervention was assigned in random sequence to four groups of clinics during each of four temporal periods. Empty white boxes represent the control status while the green represent the intervention status during that Period and Group. Periods were planned to be six months each, but Period 2 (started in February 2020) coincided with regional health emergency related to COVID-19 and was extended due to health systems challenges.

**Fig C. PCC Multicomponent Strategy.** We see the multicomponent intervention as operating through three pathways involving capability, opportunity and motivation. The immediate outcome was changes in health care worker behaviour, which then was thought to influence the client experience, client retention and clinical outcomes.

**Intervention Description**

Our theory of change for the PCC intervention integrated perspectives from health promotional and behavior changes models, including PRECEDE and COM-B^1-3^. These frameworks suggest that strategies must target multiple mechanisms to successfully foster change in provider practice related to PCC behaviors. Specifically, we sought to develop and intervention that targeted predisposing or capability factors such as knowledge and skills in delivering PCC, enabling or opportunity factors such as awareness and understanding of gaps in current client experience, and reinforcing or motivating factors to improve motivation to deliver more person-centered care. Based on underlying theory of these frameworks, addressing these three underlying determinants of behavior is necessary and sufficient to effect behavior change; that is, addressing a single determinant is unlikely to be successful on its own, but also recognizing that facilities and individuals may differ in the degree to which capability, opportunity, and motivation must be supported. Thus, the multicomponent PCC intervention included: 1) training and practice facilitation/mentorship on delivery of person-centered care, 2) systematic measurement and feedback of client experience metrics, and 3) a small facility-level reputational incentive for the best performing facilities.

As part of the intervention development process and prior to implementing the trial, we utilized Human Centred Design (HCD) approaches to co-create and tailor our intervention to the local context. This involved engaging frontline HCW, provincial, and district management staff in co-creation to develop and refine intervention components, including the person-centred care curriculum for HCWs (4). We learnt from the HCD workshops that client-centeredness principles (e.g., understanding the whole person, two-way communication) resonated with HCW but met resistance with some HCWs such as time constraints and beliefs that punitive measures improve client compliance (4). Through these HCD insights, we revised our intervention components to improve acceptability, appropriateness, and feasibility of PCC practices. The changes included: 1) incorporating the HCW experience in our theory of change, 2) integrating training, mentoring, and data collection components specifically targeting HCW experience to help HCWs feel visible, appreciated, and accountable (e.g., a module on HCW stress and burnout), 3) adding an additional 1-day facility workshop six weeks after curriculum training to support early synthesis of experiences applying PCC practices, and 4) integrated dedicated mentorship time for data comprehension^4^.

| **Table. Specification of intervention strategies using the Proctor framework.** | |
| --- | --- |
| **Domain** | **Strategy** |
| Actor | - Non- punitive Mentor who provides training, mentorship and coaching |
| Action | - Provides training and ongoing mentorship on PCC to HCW - Supports use of client experience data - Incentives for best performing facilities |
| Action Target | - All Lay and Professional HCWs trained in PCC - All cleaners and security guards trained in PCC |
| Behavioural Target | - Knowledge and skills to deliver person-centred care - Increased salience, motivation, and identification of opportunities to deliver more person-centred care - Increased of PCC behaviors, including being more welcoming, emphathy, improved communication, shared decision making, and leveraging discretionary power to better accommodate client needs. |
| Temporality | - Training HCW on PCC at 0m for Group 1, 7m for Group 2, 13m for group 3 and 19m for Group 4 - Mentorship begins immediately after first batch of HCWs trained in PCC - Synthesis meeting 6 weeks after training - Client experience data review meetings every 3 months - Incentive for best performing facility every 6 months |
| Dose | - PCC training done once for two days for each HCW - Weekly mentorship visits to facility till study end |

Training and Facilitation: The PCC intervention started with a two-day interactive training program that focused on strategies to improve the quality of interactions, included modules on PCC principles, client-provider communication, and stress management. Trainings were held outside the facility and targeted all HCWs (i.e., lay health workers, counsellors, medical officers, clinical officers, nurses, pharmacists, data managers) as well as facility support staff (e.g., security guards, cleaners) at the facility. The underlying premise was that interactions with any facility staff could influence the client experience and that HCWs commonly transfer between departments. After training was complete, study clinical mentors (i.e., 14 nurses and clinical officers trained in practice facilitation for person-centered care that oversaw 2-3 facilities each) provided ongoing support at facilities through approximately twice weekly mentorship facility visits throughout the intervention. Mentorship visits involved short lectures, role plays, and facilitated group discussion sessions that were tailored based on mentor assessment of facility needs and were delivered in a supportive manner that recognized the challenges faced by HCWs. During COVID-19 periods, trainings occurred in smaller groups (e.g., 5-10 HCWs rather than 40 HCW at a time).

Measurement and Feedback of Client Experience: We systematically measured client experience using exit interviews and short messaging service (SMS) surveys. A twelve-item exit survey was administered in-person to every *n*^th^ client leaving the facility after an HIV care visit, and five-item SMS surveys were sent within three days of a visit (with reminders send 1-, 3-, and 7-days later) to anyone with a mobile phone who expressed their willingness to participate. Surveys were based on ones developed by Wachira et al^5^ and assessed client perception and reports of satisfaction with services, HCW attitude, communication, and timeliness of services. These data were summarised in a user-friendly dashboard (Figure) and presented to facilities during quarterly data review meetings. During meetings, clinical mentors facilitated discussion and review of client experience data and trends over time, which was then used to identify, prioritize, or adjust efforts to improve client experience. These meetings provided HCWs opportunities to review and respond to gaps in client experience at their facilities. As the study progressed, responsibility for meeting facilitation gradually transitioned to the facility staff. These meetings and activities were intended to synergize and integrate with existing QI MOH QI practices and take advantage of existing QI champions. During COVID-19 periods, data review meetings were held with smaller groups.

**Figure. Data feedback dashboard.** This is an example of the client experience dashboard shared with the facilities at data review meetings. We used both SMS survey results and Exit survey results for the dashboard. For the first two indicators our aim was to get as close as possible to 100% and for the other five, we encouraged facilities to work towards getting to zero as close as possible.

Reputational incentive: To further motivate efforts, we offered recognition and provided token gifts of nominal value as facility-level incentives to be used at their discretion for the best performing facility ($75), second best performing facility ($50), and most improved facility ($25) on a biannual basis. A floating trophy as an award for good performance every six months was awarded to different facilities in each group rather than being kept by one facility. HCWs were also awarded a curriculum training certificate after completion of PCC curriculum training. There were no incentives to clients.

**Bibliography**

1. Crosby R, Noar SM. What is a planning model? An introduction to PRECEDE-PROCEED. J Public Health Dent [Internet]. 2011 Jan 1;71(s1):S7–15. Available from: <https://doi.org/10.1111/j.1752-7325.2011.00235>.
2. Green L, Kreuter M. Green LW, Kreuter MW. Health Program Planning: An Educational and Ecological Approach. 4th Edition. New York: McGraw-Hill, 2005. 2005 [cited 2023 Aug 28];408–30. Available from: <https://www.researchgate.net/publication/301749054_Green_LW_Kreuter_MW_Health_Program_Planning_An_Educational_and_Ecological_Approach_4th_Edition_New_York_McGraw-Hill_2005>.
3. Michie S, van Stralen MM, West R. The behaviour change wheel: A new method for characterising and designing behaviour change interventions. Implementation Science [Internet]. 2011;6(1):42. Available from: <https://doi.org/10.1186/1748-5908-6-42>.
4. Beres LK, Simbeza S, Holmes CB, Mwamba C, Mukamba N, Sharma A, et al. Human-Centered Design Lessons for Implementation Science. JAIDS Journal of Acquired Immune Deficiency Syndromes [Internet]. 2019 Dec [cited 2019 Nov 25];82:S230–43. Available from: <http://insights.ovid.com/crossref?an=00126334-201912003-00010>
5. Wachira J, Middlestadt S, Reece M, Peng CYJ, Braitstein P. Psychometric assessment of a physician-patient communication behaviors scale: The perspective of adult HIV patients in Kenya. AIDS Res Treat. 2013;2013.

**Fig D. Direct Acyclic Graph underlying Analysis Plan.** The directed acyclic graph (DAG) shows that PCC exposure is independent conditional on clinic and study period due to stepped-wedge cluster randomized design. However, this analysis was restricted to those who had a treatment interruption, which could open up selection/collider bias as the PCC intervention may have reduced initial treatment interruptions. Scheduled appointment interval was not conceptualized as mediator of PCC intervention based on contextual knowledge and required adjustment due to its association with initial treatment interruptions and outcomes (this would have required adjustment including if scheduled appointment were conceptualized as a mediator).

| **Table A. Post-Hoc Sensitivity Analyses of Minimal Detectable Effect Sizes Based on Known Constraints** | | | | | | |
| --- | --- | --- | --- | --- | --- | --- |
|  | N Clusters | Total Sample | Baseline Risk | ICC | Minimal Detectable Risk Difference | Minimal Detectable Hazard Ratio |
| Stepped Wedge Design – Return | 24 | 128832 | 0.65 | 0.05 | 0.013 | 1.04 |
| Stepped Wedge Design – Repeat Treatment Interruption | 24 | 86304 | 0.55 | 0.05 | -0.017 | 0.95 |
| Parallel Cluster – Return | 16 | 80752 | 0.65 | 0.05 | 0.140 | 1.49 |
| Parallel Cluster – Repeat Treatment Interruption | 16 | 55984 | 0.55 | 0.05 | -0.151 | 0.63 |

Sample Size Considerations: The parent PCC trial was designed with 24 clusters in attempts to carry out the intervention at the maximal scale possible under financial and logistical constraints. Similar to procedures for leveraging the EHR in the parent trial, sample size for this analysis was based on practical and not statistical considerations, and we leveraged all available data in the EHR at these 24 sites during the study period. We conducted post-hoc sensitivity analysis of minimal detectable risk differences (based on power 0.8 and alpha 0.05) to contextualize our ability to identify differences between intervention and control conditions using known parameters from the available data, including sample size, baseline risk in the control arm, and clinic ICC based on results from primary trial analyses. We estimated the minimal detectable risk difference under the various design specifications, and transformed into minimal detectable hazard ratio assuming constant hazard)

| **Table B. Participants by Group and Period** | | | | | |  | Totals By Group | |
| --- | --- | --- | --- | --- | --- | --- | --- | --- |
|  |  | Period 1 | Period 2 | Period 3 | Period 4 |  | Control | Intervention |
|  | Group 1 | 19,248 | 15,630 | 4,677 | 2,529 |  | 0 | 42,084 |
|  | Group 2 | 7,934 | 6,969 | 2,189 | 1,168 |  | 7,934 | 10,326 |
|  | Group 3 | 12,666 | 8,055 | 2,831 | 1,692 |  | 20,721 | 4,523 |
|  | Group 4 | 16,326 | 16,895 | 7,795 | 2,306 |  | 41,016 | 2,306 |
|  |  |  |  |  |  |  |  |  |
| Totals by Period | Control | 36,926 | 24,950 | 7,795 | 0 |  | 69,671 | - |
|  | Intervention | 19,248 | 22,599 | 9,697 | 7,695 |  | - | 59,239 |

| **Table C. Percent of Visits with Missing Appointment Dates** | | | | |
| --- | --- | --- | --- | --- |
|  | Period 1 | Period 2 | Period 3 | Period 4 |
| Group 1 | 1.40% | 1.96% | 0.27% | 0.14% |
| Group 2 | 1.34% | 2.86% | 0.50% | 0.23% |
| Group 3 | 1.45% | 1.30% | 0.34% | 0.24% |
| Group 4 | 1.27% | 5.60% | 0.62% | 0.16% |

| Table D. Healthcare Workers Trained in PCC Across Entire Facility | | | |
| --- | --- | --- | --- |
| Cadre | **Number trained in PCC** | **Number of staff in all sites** | **Percentage trained** |
| Medical officer, Dental Surgeon | 35 | 86 | 40.7% |
| Nurse | 883 | 1486 | 59.4% |
| Data/M&E, Biomedical Scientist | 167 | 271 | 61.6% |
| Counselor/Treatment supporter | 841 | 1272 | 66.1% |
| Clinical Officer, Medical Licentiate | 165 | 246 | 67.1% |
| Other | 42 | 62 | 67.7% |
| General worker e.g. security, drivers, housekeeping | 153 | 198 | 77.3% |
| Human Resource, cashiers, clerks, admin staff, public relations | 137 | 175 | 78.3% |
| Community Liaison Officer/ Community Health Worker | 65 | 81 | 80.3% |
| Pharmacist/ Pharmacy Technologists | 79 | 84 | 94.1% |
| Total | **2567** | **3961** | 64.8% |

**Fig E. Frequency of Mentor Visits** Frequency of visits that mentors made to health facilities weekly. Mentors visited facilities at least once a week throughout the study (facilities intervention phase). However, there were more visits in Group 1 & 2 and this is on account of increased support that PCC study mentors rendered to facilities in response to COVID-19 between May 2020 and August 2020.

| Table E. Top five most reported mentoring activities as a proportion of all reported activities. | | | | |
| --- | --- | --- | --- | --- |
|  | Percent of total activities per period | | | |
| Top 5 commonly reported mentoring activities | Period 1 | Period 2 | Period 3 | Period 4 |
| Relationship building | 29% | 31% | 33% | 31% |
| One-on-one HCW observation and mentoring | 25% | 17% | 28% | 24% |
| Supporting facility context improvements (facility-prioritized system-level changes driven by data review) | 10% | 12% | 5% | 8% |
| Meeting with facility leaders (Overall In-Charge, ART In-Charge or Departmental Heads) | 0% | 10% | 6% | 7% |
| Service to support facility (side-by-side work) | 2% | 3% | 2% | 3% |

Footnote: Mentors recorded their activities during mentorship visits to the health facilities in Mentor Activity Logs. These are the 'Top five most reported mentoring activities' (out of 17 main activities). Mentors spent most of their time in 'relationship building' and 'one-on-one mentoring' and this points to the importance of interpersonal interaction.

**Table F. Crude Proportions in Individual and Composite Care States after Initial Treatment Interruption and after Return from Multistate Analyses**

|  | **30 days** | | | **90 days** | | | **180 days** | | | **365 days** | | | **540 days** | | |
| --- | --- | --- | --- | --- | --- | --- | --- | --- | --- | --- | --- | --- | --- | --- | --- |
| **Care State** | Cont, % (95% CI) | Interv, % (95% CI) | RD, % (95% CI) | Cont, % (95% CI) | Interv, % (95% CI) | RD, % (95% CI) | Cont, % (95% CI) | Interv, % (95% CI) | RD, % (95% CI) | Cont, % (95% CI) | Interv, % (95% CI) | RD, % (95% CI) | Cont, % (95% CI) | Interv, % (95% CI) | RD, % (95% CI) |
| **After Initial Treatment Interruption (n=128,910)** | | | | | | | | | | | | | | | |
| Treatment Interruption | 83.9  (83.6, 84.2) | 85.1  (84.8, 85.4) | 1.2  (0.8, 1.6) | 49.6  (49.2, 50.0) | 47.5  (47.1, 47.9) | -2.1  (-2.7,  -1.6) | 37.6  (37.3, 38.0) | 34.5  (34.1, 34.9) | -3.2  (-3.7, -2.6) | 31.0  (30.6, 31.4) | 26.2  (25.8, 26.6) | -4.8  (-5.3, -4.3) | 29.1  (28.8, 29.5) | 24.4  (24.0, 24.8) | -4.7  (-5.3, -4.2) |
| Return, No Repeat Treatment Interruption | 16.0  (15.7, 16.2) | 14.7  (14.4, 15.0) | -1.3  (-1.7,  -0.9) | 49.4  (49.0, 49.7) | 51.5  (51.1, 51.9) | 2.1  (1.6, 2.7) | 51.7  (51.3, 52.1) | 55.5  (55.1, 55.9) | 3.8  (3.2, 4.4) | 35.0  (34.6, 35.5) | 44.3  (43.8, 44.7) | 9.2  (8.6, 9.8) | 22.5  (22.0, 23.1) | 38.1  (37.6, 38.5) | 15.5  (14.8, 16.3) |
| Repeat Treatment Interruption | 0.0  (0.0, 0.0) | 0.0  (0.0, 0.0) | 0.0  (0.0, 0.0) | 0.5  (0.5, 0.6) | 0.4  (0.3, 0.5) | -0.1  (-0.2,  -0.1) | 8.5  (8.3, 8.8) | 7.8  (7.5, 8.0) | -0.8  (-1.1, -0.4) | 16.2  (15.8, 16.6) | 12.2  (11.9, 12.5) | -4.0  (-4.5, -3.5) | 25.0  (24.4, 25.7) | 12.9  (12.6, 13.3) | -12.1  (-12.8,  -11.4) |
| Return after Repeat Treatment Interruption | 0.0  (0.0, 0.0) | 0.0  (0.0, 0.0) | 0.0  (0.0, 0.0) | 0.1  (0.1, 0.1) | 0.1  (0.0, 0.1) | 0.0  (0.0, 0.0) | 1.4  (1.3, 1.5) | 1.4  (1.3, 1.5) | 0.0  (-0.1, 0.2) | 16.4  (16.1, 16.8) | 15.9  (15.6, 16.2) | -0.5  (-1.0, -0.1) | 21.4  (20.8, 21.9) | 22.6  (22.2, 23.0) | 1.2  (0.5, 1.9) |
| Official Transfer | 0.1  (0.1, 0.1) | 0.1  (0.1, 0.2) | 0.1  (0.0, 0.1) | 0.3  (0.2, 0.3) | 0.3  (0.3, 0.4) | 0.0  (0.0, 0.1) | 0.4  (0.4, 0.5) | 0.5  (0.4, 0.6) | 0.1  (0.0, 0.1) | 0.8  (0.7, 0.9) | 0.9  (0.8, 1.0) | 0.1  (0.0, 0.2) | 1.2  (1.1, 1.3) | 1.2  (1.1, 1.3) | 0.0  (-0.2, 0.2) |
| Death | 0.1  (0.0, 0.1) | 0.1  (0.1, 0.1) | 0.0  (0.0, 0.1) | 0.1  (0.1, 0.2) | 0.2  (0.2, 0.3) | 0.1  (0.0, 0.1) | 0.3  (0.2, 0.3) | 0.3  (0.3, 0.4) | 0.1  (0.0, 0.1) | 0.5  (0.5, 0.6) | 0.6  (0.5, 0.7) | 0.1  (0.0, 0.2) | 0.7  (0.6, 0.8) | 0.8  (0.7, 0.9) | 0.1  (-0.1, 0.2) |
| **Any Return** | **16.0**  **(15.7, 16.2)** | **14.7**  **(14.4, 15.0)** | **-1.3**  **(-1.7,**  **-0.9)** | **50.0**  **(49.6, 50.4)** | **52.0**  **(51.6, 52.4)** | **2.0**  **(1.4, 2.6)** | **61.7**  **(61.3, 62.0)** | **64.7**  **(64.3, 65.1)** | **3.0**  **(2.5, 3.6)** | **67.7**  **(67.3, 68.0)** | **72.3**  **(71.9, 72.7)** | **4.6**  **(4.1, 5.2)** | **68.9**  **(68.6, 69.3)** | **73.6**  **(73.2, 74.0)** | **4.7**  **(4.1, 5.2)** |
| **In Care** | **16.0**  **(15.7, 16.2)** | **14.7**  **(14.4, 15.0)** | **-1.3**  **(-1.7,**  **-0.9)** | **49.4**  **(49.1, 49.8)** | **51.6**  **(51.2, 52.0)** | **2.1**  **(1.6, 2.7)** | **53.1**  **(52.7, 53.5)** | **56.9**  **(56.5, 57.3)** | **3.8**  **(3.2, 4.4)** | **51.5**  **(51.0, 51.9)** | **60.1**  **(59.7, 60.6)** | **8.7**  **(8.0, 9.3)** | **43.9**  **(43.3, 44.6)** | **60.7**  **(60.2, 61.2)** | **16.7**  **(15.9, 17.6)** |
| **After Return (n=86,333)** | | | | | | | | | | | | | | | |
| Return, No Repeat Treatment Interruption | 99.5  (99.4, 99.5) | 99.3  (99.2, 99.4) | -0.2  (-0.3,  -0.1) | 96.4  (96.2, 96.6) | 96.7  (96.5, 96.9) | 0.3  (0.0, 0.5) | 68.7  (68.2, 69.2) | 73.5  (73.0, 73.9) | 4.8  (4.1, 5.4) | 42.6  (42.0, 43.2) | 53.8  (53.3, 54.3) | 11.2  (10.4, 12.1) | 24.3  (23.5, 25.2) | 45.5  (44.9, 46.1) | 21.2  (20.1, 22.2) |
| Repeat Treatment Interruption | 0.0  (0.0, 0.0) | 0.0  (0.0, 0.0) | 0.0  (0.0, 0.0) | 2.5  (2.3, 2.7) | 2.1  (1.9, 2.2) | -0.4  (-0.6,  -0.2) | 20.8  (20.4, 21.3) | 15.7  (15.3, 16.0) | -5.1  (-5.7, -4.6) | 24.8  (24.3, 25.4) | 15.4  (14.9, 15.8) | -9.5  (-10.2,  -8.7) | 40.2  (39.2, 41.2) | 18.9  (18.2, 19.5) | -21.3  (-22.5,  -20.1) |
| Return after Repeat Treatment Interruption | 0.0  (0.0, 0.0) | 0.0  (0.0, 0.0) | 0.0  (0.0, 0.0) | 0.4  (0.4, 0.5) | 0.4  (0.3, 0.5) | -0.1  (-0.1, 0.0) | 9.5  (9.2, 9.8) | 9.7  (9.4, 10.0) | 0.2  (-0.3, 0.6) | 30.8  (30.2, 31.4) | 28.9  (28.4, 29.4) | -1.9  (-2.7, -1.1) | 32.8  (31.9, 33.7) | 33.1  (32.4, 33.8) | 0.3  (-0.8, 1.4) |
| Official Transfer | 0.5  (0.4, 0.6) | 0.7  (0.6, 0.7) | 0.1  (0.0, 0.3) | 0.6  (0.5, 0.6) | 0.7  (0.6, 0.8) | 0.2  (0.1, 0.3) | 0.8  (0.7, 0.9) | 0.9  (0.9, 1.0) | 0.2  (0.0, 0.3) | 1.3  (1.2, 1.4) | 1.4  (1.3, 1.5) | 0.1  (0.0, 0.3) | 1.9  (1.6, 2.1) | 1.8  (1.6, 1.9) | -0.1  (-0.4, 0.2) |
| Death | 0.0  (0.0, 0.0) | 0.1  (0.0, 0.1) | 0.0  (0.0, 0.1) | 0.1  (0.1, 0.1) | 0.1  (0.1, 0.2) | 0.0  (0.0, 0.1) | 0.2  (0.2, 0.3) | 0.3  (0.2, 0.3) | 0.0  (0.0, 0.1) | 0.5  (0.4, 0.6) | 0.5  (0.4, 0.6) | 0.0  (-0.1, 0.1) | 0.8  (0.7, 0.9) | 0.8  (0.7, 0.9) | 0.0  (-0.2, 0.1) |
| **Any Repeat Treatment Interruption** | **0.0**  **(0.0, 0.0)** | **0.0**  **(0.0, 0.0)** | **0.0**  **(0.0, 0.0)** | **3.0**  **(2.8, 3.1)** | **2.5**  **(2.3, 2.6)** | **-0.5**  **(-0.7,**  **-0.2)** | **30.3**  **(29.8, 30.8)** | **25.3**  **(24.9, 25.8)** | **-5.0**  **(-5.6, -4.3)** | **55.6**  **(55.0, 56.2)** | **44.3**  **(43.7, 44.8)** | **-11.4**  **(-12.2,**  **-10.6)** | **73.0**  **(72.1, 73.9)** | **52.0**  **(51.4, 52.6)** | **-21.0**  **(-22.1,**  **-19.9)** |
| **In Care after Return** | **99.5**  **(99.4, 99.5)** | **99.3**  **(99.2, 99.4)** | **-0.2**  **(-0.3,**  **-0.1)** | **96.8**  **(96.7, 97.0)** | **97.1**  **(96.9, 97.2)** | **0.2**  **(0.0, 0.5)** | **78.2**  **(77.7, 78.6)** | **83.1**  **(82.7, 83.5)** | **4.9**  **(4.4, 5.5)** | **73.4**  **(72.8, 74.0)** | **82.7**  **(82.2, 83.1)** | **9.3**  **(8.6, 10.0)** | **57.1**  **(56.1, 58.2)** | **78.6**  **(77.9, 79.2)** | **21.5**  **(20.2, 22.7)** |

Footnote: Main outcomes of interest highlighted in yellow.

| **Table G. Number under observation at varying timepoints in multistate analyses** | | | | | | |
| --- | --- | --- | --- | --- | --- | --- |
| **After Initial Treatment Interruption (n=128,910)** | | | | | | |
|  | **0 days** | **30 days** | **90 days** | **180 days** | **365 days** | **540 days** |
| Control | 69,671 | 67,574 | 60,832 | 54,036 | 36,433 | 18,171 |
| Intervention | 59,239 | 57,057 | 53,267 | 48,147 | 37,527 | 21,555 |
| Total | 128,910 | 124,631 | 114,099 | 102,183 | 73,960 | 39,726 |
| **After Return (n=86,333)** | | | | | | |
|  | **0 days** | **30 days** | **90 days** | **180 days** | **365 days** | **540 days** |
| Control | 47,150 | 35,121 | 34,384 | 28,438 | 13,266 | 4,324 |
| Intervention | 39,183 | 38,902 | 37,750 | 34,413 | 24,131 | 9,272 |
| Total | 86,333 | 74,023 | 72,134 | 62,851 | 37,397 | 13,596 |

**Fig F. Transition Hazards for Return and Repeat Treatment Interruption by Intervention Status.** This figure depicts the instantaneous hazard rates over time for A) Return to Care after initial treatment interruption, and B) Repeat Treatment Interruption after return.

**Fig G. Scaled Schoenfeld Residual Plots for Time-to-Event Analyses Hazards.** This figure depicts the scaled Schoenfeld residual plots for Cox proportional hazards analyses for A) Time to Return to Care after initial treatment interruption, and B) Time to Repeat Treatment Interruption after return.

**Table H. Event Rates by Cluster**

| **Clinic** | **Time to Return**  **(Event Rate per Person-Year)** | | **Time to Repeat Treatment Interruption**  **(Event Rate per Person-Year)** | |
| --- | --- | --- | --- | --- |
|  | **Control** | **Intervention** | **Control** | **Intervention** |
| G1C1 | - | 2.70 | - | 0.53 |
| G1C2 | - | 0.78 | - | 0.35 |
| G1C3 | - | 1.21 | - | 0.50 |
| G1C4 | - | 1.58 | - | 0.56 |
| G1C5 | - | 2.23 | - | 0.61 |
| G1C6 | - | 1.50 | - | 0.58 |
| G1C7 | - | 1.90 | - | 0.41 |
| G1C8 | - | 1.85 | - | 0.71 |
| G2C1 | 1.63 | 1.61 | 1.22 | 0.69 |
| G2C2 | 1.29 | 1.91 | 1.27 | 0.41 |
| G2C3 | 1.47 | 1.83 | 1.09 | 0.40 |
| G2C4 | 1.17 | 2.75 | 0.78 | 0.49 |
| G3C1 | 1.49 | 1.24 | 0.53 | 0.50 |
| G3C2 | 1.03 | 1.42 | 0.52 | 0.65 |
| G3C3 | 1.05 | 0.91 | 0.68 | 0.58 |
| G3C4 | 1.41 | 0.75 | 1.20 | 0.44 |
| G4C1 | 0.67 | 0.00 | 1.39 | 0.00 |
| G4C2 | 1.11 | 0.52 | 0.87 | 0.37 |
| G4C3 | 1.89 | 0.74 | 0.41 | 0.00 |
| G4C4 | 1.28 | 0.46 | 0.55 | 0.64 |
| G4C5 | 0.71 | 0.00 | 1.16 | 0.00 |
| G4C6 | 2.24 | 0.65 | 1.55 | 0.25 |
| G4C7 | 0.67 | 0.53 | 0.56 | 0.17 |
| G4C8 | 1.69 | 1.39 | 0.78 | 0.00 |

**Table I. Participant Characteristics of those Included in and Restricted from Analyses for Binary Outcomes**

|  |  | In Care 365 days after Treatment Interruptions | | | In Care 365 days after Return | | |
| --- | --- | --- | --- | --- | --- | --- | --- |
|  |  | Restricted (n=77323) | Included (n=51587) | Standardized Difference | Restricted (n=56245) | Included (n=30088) | Standardized Difference |
| Sex, n (%) | Female | 49491 (64.0%) | 33399 (64.7%) | 0.02 | 56245 | 30088 | 0.03 |
|  | Male | 27832 (36.0%) | 18188 (35.3%) |  | 36413 (64.7%) | 18997 (63.1%) |  |
| Median Age at Treatment Interruption, years (IQR) |  | 38 (31, 45) (n=77311) | 38 (31, 45) (n=51583) | 0.00 | 19832 (35.3%) | 11091 (36.9%) | 0.03 |
| Age at Treatment Interruption, n (%) | <25 years | 6819 (8.8%) | 4502 (8.7%) | 0.01 | 39 (32, 46) (n=56232) | 39 (32, 46) (n=30085) | 0.04 |
|  | 25-44 years | 51086 (66.1%) | 34019 (65.9%) |  | 4038 (7.2%) | 1908 (6.3%) |  |
|  | >45 years | 19406 (25.1%) | 13062 (25.3%) |  | 36534 (65.0%) | 19501 (64.8%) |  |
|  | Missing | 12 (<1%) | 4 (<1%) |  | 15660 (27.8%) | 8676 (28.8%) |  |
| Years on ART prior to treatment interruption, n (%) | <6m | 19794 (25.6%) | 11006 (21.3%) | 0.11 | 13 (<1%) | 3 (<1%) | 0.04 |
|  | 6m-2y | 15158 (19.6%) | 11408 (22.1%) |  | 8360 (14.9%) | 4026 (13.4%) |  |
|  | 2-5y | 16823 (21.8%) | 11766 (22.8%) |  | 11909 (21.2%) | 6636 (22.1%) |  |
|  | >5y | 25548 (33.0%) | 17407 (33.7%) |  | 13790 (24.5%) | 7511 (25.0%) |  |
| Scheduled Appointment Interval*, n (%) | 14 days | 3399 (4.4%) | 4104 (8.0%) | 0.32 | 22186 (39.4%) | 11915 (39.6%) | 0.19 |
|  | 30 days | 10067 (13.0%) | 5328 (10.3%) |  | 973 (1.7%) | 550 (1.8%) |  |
|  | 60 days | 2260 (2.9%) | 1651 (3.2%) |  | 4112 (7.3%) | 1981 (6.6%) |  |
|  | 90 days | 41214 (53.3%) | 31880 (61.8%) |  | 1453 (2.6%) | 827 (2.7%) |  |
|  | 120 days | 5517 (7.1%) | 3364 (6.5%) |  | 25506 (45.3%) | 15186 (50.5%) |  |
|  | 180 days | 14866 (19.2%) | 5260 (10.2%) |  | 3046 (5.4%) | 2479 (8.2%) |  |
| WHO Stage at Enrollment, n (%) | Stage 1 | 41679 (53.9%) | 26990 (52.3%) | 0.06 | 21155 (37.6%) | 9065 (30.1%) | 0.04 |
|  | Stage 2 | 8562 (11.1%) | 6277 (12.2%) |  | 28774 (51.2%) | 15056 (50.0%) |  |
|  | Stage 3 | 10314 (13.3%) | 7433 (14.4%) |  | 7061 (12.6%) | 4005 (13.3%) |  |
|  | Stage 4 | 761  (1.0%) | 664  (1.3%) |  | 9139 (16.2%) | 4892 (16.3%) |  |
|  | Missing | 16007 (20.7%) | 10223 (19.8%) |  | 705  (1.3%) | 443  (1.5%) |  |
| Marital Status, n (%) | Single | 10001 (12.9%) | 5657 (11.0%) | 0.07 | 10566 (18.8%) | 5692 (18.9%) | 0.07 |
|  | Married | 40965 (53.0%) | 27993 (54.3%) |  | 6639 (11.8%) | 2957 (9.8%) |  |
|  | Divorced | 8971 (11.6%) | 5495 (10.7%) |  | 30860 (54.9%) | 16657 (55.4%) |  |
|  | Widowed | 5582 (7.2%) | 3856 (7.5%) |  | 6160 (11.0%) | 3103 (10.3%) |  |
|  | Missing | 11804 (15.3%) | 8586 (16.6%) |  | 4365 (7.8%) | 2431 (8.1%) |  |
| Education, n (%) | None | 3697 (4.8%) | 2369 (4.6%) | 0.06 | 8221 (14.6%) | 4940 (16.4%) | 0.04 |
|  | Primary | 18563 (24.0%) | 13199 (25.6%) |  | 2858 (5.1%) | 1459 (4.8%) |  |
|  | Secondary | 38766 (50.1%) | 25390 (49.2%) |  | 14103 (25.1%) | 7877 (26.2%) |  |
|  | University | 4529 (5.9%) | 2550 (4.9%) |  | 27908 (49.6%) | 14551 (48.4%) |  |
|  | Missing | 11768 (15.2%) | 8079 (15.7%) |  | 3282 (5.8%) | 1561 (5.2%) |  |
| Facility Type, n (%) | Not Hospital-Based | 42651 (55.2%) | 26014 (50.4%) | 0.09 | 8094 (14.4%) | 4640 (15.4%) | 0.01 |
|  | Hospital-Based | 34672 (44.8%) | 25573 (49.6%) |  | 30214 (53.7%) | 16012 (53.2%) |  |
| Group, n (%) | 1 | 9977 (12.9%) | 32107 (62.2%) | - | 26031 (46.3%) | 14076 (46.8%) | - |
|  | 2 | 12832 (16.6%) | 5428 (10.5%) |  | 9106 (16.2%) | 21036 (69.9%) |  |
|  | 3 | 24602 (31.8%) | 642 (1.2%) |  | 10496 (18.7%) | 3113 (10.3%) |  |
|  | 4 | 29912 (38.7%) | 13410 (26.0%) |  | 16237 (28.9%) | 95 (0.3%) |  |
| Period, n (%) | 1 | 23608 (30.5%) | 32566 (63.1%) | - | 20406 (36.3%) | 5844 (19.4%) | - |
|  | 2 | 28577 (37.0%) | 18972 (36.8%) |  | 23315 (41.5%) | 19267 (64.0%) |  |
|  | 3 | 17443 (22.6%) | 49 (0.1%) |  | 25377 (45.1%) | 10821 (36.0%) |  |
|  | 4 | 7695 (10.0%) | 0 (0.0%) |  | 6713 (11.9%) | 0 (0.0%) |  |

Footnote: *Scheduled Appointment Interval either at the last visit prior to treatment interruption (After Treatment Interruption) or at the return visit (Among Returners). ART=antiretroviral therapy. Groups represent the clusters of clinics randomly assigned for intervention rollout at different time periods; Period represents the study period for the stepped-wedge design. Standardized differences not presented for Group and Period as these are imbalanced by design.

**Table J. Sensitivity Analyses of Crude Effects of PCC Intervention from Multistate Analyses**

| **After Initial Treatment Interruption** | | | | | | |
| --- | --- | --- | --- | --- | --- | --- |
|  | **Any Return at 365 days** | | | **In Care at 365 days after Treatment Interruption** | | |
|  | Control % (95% CI) | Intervention % (95% CI) | **Risk Difference, % (95% CI)** | Control % (95% CI) | Intervention % (95% CI) | **Risk Difference, % (95% CI)** |
| Main Analysis (n=128,910) | 67.7  (67.3, 68.0) | 72.3  (71.9, 72.7) | **4.6**  **(4.1, 5.2)** | 51.5  (51.0, 51.9) | 60.1  (59.7, 60.6) | **8.7**  **(8.0, 9.3)** |
| Restricted to Groups 1 and 4 (n=80,571) | 65.6  (65.1, 66.1) | 74.6  (74.1, 75.0) | **8.9**  **(8.3, 9.6)** | 49.1  (48.6, 49.7) | 60.6  (60.1, 61.1) | **11.5**  **(10.7, 12.3)** |
| Exclude individuals with any imputed apt intervals (n=120,853) | 65.4  (65.0, 65.8) | 71.1  (70.7, 71.5) | **5.7**  **(5.1, 6.2)** | 49.5  (49.0, 49.9) | 58.7  (58.2, 59.1) | **9.2**  **(8.6, 9.9)** |
| Treatment Interruption >90 days (n=91,660) | 52.3  (51.9, 52.8) | 59.0  (58.4, 59.5) | **6.7**  **(5.9, 7.4)** | 42.6  (42.1, 43.2) | 53.9  (53.3, 54.4) | **11.3**  **(10.5, 12.1)** |
| **After Return** | | | | | | |
|  | **Any Repeat Treatment Interruption** | | | **In Care at 365 days after Return** | | |
|  | Control % (95% CI) | Intervention % (95% CI) | **Risk Difference, % (95% CI)** | Control % (95% CI) | Intervention % (95% CI) | **Risk Difference, % (95% CI)** |
| Main Analysis (n=86,333) | 55.6  (55.0, 56.3) | 44.3  (43.7, 44.8) | **-11.4**  **(-12.2, -10.5)** | 73.4  (72.8, 74.0) | 82.7  (82.2, 83.1) | **9.3**  **(8.5, 10.1)** |
| Restricted to Groups 1 and 4 (n=55,978) | 58.7  (58.0, 59.5) | 50.7  (49.9, 51.3) | **-8.0**  **(-9.1, -7.1)** | 72.5  (71.8, 73.2) | 80.4  (79.8, 81.0) | **7.9**  **(7.0, 8.9)** |
| Exclude individuals with any imputed apt intervals (n=78,713) | 55.3  (54.6, 55.9) | 44.9  (44.3, 45.4) | **-10.4**  **(-11.3, -9.5)** | 73.0  (72.4, 73.6) | 82.2  (81.7, 82.6) | **9.1**  **(8.4, 9.9)** |
| Treatment Interruption >90 days (n=47,593) | 41.0  (40.0, 42.1) | 21.9  (21.3, 22.6) | **-19.1**  **(-20.3, -17.9)** | 73.8  (72.9, 74.8) | 87.2  (86.7, 87.8) | **13.4**  **(12.3, 14.5)** |

**Table K. Sensitivity Analyses of Effect of PCC Intervention on Return to Care and Retention from Adjusted Regression Models**

| **After Initial Treatment Interruption** | | | |
| --- | --- | --- | --- |
| **Time to Return** | **Adjusted Cause-Specific Hazard Ratio**  **(95% CI)** | **p-value** | **ICC** |
| Main Analysis (n=128,894) | 1.16  (1.12, 1.20) | <0.001 | 0.033 |
| Restricted to Groups 1 and 4 (n=80,566) | 1.33  (0.99, 1.80) | 0.061 | 0.044 |
| Exclude individuals with any imputed appointment intervals (n=120,838) | 1.17  (1.13, 1.21) | <0.001 | 0.051 |
| Treatment Interruption >60 days (n= 115,522) | 1.21  (1.17, 1.25) | <0.001 | 0.039 |
| Treatment Interruption >90 days (n=91,654) | 1.16  (1.11, 1.22) | <0.001 | 0.053 |
| **In Care at 365 days** | **Adjusted Risk Ratio**  **(95% CI)** | **p-value** | **ICC** |
| Main Analysis (n=51,583) | 1.19  (1.05, 1.35) | 0.008 | 0.031 |
| Restricted to Groups 1 and 4 (n=45,494) | 1.20  (1.04, 1.38) | 0.010 | 0.034 |
| Exclude individuals with any imputed appointment intervals (n= 48,331) | 1.20  (1.05, 1.38) | 0.009 | 0.033 |
| Treatment Interruption >60 days (n=41,748) | 1.22  (1.02, 1.44) | 0.026 | 0.052 |
| Treatment Interruption >90 days (n=28,627) | 1.36  (1.07, 1.74) | 0.012 | 0.063 |
| **After Return** | | | |
| **Time to Repeat Treatment Interruption** | **Adjusted Cause-Specific Hazard Ratio**  **(95% CI)** | **p-value** | **ICC** |
| Main Analysis (n=86,317) | 0.50  (0.45, 0.55) | <0.001 | 0.058 |
| Restricted to Groups 1 and 4 (n=55,973) | 0.69  (0.46, 1.02) | 0.066 | 0.072 |
| Exclude individuals with any imputed appointment intervals (n=78,698) | 0.52  (0.47, 0.58) | <0.001 | 0.052 |
| Treatment Interruption >60 days (n=71,578) | 0.47  (0.40, 0.56) | <0.001 | 0.077 |
| Treatment Interruption >90 days (n=47,588) | 0.34  (0.26, 0.44) | <0.001 | 0.068 |
| **In Care at 365 days after return** | **Adjusted Risk Ratio**  **(95% CI)** | **p-value** | **ICC** |
| Main Analysis (n=30,085) | 1.14  (1.05, 1.25) | 0.002 | 0.037 |
| Restricted to Groups 1 and 4 (n= 26,879) | 1.14  (1.04, 1.25) | 0.006 | 0.040 |
| Exclude individuals with any imputed appointment intervals (n= 27,354) | 1.13  (1.04, 1.23) | 0.005 | 0.035 |
| Treatment Interruption >60 days (n= 22,873) | 1.14  (1.04, 1.24) | 0.006 | 0.041 |
| Treatment Interruption >90 days (n= 12,624) | 1.07  (1.02, 1.12) | 0.011 | 0.055 |

Footnote: Analyses adjusted for study facility as shared frailty (Cox Models) or random effect (mixed effects Poisson models with robust variances) and study period as fixed effects. Analyses also included adjustment for sex and age (including sex*age interaction), time since ART initiation, scheduled appointment interval at either last visit or time of return, and facility type. Individuals with missing age were excluded from these analyses.

**Fig H. Forest Plot of Effect of PCC Intervention Stratified by Client Subgroups**. This figure shows exploratory stratified estimates of the effect of the PCC intervention from adjusted analyses with 95% confidence intervals on our four main outcomes of interest: (A) Time to return to Care after treatment interruption, (B) Time to Repeat Treatment Interruption after Return, (C) In Care 1-year after Return, and (D) Overall Proportion in Care 1-year after Initial Treatment Interruption. In Panels A, C, and D, positive outcomes to the right of line of null effect favor intervention in (i.e., increased return and retention); In Panel, B negative outcomes to the left of line of null effect favor intervention (i.e., decreased treatment interruptions). *Scheduled Appointment Interval either at the last visit prior to treatment interruption (Panels A and D) or at the return visit (Panels B and C). ART=antiretroviral therapy.

**Table L. Effect of PCC Intervention on Return to Care and Retention Stratified by Study Period**

|  | **Time to Return** | **Time to Repeat Treatment Interruption** | **In Care at 365 days after Treatment Interruption** | **In Care at 365 days after Return** |
| --- | --- | --- | --- | --- |
|  | **Adjusted Hazard Ratio (95% CI)** | **Adjusted Hazard Ratio (95% CI)** | **Adjusted Risk Ratio (95% CI)** | **Adjusted Risk Ratio (95% CI)** |
| Period 1 (Pre-COVID) | 1.23  (0.98, 1.53) | 0.55  (0.39, 0.78) | 1.19  (1.03, 1.36) | 1.13  (1.03, 1.23) |
| Period 2 (COVID) | 1.20  (1.03, 1.39) | 0.48  (0.33, 0.70) | 1.17  (0.92, 1.48) | 1.26  (1.03, 1.55) |
| Period 3 (Post-COVID) | 1.38  (0.74, 2.61) | 0.27  (0.19, 0.38) | Not estimable due to insufficient follow-up time | |
| Period 4 (Post-COVID) | Not estimable as all clinics in intervention during this period | | | |

**Analysis Code Description:**

*S1 File PCC Reengagement Multistate Data Setup and Analysis – Stata* includes the code for the initial data set up for multistate analysis using the *multistate* command in R. It starts with loading "PCC Reengage Multistate Dataset.dta", a long data file with all participants and event dates. This data file is available upon request. This file also includes code for the regressions for the primary analyses.

*S2 File Run PCC Reengage Multistate – R* includes the code to run the multistate analysis in R after appropriately formatting data using the initial Stata code and generating “PCC Reengage Multistate.csv”
