# Supplementary material for: Effect of a person-centered care intervention on return and sustained reengagement after treatment interruptions from HIV care in Zambia: A post-hoc analysis of a stepped-wedged cluster randomized trial
Source: PLoS Med. 2026 Sep 1;23(9):e1005052. doi: 10.1371/journal.pmed.1005052 (PMC13533374; doi:10.1371/journal.pmed.1005052)
Supplement: Supplementary file 2 [file pmed.1005052.s002.docx]

**CONSORT Extension for Stepped-Wedge Cluster Randomised Trials (SW-CRT) — Checklist**

| **Item** | **Checklist item** | **Location** |
| --- | --- | --- |
| **Title and abstract** | | |
| 1a | Identification as a SW-CRT in the title | Title |
| 1b | Structured summary including trial design, methods, results, conclusions; description of stepped wedge design | Abstract |
| **Introduction** | | |
| 2a | Scientific background, explanation of rationale, and rationale for using a stepped wedge design | Introduction, paragraphs 1–3; Methods, paragraph 1, Protocol Supplement |
| 2b | Specific objectives or hypotheses | Introduction, paragraph 3 |
| **Methods — Trial design** | | |
| 3a | Description and diagram of trial design including cluster definition, sequences, clusters per sequence, periods, step duration, data collection, control condition | Methods, paragraph 1, 4; Figure S2 |
| 3b | Important changes to methods after trial commencement, with reasons | Methods, paragraph 4 |
| **Methods — Participants** | | |
| 4a | Eligibility criteria for clusters and participants; whether determined before or after randomisation | Methods, paragraph 3, 4 |
| 4b | Settings and locations where data were collected | Methods, paragraph 1 |
| **Methods — Interventions** | | |
| 5 | Interventions for each condition with sufficient detail to allow replication | Methods, paragraph 5, Supplemental Appendix (Intervention Procedures) |
| **Methods — Outcomes** | | |
| 6a | Pre-specified primary and secondary outcome measures, including how and when assessed | Methods, paragraphs 7–9; Figure 1 |
| 6b | Any changes to trial outcomes after trial commenced, with reasons | N/A |
| **Methods — Sample size** | | |
| 7a | How sample size was determined, including allowance for clustering, time effects, and repeated measures | Table S1 |
| 7b | Interim analyses and stopping guidelines | N/A |
| **Methods — Randomisation** | | |
| 8a | Method used to generate random allocation sequence; unit of randomisation | Methods, paragraph 4 |
| 8b | Type of randomisation; details of any restriction | Methods, paragraph 4 |
| 9 | Mechanism used to implement the random allocation sequence; allocation concealment | Methods, paragraph 4 |
| 10 | Who generated the sequence, enrolled participants, and assigned to sequences | Methods, paragraph 4 |
| **Methods — Blinding** | | |
| 11a | Who was blinded after assignment and how | Methods, paragraph 4 |
| 11b | Similarity of interventions | N/A |
| **Methods — Statistical methods** | | |
| 12a | Statistical methods for primary and secondary outcomes including time effects, clustering, and repeated measures | Methods, paragraphs 9–12 |
| 12b | Methods for subgroup, sensitivity, and adjusted analyses | Methods, paragraphs 9–11 |
| **Results — Participant flow** | | |
| 13a | Numbers of clusters and participants assessed, assigned, treated, and analysed; flow diagram by sequence | Results, paragraph 1; Figure 2; Table S2 |
| 13b | Losses and exclusions for clusters and participants with reasons | Results, paragraph 1; Figure 2; Methods, paragraphs 6 and 10; Table S3, S10, S11 |
| **Results — Recruitment and baseline data** | | |
| 14a | Dates defining steps, initiation of intervention, and deviations from planned dates | Results, paragraphs 1 and 2; Figure S2; Table S2 |
| 14b | Why the trial ended or was stopped | N/A |
| 15 | Baseline demographic and clinical characteristics by treatment condition and sequence | Table 1 |
| **Results — Outcomes and estimation** | | |
| 16 | Results for each outcome by treatment condition and time period; effect size and precision | Results, paragraphs 3–7; Figures 3–4; Table 2, Tables S6, S10, S11 |
| 17a | Adjustment for time effects; secular trends | Results, paragraphs 6–7; Table 2, Tables S11 |
| 17b | Absolute and relative effect sizes for binary outcomes | Results, paragraphs 6; Table 2, Tables S11 |
| 18 | Subgroup, sensitivity, and adjusted analyses | Results, paragraph 7; Figure S8, Tables S10, S11, S12 |
| 19 | Important harms or unintended effects in each condition | N/A |
| **Discussion** | | |
| 20 | Trial limitations, including sources of potential bias and limitations of the SW-CRT design | Discussion, paragraph 5 |
| 21 | Generalisability of trial findings | Discussion, paragraph 2-4, 6 |
| 22 | Interpretation consistent with results; consideration of secular trends | Discussion, paragraphs 1-4 |
| **Other information** | | |
| 23 | Registration number and name of trial registry | Methods, paragraph 2 |
| 24 | Where the full trial protocol can be accessed | Protocol Supplement |
| 25 | Sources of funding and other support; role of funders | Funding section; Methods, Role of the funding source paragraph |
| 26 | Ethical approval and consent | Methods, paragraph 2 |

**References**

Hemming K, Taljaard M, McKenzie JE, et al. Reporting of stepped wedge cluster randomised trials: extension of the CONSORT 2010 statement with explanation and elaboration. BMJ. 2018;363:k1614. doi:10.1136/bmj.k1614

For more information please see:

https://www.consort-spirit.org/ - CONSORT
