# Supplementary material for: Effect of a person-centered care intervention on return and sustained reengagement after treatment interruptions from HIV care in Zambia: A post-hoc analysis of a stepped-wedged cluster randomized trial
Source: PLoS Med. 2026 Sep 1;23(9):e1005052. doi: 10.1371/journal.pmed.1005052 (PMC13533374; doi:10.1371/journal.pmed.1005052)
Supplement: Supplementary file 3 [file pmed.1005052.s003.docx]

Person-centred public health for HIV treatment in Zambia Main Study

(PCPH)

Protocol Version 1.5

18^th^ January 2021

Sponsor: The Bill and Melinda Gates Foundation

Table of Contents

[1.0	BACKGROUND AND INTRODUCTION	4](#_Toc2661382)

[2.0 SIGNIFICANCE	4](#_Toc2661383)

[3.0 RATIONALE	5](#_Toc2661384)

[4.0 LITERATURE REVIEW	5](#_Toc2661385)

[4.1 Build on Existing Literature	5](#_Toc2661386)

[4.2 Patient Barriers to Care	6](#_Toc2661387)

[4.3 Emerging role of health	7](#_Toc2661388)

[5.0 RESEARCH AIM	8](#_Toc2661389)

[5.1 Study Objectives	9](#_Toc2661390)

[6.0 INTERVENTION DESIGN	10](#_Toc2661391)

[7.0 STEPPED WEDGE DESIGN	11](#_Toc2661392)

[8.0 METHODOLOGY	12](#_Toc2661393)

[8.1 Study Objectives	12](#_Toc2661394)

[8.1.1 Study Design for Objective 1	12](#_Toc2661395)

[8.1.2 Study Design of Objective 2	12](#_Toc2661396)

[8.1.3 Study Design of Objective 3	12](#_Toc2661397)

[8.1.4	Study Design of Objective 4:	13](#_Toc2661398)

[8.2 Study Population for Objective 1	14](#_Toc2661399)

[8.3 Data collection for Objective 1	17](#_Toc2661400)

[8.4 Qualitative Measures	18](#_Toc2661401)

[8.5 Analytic approach for Aim 1	21](#_Toc2661402)

[8.6 Design of Objective 2 (Service Delivery and Patient Experience)	22](#_Toc2661403)

[8.7 Overview of Objective 3	26](#_Toc2661406)

[8.8 Analysis for Objective 3	28](#_Toc2661407)

[8.9 Design of Objective 4	28](#_Toc2661408)

[9.0 ETHICAL CONSIDERATIONS	30](#_Toc2661411)

[9.1 Informed Consent	32](#_Toc2661412)

[9.2 Risks to Participants	36](#_Toc2661413)

[9.3 Methods to Minimize Risks	37](#_Toc2661414)

[9.4 Anticipated Benefits to Participants	37](#_Toc2661415)

[9.5 Privacy of Individuals	38](#_Toc2661416)

[9.6 Confidentiality of Data	38](#_Toc2661417)

[9.7 Study Discontinuation	38](#_Toc2661418)

[9.8 Data Use	38](#_Toc2661419)

[10.0 TIMELINES	39](#_Toc2661420)

[11.0 BUDGET	39](#_Toc2661421)

[12.0. REFERENCES	40](#_Toc2661422)

**Roster of Investigators**

| **Name, Affiliation** | Role |
| --- | --- |
| Dr. Charles Holmes, MD, MPH, Georgetown University (GU) | GU Principal Investigator (PI) |
| Dr. Elvin Geng, MD, MPH, Washington University in St Louis | WashU PI |
| Dr. Izukanji Sikazwe, MBChB, MPH, Centre for Infectious Disease Research in Zambia (CIDRZ) | CIDRZ- PI |
| Dr. Carolyn Bolton, MBChB, MSc, University of Alabama at Birmingham (UAB) | CIDRZ, UAB -PI |
| Dr. Laura Beres, MPH, PhD, Johns Hopkins University. | Co-Investigator |
| Dr. Aaloke Mody, MD,Washington University in St Louis. | Co-Investigator |
| Dr. Mwanza Wa Mwanza, MBChB, CIDRZ | Co-Investigator |
| Dr. Nancy Padian, PhD, MPH. UCSF, CIDRZ Consultant. | Co-Investigator |
| Dr. Jake Pry, MPH, PhD~~c~~, CIDRZ, Washington University in St Louis | Co-Investigator |
| Dr. Theodora Savory, MBChB, CIDRZ | Co-Investigator |
| Dr. Anjali Sharma, ScD, CIDRZ | Co-Investigator |
| Kombatende Sikombe, MPH, CIDRZ, London School of Hygiene and Tropical Medicine (LSHTM) | Co- Investigator |

1.0 BACKGROUND AND INTRODUCTION

The scale-up of human immunodeficiency virus (HIV) treatment services has expanded rapidly in Zambia, facilitated by the evolution of the HIV response from centralized services to decentralized care and now towards differentiated service delivery models. Nevertheless, optimal effectiveness remains elusive because timely engagement in care to allow uninterrupted access to antiretroviral therapy (ART) and sustained viral suppression remain incomplete (1). Our results from a recent study in Zambia, *BetterInfo*, using a sampling-based approach to quantify mortality in the presence of high loss to follow up (LTFU), found that in the contemporary era, mortality among patients starting ART reached 7% at two years – far higher than in developed country settings – and continued to accrue at about 2% per year for up to eight years with LTFU or missing visits as the strongest predictor of mortality (2). The results from the *BetterInfo* study also demonstrated a high prevalence of clinic-based barriers to care, including rude health care worker (HCW) behaviour, short intervals between patient clinic visits, and long wait times at the facility (3). In addition, a similar study conducted in Zambia to understand reasons for patient attrition from HIV care cited amongst others clinic based barriers such as placing defaulters on intensive adherence counselling, thus imposing opportunity costs for patients and long waiting times for medical care. The study further suggested the need to improve patient provider dialogue about patient experiences(3–5). This underscored the need to better understand these clinic-based drivers of engagement and, overall, to improve the patient experience, in essence, a more patient-centred care approach. Unfortunately, little is known about how to evaluate and implement patient-centred care in the public health context of HIV care in sub-Saharan Africa (6). To address these knowledge gaps, we aim to systematically evaluate the overall patient experience and its effect on retention in care and virologic suppression via multiple novel measurement modalities in public ART clinics in Zambia. This study will provide important direction for future interventions to implement more patient-centred care in the current public health HIV infrastructure, as it will fill important knowledge gaps regarding clinic-based barriers to care.

2.0 SIGNIFICANCE

Although the evolution of health systems toward decentralization (7) and now differentiated service delivery models (1) have extended the reach of HIV services, gaps remain. In 72 clinics providing treatment to over 180,000 patients in Zambia, the Bill and Melinda Gates Foundation (BMGF)-funded BetterInfo study found that 22% of all patients were LTFU after two years (2). Furthermore, the rate of disengagement from care varied by over 10 fold across facilities – differences that were not explained by adjustment for facility size, geogreaphic location nor other characteristics, and which we believe to be due to nature of setting and services at those facilities. Interviews among a random sample of lost patients intensively traced and found in the community revealed variable barriers to care including clinic based barriers (e.g., rude health care workers, excessive facility waiting times) reported by up to 80% of patients by facility, and structural barriers (e.g., transportation, work demands) reported by up to 70% of patients by facility. In addition, we also carried out a discrete choice experiment in which HIV patients were offered a series of choices between two hypothetical facilities with different combinations of attributes and asked which they would prefer – thus quantifying the relative rank and strength of patient preferences – and potentially informing prioritization of health systems improvements. These choice experiments revealed that patients were willing to trade up to 15 hours of waiting time for “kind” health care workers and seven hours of waiting time for longer drug refill intervals. Improving the patient experience and retention represents a critical step toward sustained treatment and full epidemic control.

3.0 RATIONALE

Evidence from this study as well as throughout Africa points to the gaps in the patient experience as a crucial area for improvement (3,4,6,8,9). These data offer a simple synthesis and strategy for improvements. That is, when patients perceive encounters with the health system to be high-quality, respectful, accessible, responsive, and effective, engagement is strengthened (9). We therefore set out to make public health HIV treatment more patient centred, manage and enhance perceptions of quality, and strengthen engagement to optimize treatment. To do so, first, facilities require a scalable measurement platform to systematically capture the patient experience (e.g., satisfaction, perceived quality, communication, patient outcomes) and make this information routinely available to HCWs. Second, a strategy using insights from networking science (interplay of human behaviour, technology and information diffusion) is needed to disseminate perspectives to promote patient centeredness, shared decision making and address prevalent barriers to care. Although a patient centred public health for HIV care in Africa cannot accommodate every individual’s circumstances, redesign can better meet the needs of many. Person centred care has been shown to improve outcomes and reduce expenses in medical settings in North America for conditions ranging from diabetes to HIV (8,10–12). Other studies in Zambia and Uganda evaluated the impact of a large health initiative on quality of care and satisfaction among patients and maternal health workers (12). Because the specific barriers to patient engagement vary in intensity and nature across facilities, we believe that measurement of patient-reported experiences at each facilities level represent a crucial strategy for targeting the intensity and nature of improvement efforts. We propose introducing and adapting these practices into the public health setting in Zambia and determining other relevant practices for Zambia to redesign public health delivery.

# 4.0 LITERATURE REVIEW

## 4.1 Build on Existing Literature

*We propose to build on existing literature as well as preliminary data developed in Zambia and generated by this group.*

Our proposal is structured around multiple well-established frameworks to gain useful and practical insight into how to improve the HIV patient experience in Zambia, ultimately with the aim of improving overall patient outcomes. First of all, our project is informed by the substantial amount of data originating from sub-Saharan Africa -- including our own from Zambia -- on the barriers patients face to remain engaged in HIV care, including structural barriers (transportation, work conflicts, etc.), clinic-based barriers (rude health care workers, long wait times, etc.), and psychosocial barriers (stigma, denial, etc.) (13–16). Multiple studies have cited poor communication as a significant clinic-based barrier (12–19). Furthermore we have shown that the prevalence of these diverse barriers differ across facilities. In other words, some facilities have a preponderance of structural problems (e.g., transportation) while others have higher levels of clinic based problems or psychosocial issues among clients. We leverage these insights in developing our protocol in order to gain deeper understanding of the drivers of these barriers and their consequences for HIV care in Zambia.

Second, we believe the concept of patient-centred care represents an overarching framework to make services more penetrant, sustained and effective. Patient centeredness includes an analysis of dimensions of the patient and provider relationship, recognition that a patient’s care exists in the context of their entire life (bio psychosocial perspective), and shared responsibility and decision-making to underscore the goals of this project (20,21). The concept of patient-centred care is well-established in healthcare systems in developed countries. In North America, more patient-centred HIV care has been associated with improved patient outcomes (8,9,22,23). Currently, there is burgeoning interest in how the current public health infrastructure in sub-Saharan Africa can accommodate more patient-centred practices. Differentiated ART delivery and HIV care seek to implement elements of patient-centred care with limited scope, but little is known about how to comprehensively incorporate patient-centred practices into HIV care in Zambia and sub-Saharan Africa and the impact that this could have on patient outcomes (1,6,24–26). Patient centeredness also informs our approach to a “facility strategy” articulated in this proposal: identifying and focusing on facility-specific problems takes a step toward greater patient centeredness, and one that is appropriate for public health settings. The facility strategy includes identification of elements of the HCW experience that impede or enhance a health worker’s ability to deliver patient-centred care and engaging in efforts with HCWs to improve their experience in service of improving the patient experience.

Lastly, we consider health system responsiveness, as established by the World Health Organization (WHO), as a key element to health care quality and an intrinsic goal for health care systems underlying our measurement of the HIV clinic experience. Health system responsiveness is a system’s ability to respond to the legitimate expectations of its potential clients and relates to how individuals are treated and the environment in which they are treated (18,27,28). In this proposal, we seek to integrate our understanding of patient barriers to care, patient-centred care, and health system responsiveness in order to describe the HIV clinic experience from these perspectives and its impact on patient outcomes in Zambia. Many studies on engagement and retention in both HIV care and other chronic conditions focus on a particular intervention, such as perhaps SMS messages or conditional cash transfers. A health systems based approach, founded on patient centeredness as a guiding principle, and the epidemiological observation of heterogeneity of problems represents a different approach – one focused on the health system responsiveness rather than any particular intervention.

## 4.2 Patient Barriers to Care

There has been a significant body of literature to date on the barriers patients face in accessing HIV care, and although barriers are diverse, there is a consistent observation that facility based operational factors such as waiting times, disrespectful providers, and lack of privacy are prevalent. Previous studies from sub-Saharan Africa have described a diverse range of barriers faced by HIV-infected individuals when accessing care including structural barriers (e.g., transportation, time away from work), clinic-based barriers (e.g., wait times, rude health care workers), and psychosocial barriers (e.g., stigma). Studies from East Africa using a sampling-based approach to trace patients who were LTFU, found that though structural barriers are commonly cited (65%) among all patients LTFU, the patients who disengaged from care (as opposed to those who silently transferred to another clinic) were more likely to cite a psychosocial barrier such as stigma or treatment fatigue (76%) (29,30). This agrees well with data for Zambia from the *Better Info* study. In the *Better Info* study, disengaged patients also cited psychosocial barriers more frequently, but when asked what would need to change for them to come back to clinic, patients most frequently cited clinic-based barriers such as decreased rudeness of health care workers. Additional qualitative work from Nigeria, Tanzania, and Uganda also cited fear of scolding or reprisal from health care workers as a barrier to re-engagement to care (14). Discrete choice experiments from *Better Info* in Zambia as well as other studies in Ethiopia, Tanzania, and Mozambique further highlight the importance of health care worker attitudes towards patients and interpersonal interactions (12,17). In addition to their association with retention in care, an increased number of barriers to care has been shown to be associated with increased mortality in South Africa (31).

Understanding patient perspectives and using them to improve retention is an emerging strategy for clinical care, but has not been explored in the public health setting in Africa. Patient-centred care is a multi-faceted approach to care broadly defined as “providing care that is respectful of and responsive to individual patient preferences, needs, and values and ensuring that patient values guide all clinical decisions” (32). It is one of the six major pillars of health care quality adopted by the Institute of Medicine in the United States and has also been associated with improved patient outcomes in the United States, including in HIV care (32). In North America, patient-centeredness and patient satisfaction with the care they received have both been associated with improved outcomes in HIV care (8,9,22,23). There have been attempts to evaluate the patient experience based on the tenets of health-system responsiveness in sub-Saharan Africa, but not its association with patient outcomes and little is known about patient-centred care overall in our setting (1,6,18,24–26,33).

## 4.3 Emerging role of health

Technologies for mobile communication are increasingly accessible across the globe, with an estimated 7.7 billion mobile phones being used around the world in 2017 (34). There has been mounting interest in capitalizing upon the promise of using mobile technology to support the achievement of health priorities in sub-Saharan Africa (35) which, by the end of 2016, was home to over 420 million unique mobile subscribers (36). In Zambia, according to the 2015 ZICTA Survey Report, 33.1 percent of households have access to the national grid and 64.5% of households own a mobile phone (37).

Given the rapid uptake of mobile technologies, Mhealth, broadly defined as the usage of mobile phone technology for health-related purposes (38), has recently emerged as an important intervention with the potential to impact health service delivery and public health systems worldwide. Zambia has seen several large scale initiatives in health, including several on the topics of vulnerable children, HIV patient care, nutrition, and integrated community case management (39,40). There is a growing body of evidence to support the use of mobile technology for frontline health workers (41), including the use of mobile technology to improve quality of care of services at facilities (42).

As we take a systems approach to designing an intervention that uses SMS surveys, smartphone applications, dashboards, and feedback mechanisms, it is important to note the value of following guiding principles in design practices, in particular the Principles for Digital Development (43), the WHO era checklist (44) and Human-Centred Design.

5.0 RESEARCH AIM

Although the evolution of health systems toward decentralization and now differentiated models of care have extended the reach of HIV services, treatment outcomes are still far from uniformly successful. Even though few patients stop care permanently, lapses in care from weeks to months are still common (i.e., patients are inconsistently in care), and some settings lapses in treatment likely reaches nearly 50% after three years. In addition, even though retention in the system is higher than retention in a particular clinic, recent analyses suggest that patients who have undocumented transfers between facilities (“silently transfer”) are up to five-fold more likely to be viremic than patients at their original clinics (45). While large-scale population based surveys in Africa suggest that 85% or more of patients on treatment are suppressed, these figures do not necessarily take into account those who have been on treatment in the past but who have now lapsed, which could influence estimates considerably (46).

Furthermore, recent research work in Zambia done by this group suggests that looking nationally or even regionally hides important variation in the magnitude and types of barriers to care across facilities. We found, for example, that mortality rates, even after adjusting for CD4 levels ranged from 2% to 20%, and that lapses in retention varied from 5% to 40% by two years (2,47). When lost, patients were sought in the community and surveyed, their reasons for stopping care reflected a great deal of variability. In some facilities, transportation problems were paramount, while in others psychosocial problems (such as stigma and depression) were prevalent (and transportation problems were uncommon)(3,48,49). This across-facility variability in both the magnitude and the nature of service delivery problems leads to our scientific premise: if problems with performance are site specific, then the intensity and nature of the intervention should be targeted to the measured problems at each site.

Over the past year, we have proposed and refined a three-part intervention to determine site-level barriers to care and target interventions to those problems. We call this approach Patient Centered Care in Public Health (PCC). In short, the intervention has three interlinked components. First, we will make systematic measurements of the patient experiences (e.g. patient reported barriers to engagement, satisfaction) and outcomes (e.g. retention, viral load monitoring) at the facility using SMS, exit surveys, SmartCare data, and leveraging on already existing tracing of lost patients. These measurements will provide a “diagnosis” for each facility as to what the level of performance is as well as the prevailing problems. Second, we will train and use practice facilitators (i.e., “coaches”) to feed these data on patient experience and performance back to the facilities and guide their respond using quality improvement methodologies targeting the prevalent barriers at each facility. Finally, we will motivate the sites through an incentive schema. Each of these interventions are themselves evidence-based both in the scientific literature as well in local practice. We combined them and tailor them to the local environment through mixed methods, design workshops and key informant interviews. We hypothesize that we will magnify their effects when used in concert.

We now propose to evaluate the effect of this three-part PCC intervention through a stepped wedge cluster randomized trial design. We will approach approximately 24 to 34 facilities in Lusaka Province supported by CIDRZ for participation. We will roll out the intervention in four groups every six months over 18 months and follow facilities for a total of 24 months. We will increase the probability of randomization into the first and last waves to achieve adequate power for longer term retention outcomes. We will evaluate the implementation, effectiveness and cost effectiveness of the intervention on patient experience and clinical outcome measures. Our specific objectives are fourfold as below.

5.1 Study Objectives

Our four main study objectives are:

1. To evaluate the implementation of the PCC intervention in routine care setting in Zambia
2. To evaluate the effect of the PCC intervention on service delivery and patient experience
3. To evaluate the effect of the PCC intervention on retention and viral suppression
4. To evaluate the cost and cost effectiveness of the PCC intervention.

These are further explained below.

**Objective 1: Evaluate the implementation of the PCC intervention in routine care setting in Zambia.**

Even though our team has undertaken a participatory process with health care workers to develop the intervention, understanding the extent that a complex intervention is delivered is fundamental to understanding the effects of the intervention being tested (whether positive or not). We will document the occurrence of each of the three components of the PCC intervention. These will be the extent to which we measure patient experience and outcomes as intended, the success of training, data sharing, and coaching (as measured by the adequate encounters between the facilities and practice facilitators) and the process of implementing an incentive, including mixed methods approaches to understand how these were perceived by the patients and health care workers. To the extent that the implementation of these factors could be effected by above site systems failures (e.g., health care workforce vacancies, facility power supply), we will document and track such events.

With first diagnosed case of COVID-19 in Zambia on the 18^th^ of March 2020, the context of the implementation of the PCC intervention has changed dramatically. As the COVID-19 pandemic progresses globally and in Zambia, the situation in which HIV care is delivered will be dynamic. Initial responses included the Ministry of Health issuing a directive on 24^th^ March 2020 to dispense a 6-month antiretroviral therapy (ART) supply to all patients on HIV treatment, and all sites began a process of calling patients into the clinic, regardless of the timing of their last visit. In accordance with guidance from CDC and PEPFAR, community-based activities such as testing campaigns have been suspended; and resources redirected to surveillance and testing for COVID-19. Health facilities have implemented measures including screening of patients of symptoms upon arrival, triage of suspected COVID-19-positive patients, maintaining a distance of 1 metre or more between patients who are waiting to be attended to and installing additional handwashing stations. Just as the context has changed, the experience of patients and providers has changed. To understand HIV care delivery – critical and life-saving services that must continue under any circumstances – we will assess the patient and provider experiences under these conditions, leveraging the study platforms established for prior measurements.

**Objective 2: Evaluate the effect of the PCC intervention on service delivery and patient experience.**

We will seek to evaluate the effect of the PCC intervention on the patient experience as measured by multiple modalities at each of the facilities. We will carry out assessments of the patient experience using four modalities through the study and will evaluate each one as distinct outcomes as well as a composite score: exit interviews among a systematic sample of patients leaving the facilities, “trained” patients exposed to exit interviews before their visit (Quality Improvement Patient Surveys QIPS), a structured documentation of the patient-provider interactions in the form of the Roter Interactive Analysis System (RIAS), and patient reported barriers to care from tracing of lost to follow up patients. At each of the facilities, we will carry out these assessments continuously, and example aggregate scores during intervention and non-intervention periods. We will document above site unanticipated events that impact on service delivery and patient experience.

**Objective 3: Evaluate the effect of the PCC intervention on retention and viral suppression.**

Ultimately, we seek to assess the effect of the targeted facility intervention on patient behaviors and clinical outcomes. We will use waves one and four of the stepped wedge design to evaluate the impact on retention and viral suppression. Our primary outcome will be retention, as measured by first episode of missing a clinic visit or drug pick up by 14 or more days. Secondary outcomes will be medication possession ratio, the “time in care” metric (defined as the fraction of time in care that the patient is late), time event analyses with longer intervals (30, 60 and 90 days) and viral load. The research team will support the facility to carryout routine monitoring and documentation of those results through existing data review meetings to strengthen facility performance as well as obtain crucial measurements to document intervention effects. In particular, as monitoring and evaluation systems have further expanded to capture engagement in community programs such as uptake of differentiated service delivery models, and the study will support existing efforts at the facilities to document clinical encounters, including those that happen outside of the facility.

**Objective 4: Evaluate the cost and cost effectiveness of the PCC intervention.**

Given the limited global resource envelope for interventions, policy makers need to know not only the effects of any public health innovation, but also their cost and cost effectiveness. We will review administrative data, carryout observation and time and motion studies to document the costs of the PCC intervention. Cost-effectiveness will be evaluated from both a healthcare and modified societal (i.e., healthcare plus patient) perspective. We will estimate the incremental cost and incremental effectiveness of the PCC intervention enabling us to calculate the incremental cost-effectiveness ratio (ICER) for this intervention.

# 6.0 INTERVENTION DESIGN

The PCC intervention is a health systems intervention that seeks to improve the patient-centredness of HIV care to improve the patient experience and, ultimately, clinical and care outcomes by: 1) systematically assessing the patient experience (e.g., satisfaction, barriers to care), facility approaches to patient care (e.g., use of differentiated service delivery models, monitoring viral load, how late and lost patients are welcomed back to care), and key patient outcomes (e.g., retention); 2) providing HCWs training in patient-centred principles and skills and mentoring to improve patient-centred practice using the collected data to target action; and 3) offering incentives to motivate facilities above and beyond their inate desire to help improve patient lives.

Part 1 of the intervention is to add continuous measurement of the patient experience and outcomes using four new modalities (exit interviews, QIPS, SMS surveys, and structured assessments among patients who are lost to follow-up) along with existing data to be drawn from SmartCare and other routine data sources to highlight current standard measures of performance (e.g., retention, completeness of viral load monitoring, and possibly viral suppression and mortality). Other standard measures will include any above-facility events or trends that could influence the implementation of the PCC interventions or it’s effects on service delivery or patient outcomes. Such events include facility drug supplies and staffing levels that could affect service delivery as well as unanticipated events such as political instability or severe climate events as well as policy changes. These measurements may provide context for the observed effects.

Part 2 is summarizing the data and delivering them to facilities in appropriate fora, such as at quarterly staff meetings, in the context of quality improvement coaching and training. All data will be summarized and delivered to facilities as part of a public health patient experience management system (PPEMS) that will aim bring the patient experience into routine program data. The coaching begins with a structured curriculum focused on patient-centeredness principles and skills. Subsequent coaching visits to facilities by trained practice facilitators will strengthen existing quality improvement practices at the facility to enable facilities to effectively respond to the stream of data we will provide about the patients experience as well as facility performance using metric such as the patient exit interviews (from Part 1) as well as metrics of performance drawn from routine data (such as the completeness of viral load monitoring). We will act through bolstering existing quality improvement mechanisms (e.g., improvement committees, root cause analyses, ‘Plan, Do, Study, Act’ (PDSA) cycles).

Part 3 is to enhance the motivation of the clinic to act on these findings, and improve them, through showing facilities their performance as compared to other facilities (in order to motivate lower performing facilities) and offering rewards for good performance. Empirical data suggests that health care workers compare their performance to those of peers and we leverage this effect to create a gentle reputational incentive.

Overall, this approach seeks to create the ability to match targeted improvement activities on local, facility-level drivers of the most prevalent barriers to care at each facility. Targeting improvement activities can enhance effectiveness by intensifying focus on the most important problems at a specific facility, as well as potentially improve efficiency through minimizing less relevant activities at a specific site. In addition, “pay for performance” schemas are supported by widespread empirical evidence as well as theory that organizational change has a cost to health care workers, in that they must reinvent routines and processes, and that an small external incentive can help.

Overall, the intervention is facility-based and both health care worker and patient-focused. The design acknowledges that different facilities and teams may have different preferences and requirements which, if identified and addressed, may improve intervention success. The study will employ human centred design (HCD) methodology to ensure appropriate participant engagement and adaptation of interventions throughout implementation. The coaches will utilise quality improvement and methods such as process mapping, prototyping, and other activities throughout the curriculum-based training, information sharing and coaching activities to ensure high quality implementation of the intervention.

7.0 STEPPED WEDGE DESIGN

Stepped-wedge cluster randomized designs are used when equipoise is not met (because the preponderance of evidence favours the intervention), but when there is also a desire for rigorous inference about the magnitude of the effect (50). We plan on evaluating approximately 34 facilities mainly in Lusaka Province which are between 500 and 10,000 patients currently on ART. We will roll out the intervention in four groups, at time zero, 6, 12 and 18 months, and follow all sites for a total of 24 months. We employ an open-cohort design where new patients can enter observation time at any time during the trial and be measured for outcomes at multiple times. Some measurements which are made repeatedly overtime, and in these cases, it is possible that a single patient if surveyed more than once can contribute to the patient experience or service delivery outcomes both before and after cross over. For other outcomes such as retention and viral load which represent cumulative effects of longitudinal exposure to the intervention, we will censor at crossover. Because we seek to detect both the impact of the intervention on the patient experience (which can occur over shorter term) as well as retention and viral load (over a longer term), we will weight the steps unequally to achieve adequate power in both types of outcomes. Specifically we seek to randomize approximately 75% of facilities to the first and last waves and 25% to waves two and three.

8.0 METHODOLOGY

8.1 Study Objectives

8.1.1 Study Design for Objective 1

Objective 1 is a mixed method evaluation of the implementation of the three components of the intervention (i.e., implementation outcomes). As such, it seeks descriptive epidemiological and qualitiatve findings. We will capture the occurance of implementation activities (e.g., training, coaching visits, incentive schema) through structured and semistructure electronic forms. This aim also utilizes a combination of in-depth qualitative interviews with patients, focus group discussions (FGDs) with HCWs, records kept by coaches and data collection staff members, and direct observations. The first component includes measurements of patient experience and outcomes which will be used both to inform the nature of coaching and improvement processes at each facility, and what we will use to understand the effects of the intervention. Because we are measuring implementation of the intervention, this will take place at the intervention facilities only.

The ability to understand the extent to which procedures to measure experience and outcomes, as well as interventions to influence them, were carried out will be critical to understanding their effects (or lack thereof). We will capture these quantitatively with “cascade” analyses which describe the implementation process as a series of steps and then quantify the extent to which those steps were met.

8.1.2 Study Design of Objective 2

In objective two, we examine the effect of the PCC intervention on patient experience outcomes and service delivery outcomes using a stepped wedge, cluster randomized trial design. As described above, we plan to roll out the intervention in approximately 34 healthcare facilities. These facilities will be stratified by size and geography to ensure balance of facilities types and locations over time and across intervention conditions. Within strata, facilities will be randomly allocated to one of four waves. Each six months, the intervention will be introduced in one of the four waves at time zero, 6 , 12 and 18 months. We plan a total of 24 months of follow-up time. The outcomes for this objective are patient experience (e.g., exit interviews) and metrics of service delivery (e.g., completeness of viral load monitoring and trained patient exit interviews). Measurements will be made continuously during the study period. We will estimate the effect using “vertical” approach in which period specific estimates are combined without explicit model specification. The outcomes will be differences across these patient experience and service delivery metrics.

8.1.3 Study Design of Objective 3

Objective three examines the effect of the PCC intervention on clinical *care* and clinical outcomes of retention and viral suppression using the same stepped-wedge cluster randomized design. Because retention and viral load are outcomes that may take longer to influence, we design the stepped wedge to weight the first and last steps (which will receive ~ 75% of the facilities), and therefore increase the periods of time where the potential effects on retention and viral suppression can take place. In the design, we will have 18 months of contemporaneous observation time in which some facilities are experiencing the intervention and some the control. We will seek to measure the effect of the intervention one year after patients are exposed to it, which allows a cohort of all patients getting HIV care or treatment at the selected facilities who have encounters over the first six months of observation in waves one and four to accrue at least one year of observation time. Retention will be measured using routine clinic data extracted for the cohort of patients, and our primary outcome will be missing a clinic visit by 14 or more days using stratified Kaplan Meier estimates. We will explore alternative metrics of retention as secondary outcomes including time in care (i.e., the fraction of follow up time compliant with the appointment schedule, medication possession ratio and visit adherence). Given the importance of documentation of clinical and pharmacy appointments and visits, including in community based care, we will support the sites to ensure high performance of normal systems to document and track these data including tracing related outcomes from LTFU patients. We will also assess viral load one year after exposure to the intervention in a cohort of patients with an encounter at the facility between time zero and 6 months (to be measured between 12 and 18 months). A baseline viral load will also be collected in the cohort between time zero and 6 months from intervention and control sites. Our cohort of viral load participants will include new ART , already on ART ,late/LTFU. Viral load monitoring is now standard, and we will assist facilities (through their normal patient monitoring practices) to complete MOH required monitoring schedules. In addition, we will leverage on existing patient tracing at the facility to assess mortality. We will examine differences in the proportion virally suppressed (at a cut off of 400 copies /ml) using a simple t-test.

8.1.4 **Study Design of Objective 4:**

Objective four evaluates the cost-effectiveness of the PCC intervention, from both a health systems and a patient perspective. While much of the implementation costs will occur at the central level, many costs of delivery, quality assurance/improvement, and changes in activities are likely to occur at the clinic level. Thus, while much of the costing of PCC implementation can be accomplished through key informant interviews, much of the evaluation of the other components may require observation, interview, or documentation at the clinic level. Our costing activities are designed to comprehensively assess costs that are associated with implementation of the PCC intervention, thereby enabling us to estimate costs of similar programs that might be implemented at different scales, using some (but not all) of the different components, and/or in different epidemiological/economic contexts. We will collect costs in three different manners: (a) one-time, centrally, among PCC staff; (b) one-time, at each of the 34 clinics; and (c) once per wave of the stepped wedge trial, in each of between four to eight clinics that will be purposively sampled to provide a range of urban/rural status, healthcare system level, staffing ratio (e.g., number of vacant positions), geography, and patient volume.

Overall schema of the objectives and cascade of outcomes

8.2 Study Population for Objective 1

Since objective 1 is to understand the extent to which each of the intervention was implemented, the study population for Objective 1 depends on each of three intervention components and will be carried out at each of the sites implementing the intervention. Because most of the measurements included in the PPEMS are a part of the intervention as well as the outcome (that is to say, the exit interview at a given time will be used to inform the intervention as well as at a later time represent the effects of the intervention), we include an understanding of their implementation in this objective, but it should be clear that we will measure implementation of the relevant parts of the PPEMS in the non-intervention settings to ensure the completeness of outcome ascertainment.

*Table 1*: Schema showing measurement schedule for Objective 1

|  | Period 1 | 2 | 3 | 4 |
| --- | --- | --- | --- | --- |
| Wave 4 | PPEMS | PPEMS | PPEMS | PPEMS + Training coaching + incentives |
| Wave 3 | PPEMS | PPEMS | PPEMS + Training coaching + incentives | PPEMS + Training coaching + incentives |
| Wave 2 | PPEMS | PPEMS + Training coaching + incentives | PPEMS + Training coaching + incentives | PPEMS + Training coaching + incentives |
| Wave 1 | PPEMS + Training coaching + incentives | PPEMS + Training coaching + incentives | PPEMS + Training coaching + incentives | PPEMS + Training coaching + incentives |

*8.2.1 SMS/USSD-Based Surveys (Population for Objective 1)*

The target population (those we seek to sample from) for SMS/USSD-based patient experience survey are patients currently in care, aged 18 years or older, and have access to a mobile device (i.e. owned or shared). We will seek to enrol a convenience sample of about 250-300 eligible participants (depending on the overall clinic size) to receive SMS/USSD-based surveys at each facility during each six-month period during the intervention. During clinic visits, patients will be approached by study staff using a recruitment script to determine their eligibility and voluntary willingness to participate. If they agree to participate they will have their contact information and ART number collected, and thereby be enumerated for objective 1. Patients who were enrolled in the prior 6 months and completed the SMS/USSD-based survey will maintain enrolment for the remainder of the study period. Based on pilot data survey response rate of 40% completion, this sample will allow us to collect 75-100 new complete surveys per facility per measurement period (each six months) and estimate the patient experience with a precision of approximately +/-10%. In essence the SMS population will represent a nested sub-cohort within the larger clinic population in that they will be queried more than once.

*8.2.2 Exit Survey (Population for Objective 1)*

The exit interview will aim to capture the patient experience among patients currently in care who are 18 years or older. Among patients leaving the facility after attending clinic that day, we will take a systematic (every kth) sample of patients to assess their experience in clinic that day, where k depends on the facility volume. Patients will be approached by study staff using a recruitment script to determine their eligibility and voluntary willingness to participate. If they agree, we will collect their ART number, and thereby be enumerated for the exit survey. We seek to collect 75-100 complete surveys per six months and estimate the patient experience with a precision of approximately +/-10%. Many patients will be given the opportunity to participate in both the SMS and exit surveys.

For both the SMS Surveys and Exit Surveys we seek a waiver of consent. The questions posed in the SMS and exit surveys involve minimal risk to the participants and are designed to not reveal anything about individual HIV care. They are similar to consumer satisfaction surveys used across various industries. They are also similar to data recorded during programmatic standard of care tracing of late or lost patients that are connected to their electronic medical record data. Participants are free to not respond to any questions in the exit survey or to not respond to the SMS, even if they do agree to voluntarily participate. As the surveys are short (3-13 questions) and conducted after the patient has sought care, the greatest time burden on the participant would be a written consent process. By relieving this time burden in a minimal risk environment, the study will likely permit more patients to share their care experiences, which has been shown to be desirable in both formative and pilot data collection.

*8.2.3 BetterInfo Lite (Population for Objective 1)*

The target population for *Better Info* Lite procedures are patients who are currently LTFU according to facility practices. Of note, the tracing of lost to follow-up patients and recording of their vital status and barriers to care is now a priority for all sites from donors and the Zambian MOH, and is therefore considered standard of care. We will support this existing tracing being conducted routinely at study sites and this will include all data on patient reported reasons for engagement or disengagement. Our support to the facility will include sharing our experience on the BetterInfo study and ensuring facility staff are well trained on ensuring tracing outcomes are met. We will also support facilities if needed to identify patients that are lost to follow up using the Better Info algorithm (REF. No 004-06-14) as well as to revise facility specific retention estimates based on facility tracing outcomes. If further intensive tracing is required or desired by the facility to obtain more accurate outcome estimates for program reporting (again this is outside of the research and routinely mandated clinical reporting), among those LTFU patients, we will assist with selecting of a random sample currently LTFU using existing standards (REF. No 004-06-14).

*8.2.5 Training Curriculum and Coaching (Population for Objective 1)*

The target population for the training curriculum and coaching component of our intervention are all health care workers at our study facilities. This includes medical officers, clinical officers, nursing staff, lay health workers/counsellors, pharmacists, data managers, and others. We anticipate approximately an average of 20 health care workers across these widely varying clinics, for a total of 800 health care workers targeted for training. Assessment will be made both quantitatively (e.g., people trained, the frequency of coaching visits to the target facilities, health care worker experience survey) as reported in Coaching Logs, as well as qualitatively (e.g., how did the health care workers at our study facilities feel about the coaching, how did the study coaches feel about the coaching, what did it entail, what are patient perceptions of the intervention outcomes.) as understood through interviews with patients, focus group discussions with health care workers, what are facility vital signs that affect operations and observations at the facilities. Finally, we will assess the implementation of the incentive schema during intervention periods as understood through coaching logs or other administrative records documenting rewards given and facility performance, and FGDs with health care workers. At a high level, we will assess whether all facilities participated in the incentive schema, but will also employ mixed quantitative and qualitative methods to understand how health care workers perceived the incentive program.

*Table 2: Sample size by Activity (Population for Objective 1)*

| **Approach** | **Depth** | **Reach** | **Purpose/Use** | **Sample Size** | **Implementation metrics** |
| --- | --- | --- | --- | --- | --- |
| SMS survey | + | +++++ | SMS will be critical due to speed and reach. In addition, it will allow for potential capture of care experiences that occur in community that are not captured by exit surveys in facility based models of care | Up to average 200 measurements in each of approximately 34 facilities in each of the four study periods (200 * 34 * 4) for total maximum of 27,200 measures | Proportion of patients who agree to, receive, initiate and complete survey |
| Exit survey | ++ | +++ | Exit survey will provide the opportunity to reach a larger number of patients immediately after their contact with the health system when recall bias is minimized. | Up to average of approximately 75-100 measurements in each of 34 facilities in each of the four study periods (100 * 34 * 4) for total maximum of approximately 13,600 measures | Proportion of patients approached who agree to do, and complete survey |
| Enhanced Tracing (Better Info Lite) | ++ | +++ | Leveraging on already existing enhanced tracing and surveying patients who are LTFU will be essential to capturing the patient experience of those who have disengaged from care. | As per routine facility tracing guidelines | The fraction of patients the facility identify for tracing that are attempted  The fraction contacted and vital status updated  The fraction with adequate documentation of reasons who are contacted in person |

## 8.3 Data collection for Objective 1

*8.3.1 SMS/USSD-Based Surveys. (Data Collection for Objective 1)*

After enrolling for SMS/USSD-based surveys, participants will be automatically sent SMS/USSD-based surveys every 3 months. Participants will receive automated disbursements of varying amouts of airtime (zero Kwacha to five Kwacha) incentive for survey completion allowing us to understand strategies to optimize response rates. Participants will also receive up to 3 reminders if survey not initially completed. To measure implementation of SMS/USSD-based surveys, we will collect data on 1) the number of patients who are approached, eligible, and agree to participate; 2) the number of surveys successfully sent, initiated, completed; 3) time to response and the number of reminders sent, and 4) socio demographics among those who agree to participate.

*8.3.2 Exit Survey (Data Collection for Objective 1)*

Among patients who agree to participate, lay health care worker will administer the tablet-based survey in a private area, guiding individuals through each question and the response options. The instrument itself is described with greater detail in objective 2, but solicits their experience at the visit. To measure implementation of exit surveys, we will collect data on 1) the number of patients who are approached, agree to participate, and successfully complete the survey; 2) the time for survey completion; and 3) sociodemographic among those who agree participate.

*8.3.3 BetterInfo Lite (Data Collection for Objective 1)*

Tracing patients who are late to appointments or LTFU is now standard practice in ART clinics. We will use the Better Info protocol (REF. No 004-06-14) to enhance pre-existing tracing procedures in order to capture the experience (e.g., satisfaction, perceived quality, empathy, communication, patient outcomes) of LTFU patients using the LTFU questionnaire. We will support facilities in recording total LTFU by facility and tabulate the proportion of those randomly selected that are successfully traced (paper, phone and/or field), as well as how many in whom we obtain a completed loss to follow up assessment form. Data will be collected using a tablet-based open data kit tool in accordance with Better Info. Because we are leveraging on routine tracing activities in the facility, data may be available in paper forms too. For patients who are sought and reported dead, we will record date of death, approximate if necessary, as well as the cause of death in six simple, mutually exclusive and exhaustive categories: accident, homicide, suicide, child-birth, illness, and other. Patients will be classified as in-care, silent transfer, or out of care. These data will provide information that will be used to provide revised retention rates for facilities as per Better Info protocol (REF. No 004-06-14) (2,47).

*8.3.4 Measuring Health Care Worker Experience*

We will use the health care worker (HCW) survey questions developed and validated in Kenya among health care workers in hospitals (51). The aim of this survey will be to measure the experience of health care workers in relation to motivation, job satisfaction, stress management, and their work environment. We will seek to sample up to 30% of health care workers per health facility (up to 50 health care workers per facility) to measure the experience of health providers. We request to waive the requirement to obtain and document written informed consent from prospective participants for this survey. These participants are Health Care workers who have either been trained or mentored by the PCPH study to provide patient centred care . Consenting HCWs who are already farmiliar with the study would increase time burden. In addition, participating in the Health Care Worker survey poses minimal or no risks to the participants as most questions seeks to measure their satisfaction, motivation and experience with the PCPH study.

*8.3.5 COVID-19 Measures*

A) Working with a cohort of patients enrolled in the USSD/SMS survey, we will continue ongoing assessments of the patient experience modifying questions to reflect the new ART distribution guidelines of dispensing a 6-month ART supply. We will send out the survey one time to all enrolled participants using the same incentives and a reminder message after one day if there is no reply. Subsequently, we will send the survey 8 days following any regularly scheduled standard USSD/SMS survey to understand their experience around COVID-19 prevention measures and ART distribution going forward.

B) We will enroll up to 30 participants from the USSD/SMS cohort in telephone-administered formative, semi-structured interviews to understand the HIV patient experience and patient-centeredness in the context of COVID-19.

Using open-ended questions, we will seek to understand key patient priorities and the meaning of patient-centredness in the COVID-19 context. Participants will be purposively sampled to achieve balanced enrolment of gender and across health facilities. Study staff members trained in qualitative interviewing and telephone-based data collection will: 1) call the potential participant using their phone number from the USSD/SMS cohort, 2) confirm their identity without speaking about HIV or other topics that may be sensitive to who answers the call, 3) once confirming the potential of the individual, describe the interview opportunity and complete a process of voluntary verbal consent in the participants preferred language 4) only after voluntary consent is granted will the interview proceed. The interviews will be recorded and transcribed or, if the participant refuses to be recorded, interviews will be documented using notes taken by the interviewer. Participants will be offered a K100 reimbursement for their time spent in this interview.

C) We will enroll up to the entire USSD/SMS cohort in a telephone-administered survey covering knowledge about and impact of COVID-19. The survey will be administered monthly. Study staff members trained in telephone-based data collection will 1) call the potential participant using their phone number from the USSD/SMS cohort, 2) confirm their identity without speaking about HIV or other topics that may be sensitive to who answers the call, 3) once confirming the potential of the individual, describe the interview opportunity and complete a process of voluntary verbal consent, 4) only after voluntary consent is granted will the survey proceed. Participants may stop or decline subsequent participation at any time. All participants will be given the national phone number 909 that they can call for additional information on COVID-19.

To understand the health care worker experience, which is critical to the patient experience, we will administer the following:

D) We will offer telephone-based data collection for all health care workers currently enrolled in the in-person survey as listed in 8.3.4, to accommodate social distancing and changes in health care worker schedules at the health facility.

E) COVID-19 has affected health care worker well-being worldwide. We will offer a self-administered paper-based mental health assessment, the Patient Health Questionnaire-9 , which has previously been used successfully in Zambia (52), at all 24 study facilities once every 2 months. Participants who voluntarily self-administer the anonymous survey will drop the completed survey to the study Research Assistant at the facility. Participants may also choose the option to have a trained interviewer administer it over the phone. The questions posed in this health care worker survey involve minimal risk to the participants and are designed not to collect and reveal personal identifiers. We therefore request a waiver of consent for this survey, similar to the waiver granted for the PCC health care worker survey pre COVID-19 in section 8.3.4. The survey will include a phone number that participants can call to access mental health support if needed.

F) We will conduct a survey of personal protective equipment (PPE) access with a sample of up to 20 health care workers from each of the 24 study health facilities. We will administer the survey every 2 weeks to support monitoring of PPE through the course of the COVID-19 response. We will establish a sampling frame by compiling all health care worker telephone numbers from interactions to date at the facilities. Purposively sampling to enroll participants from each cadre, including managerial-level, professional and lay health care workers, study staff trained in telephone based interviewing will: 1) call the health care worker, 2) confirm their identity as the health care worker known to the study, 3) describe the survey opportunity and complete a process of voluntary verbal informed consent, 4) only after voluntary consent is granted will the survey proceed. Participants may stop or decline subsequent participation at any time. The short survey may also be available in paper-format at the facility to support optimal administration given the likely changing circumstances presented by COVID-19.

G) Conduct a survey administered over the telephone to a sample of up to 20 health care workers in each of the 24 study health facilities. Using the same sampling frame and consent process as above, this survey will include a monthly assessment of HIV service availability and an initial semi-structured interview in the first phone call to assess health care worker COVID-19 experiences and perspectives on HIV care delivery in the context of COVID-19. The semi-structured interview will be analysed to identify key health care worker priorities, concerns and ongoing HIV service availability.

## 8.4 Qualitative Measures

Qualitative activities will be conducted in order to understand the intervention. The main activities will include data collection using coaching logs, Focus Group Discussions (FGDs) with health care workers, In-depth Interviews (IDIs) with patients,study coaches and observations.

*8.4.1 Coaching/Mentorship Logs&Qualitative interviews with coaches*

The process and feedback of the coaching or mentoring session between the coach/mentor and health care workers will be documented in a coaching/mentorship log by the coach/mentor. This log will capture details like facility name where the mentor interaction took place, date of mentor interaction, cadre of health care worker who participated in the mentor interaction, concerns raised by the health care workers, key reflections from the mentor interaction and any other important details from the health facility relevant to the implementation of PCC (e.g. facility vitals signs on staffing, drug stock outs).

The coach/mentor will complete the coaching/mentorship log after every health facility interaction. The summarised data from coaching/mentoring logs will be presented to the study leadership team during study meetings. This data will be used to refine the coaching/mentoring engagement, influence future coaching in other health facilities, and provide implementation outcome data such as number of facility visits and activities for costing analysis.In addition, as part of study implementation monitoring, we will conduct in depth interviews with the study coaches on a monthly basis to understand their coaching experience. We therefore request for a waiver of consent for our study coaches.

*8.4.2 Focus Group Discussions (FGDs) with HCWs*

In order to understand healthcare workers’ experience with the training and coaching/mentorship on patient-centred care, we will conduct at least 1 - 2 FGDs per facility with healthcare workers drawn from the two highest and two lowest performing health facilities, or facilities selected based on other variation that we determine may be associated with the outcomes of interest such as typologies of coaching activities or facility level (rural, urban, hospital, health post). We will also conduct at least 1 FGD in up to 8 facilities to understand the perceived barriers and facilitators to integrating Viral Load monitoring into daily clinical practice from the healthcare worker perspective. This may provide new insights into the strategies that can be used to improve uptake of VL in Zambia. These FGDs will also help our understanding on the impact of Covid 19 on routine Viral Load monitoring. We will use data such as retention and viral load suppression from electronic medical records to determine health facility performance. We will use convenience sampling to identify healthcare workers who participated in the intervention, seeking variation across cadres represented in the facility.

HCWs will be recruited by the study qualitative staff who will visit the overall in-charge of the facility to explain the purpose of the FGDs and make appointments. The study qualitative staff will consult with the Overall In-Charge with regards the most appropriate time and location to recruit and conduct the FGDs. The Overall-In-Charge will inform and remind the HCWs about the scheduled meeting. At the meeting with HCWs, the purpose of the FGD will be mentioned and it will be emphasised that participation in the FGD is purely voluntary and that their participation or non-participation will not affect their job at the health facility in any way. HCWs will also be informed that should they be willing to participate in the FGD, it will last for 60 – 120 minutes, and the FGD will be audio-recorded with their permission. HCWs will be encouraged to ask clarifying questions before and after the FGD.

The FGDs with healthcare workers may take place at the beginning, mid-way and end of each wave in the intervention facilities. We may also collect data from control sites to explain possible differences arising from the PCC intervention. We will use similar procedures as intervention sites to recruit HCWs in the control sites. Specifically, we will conduct at least 1 - 2 FGDs per facility with healthcare workers drawn from the two highest and two lowest performing health facilities, or facilities selected based on other variation that we may determine. We will use data such as retention and viral load suppression data from electronic medical records to determine health facility performance. Eligibility includes being a health care worker at a study site who gives voluntary consent to participate.

Participants will be given K100 for travel reimbursement. With voluntary informed consent, interviews will be audio recorded and/or the facilitator(s) will take written notes. After the FGD, facilitators will write field notes to record their observations of the data collection experience and important themes that emerged. These field notes will be used during analysis.

*8.4.3 IDIs with Patients*

We will conduct in-depth interviews (~60-120 minutes) in approximately 40 patients to capture their experience of the intervention. Participants will be recruited when they are waiting for their clinical consultations or drug pick up at the intervention health facilities. The study Community Liaison Officers (CLOs) will work in collaboration with the qualitative staff to recruit participants in the study. We will recruit patients through convenience sampling from patients at sampled sites on data collection days. We will also recruit participants based on patient characteristics such as gender and age. Participants will be interviewed using a semi-structured interview guide. Participants will be given K100 for travel reimbursement. With voluntary informed consent, interviews will be audio recorded and/or the interviewer will take written notes. After the interview, interviewers will write field notes to record their observations of important themes that emerged. These field notes will be used during analysis. Eligibility includes being an adult (18 years or older) patient at a study site who gives voluntary consent to participate.

*8.4.4 Direct Observations*

Direction observations of health centre operations, formalized into research memos, will take place in the intervention health facilities at the beginning, mid-way and end of each wave. One to two-day direct observation exercises at up to 20 study facilities will be carried out in public areas of the facility such as waiting areas, registry, vital measurement stations, ART, pharmacy, and laboratory departments. Additionally, we will conduct observations in ART screening rooms and ART counselling rooms. Data collected during observations will contribute to measuring the patient experience by supplementing efforts of measuring welcome-back (QIP Exit clients) and patient-provider interaction (RIAS).

*8.4.5 Data Collection for Direct Observations*

Observations will be conducted over approximately two days, incorporating time spent in each of the departments (e.g. ART, pharmacy, laboratory department, screening rooms) and will be conducted by trained research staff, who will offer an objective documentation outside of the day to day documentation done by the coaches. Accompanied by the health centre in-charge, the trained observer(s) will introduce themselves to all staff in a general round of introductions at the beginning of the observation-period, and subsequently sit in various locations in each department, making shorthand notes related to their observations of health centre operations, healthcare worker interactions (verbal and non-verbal), patient-provider interactions and informal conversations. Notes will be structured under general thematic headings such as: operational features; patient-provider relations; environment / context. We will look for outcomes of interest to the study, including a kind welcome of patients, use of viral load monitoring, opportunities to utilise differentiated service delivery models, use of good communication skills, and other objectives identified by the coaches as important for that facility. Where possible, notes will be transcribed daily into an electronic log for later analysis.

*8.4.6 Procedures for Direct Observations*

Direct observations will involve a trained study team member as a passive observer in public observable zones within the clinic, such as waiting areas, departmental registry rooms and vitals measurement stations. Permission to be based in these areas will be established in the first instance with the overall-in charge at the commencement of study activities in the clinic. Based on a list of themes outlined in a semi-structured observation tool, the trained observer will sit or stand in an unobtrusive location to observe patient flow, one-on-one and group interactions and other elements of day-to-day operations. Each ‘block’ of observation will last 1-2 hours. As far as possible, the observer will not participate in conversations or activities although where necessary may answer direct questions. Short hand notes will be recorded on in summary around the following themes: (general environment and work flow; provider service behaviours; communication patterns; patient or provider group behaviours) and written up in full at the end of the day or as soon as possible thereafter.

A second category of observations will take place in the ART and OPD screening rooms and counselling rooms. For these observations, which take place in a private setting, we will seek formal written consent from the health worker involved prior to commencing observations. Since the focus of the observation relates to the manner in which services are provided such as communication style and types of services being delivered we are seeking a waiver of formal consent for patients. All patients will nonetheless be informed of the observers’ presence and have the option to request the observer leave. In such a case, the observer will exit the screening room for the duration of that screening event and then re-enter prior to the next patient. No personal or otherwise identifying information will be collected about either the patients or providers. Observations will be conducted once or twice in screening rooms of the OPD and ART and counselling rooms, in 1-2 hours blocks. During this time the trained observer will adopt a completely passive role and (following consent procedures) will not participate or interact with either the patient or provider. Short hand notes based on the following themes (screening room environment; services provided; patient & provider communication styles; screening outcomes) will be recorded on a summary memo and written up in full at the end of the day or as soon as possible thereafter.

8.5 Analytic approach for Aim 1

Previous preparatory work has been used to validate the approaches and in this objective, analysis will see to quantify the implementation outcomes (see table below).

8.5.1 SMS/USSD-based Surveys (Analysis of implementation in Objective 1)

We will describe the acceptance to participate in the SMS/USSD-based survey as a proportion of those approached that enrol as well as survey completion rate among those enrolled. In addition, we will analyse response by sociodemographic characteristics, message method (short message service and unstructured supplementary service data), and mobile network operator (of which there are currently three in Zambia) using univariate and multivariate regression models, using log link functions, and random effects to account for clinic correlations. We will estimate the precision of the SMS/USSD-based survey tool as a function of total complete responses and clinic population. Time to survey response and the role of reminders on survey completion rate will also be evaluated using time to event approaches such as the Kaplan Meier method or Aalen Johansen if competing risks are identified.

*8.5.2 Exit Survey (Analysis of implementation in Objective 1)*

In order to assess uptake of the exit survey, we will tabulate the number of those who agree to participate in the exit survey among those systematically approached. Uptake will be analysed by sociodemographic factors, facility, and study period using single predictor logistic regression to identify predictorws of non-response. Patient experience responses will be aggregated at the facility level through composite scores to facilitate cross-facility comparisons of uptake.

*8.5.3 BetterInfo Lite (Analysis of implementation in Objective 1)*

Given that the facilities have taken tracing of lost patients as routine activities, we will seek to assess the extent to which tracing is being carried out and documented (and therefore the extent to which we will carry out support activities). We will also seek to assess completeness of documentation. All will be done as simple propotions for a given interval of time. We will examine between clinic differences, and predictors of tracing implementation.

*8.5.6 FGD, IDI and Direct Observations*

Research memos will be used to analyse data from facility observations. However, we will use a combination of research memos and full transcription of audio-recorded data for in-depth interviews and focus group discussions. Research memos will be useful to get rapid feedback on implementation outcomes to the coaches and full transcription will be used to code for outcome themes. With the transcribed audio-recording data, we will translate (where appropriate), code, and computerize for analysis. If conducted in a language other than English, the interviews or focus group discussions will be translated into English with subsequent back translation or a second-party review of sections of the translation. The audio recordings, data collection tools and memos will be kept in a secure and locked file until the interviews are transcribed and the transcription is finalised, after which the recordings and notes will be destroyed. Identifying information will be removed during transcription in order to maintain confidentiality. Transcriptions will be kept in a password-protected computer file that will only be accessible to members of the research team for data analysis. Investigators will use thematic content analysis across all data to analyse the data using qualitative software (e.g. NVivo or Atlas TI). Analysis will begin during data collection so that topics for further exploration can be noted and incorporated into on-going fieldwork. Qualitative data analysis consists of searching for patterns in data and conceptualizing ideas that help explain the presence of those patterns. In order to achieve this, the following five steps maybe applied:

1. Reading for Content: Our analysis will begin with data reading until content becomes intimately familiar. As data are reviewed, emergent themes will be noted. Topics that previous research has not adequately addressed and ones that emerge unexpectedly will be explored in continued fieldwork.
2. Coding: A list of codes will be created based on identified themes and assigned to specific sections of text so that the text can be easily searched. Code definitions will be documented in a code book. Qualitative interviewers will be trained to apply the codes using qualitative analysis software. To ensure inter-coder reliability, 10% of data will be double-coded.
3. Data reduction: Once transcripts have been coded, we will work within each code to identify principal sub-themes that reflect finer distinctions in the data. This entails taking an inventory of what is related to the given code, capturing the variation or richness of each theme and noting differences between individuals or among subgroups.
4. Data display: Matrices and tables that categorize and display data will be used to help facilitate comparisons.
5. Interpretation: Once text has been read and coded, and central ideas extracted, we will identify and explain the core meanings of the data. We will search for relationships among themes or concepts identified and develop diagrams in order to map out relationships in the data.

8.6 Design of Objective 2 (Service Delivery and Patient Experience)

8.6.1 Population for Objective 2

The source population for Objective 2 will be patients receiving care the 34 health care facility and at each facility, in which we will sample patients to measure the effect of the intervention on service delivery and patient experiences. We will use a combination of exit interviews, trained patient exit interviews, RIAS and outcomes among the lost to measure these two constructs.

*Population for RIAS (Objective 2)*

The population of interest for RIAS recordings are HIV-infected adults (18 years or older) attending clinic for a routine clinical consultation as well as the health care workers that provide the clinical consultation, which includes medical officers, clinical officers, and nurses. We will seek to enroll about 480 patient and provider pairs for audio-recorded interactions overall from clinics (~3 pairs per clinic per wave). Recordings will only be conducted for those providers and patients giving written informed consent.To implement RIAS, research study staff will first enrol health care workers that conduct clinical consultation with patients (i.e., clinical officers, medical officers, and nurses). Patients attending the clinic for a clinical consultation will then be enrolled by research study staff from the waiting room prior to their encounter. Patients who consent to participate will then have their patient-provider interaction audio-recorded for future coding. To measure implementation of RIAS, study staff will record the number of eligible subjects (health care workers and patients), number of subjects approached, number enrolled, number of audio-recordings attempted, and number of encounters successfully recorded. Study staff will also collect data on the sociodemographic characteristics of enrolled health care workers and patients as well as document any challenges they encountered with audio-recording patient-provider interactions. We will also document the length of patient-provider interactions and the time required to code each interaction using the RIAS method.

*Table 3:* **RIAS:** Cell values represent total number of measurements per wave, per period

| **Wave** | **Period 1** | **Period 2** | **Period 3** | **Period 4** |  |
| --- | --- | --- | --- | --- | --- |
| **1** | **45** | **45** | **45** | **45** |  |
| **2** | **15** | **15** | **15** | **15** |  |
| **3** | **15** | **15** | **15** | **15** |  |
| **4** | **45** | **45** | **45** | **45** |  |
|  | **120** | **120** | **120** | **120** | **Total=480** |

*Population for Exit Interviews (Objective 2)*

The population is the same conceptually as in Objective 1.

*Population for QIPS (Objective 2)*

The target population for the QIP Exit survey component of our intervention are patients routinely in care and potentially LTFU patients who intend to return to back to care or who have an upcoming scheduled visit. After tracing LTFU patients as part of routine facility practices, we will recruit a convenience sample of LTFU patients who express intention to return to care. We will work with facility tracing staff to identify these patients. Among those who agree to participate in other study activities, such as SMS and exit surveys, we will recruit patients who are routinely in care. We will seek individuals with strong recall ability which can help ensure accurate data collection, and we will consider educational qualifications and language barriers practical considerations. All QIP participants will be 18 years of age or older. We seek to recruit approximately 15 for 15 encounters per clinic (15*34=510) per study wave (2040 total.). These participants will provide written informed consent.

## *8.6.2 Data collection for Objective 2*

*Measuring the Improved Welcome Back Experience and In Care Experience using* *QIPS*

We will use QIPS experience methods to measure the effect of our intervention on the experience of LTFU patients returning back to care, and of those in care. After tracing LTFU patients in the field through the existing programme, we will phone and recruit a convenience sample of LTFU patients who express intention to return to care or those who were wrongly classified as LTFU – that is, they are still in care. Prior to their return to care, LTFU patients who agree to participate as a QIPS will receive a brief training over 2-3 hours on the study instruments, on being natural yet observant during a clinic visit, and the concepts of quality care using the MoH’s Quality Improvement for HCWs in Zambia manual, the “gold standard”. Similarly, those still in care will receive this training. After this training, the QIPS will present themselves to the facility as they normally would to re-engage with care and will be instructed to make detailed observations regarding their experience. After their clinic encounter, they will then be asked to fill out a survey asking questions about their experience of returning to clinic. Participants will be given K100 for each training they attend and travel reimbursement for the clinic visit made. Just like the exit survey, this survey will also address issues around:

- The actual services offered by the facility to the patients
- The type of communication used with the patient
- The attitude and demenaor of the health care workers
- The extent to which barriers to care are solicited and addressed

*Measuring Patient Experience using Exit Surveys*

We will use the exit survey that was developed and validated during the pilot period and aims to capture the patient experience among patients currently in care. This survey was adapted from an existing tools, the Physician-Patient Communication Behaviours (PPCB) Scale (25), and also included questions that asked for patient reports on issues commonly cited in our previous work from the *Better Info* study. Throughout the study, we will seek to take a systematic (every *k*th) sample of patients leaving the facility after completing a clinic visit to assess their experience in clinic that day, where k depends on the facility volume and other logistical issues. At minimum, we will need to collect approximately 4,000 exit surveys during the study period to be adequately powered for this objective. As described in Objective 1, however, in order to obtain and report back facility-level estimates of the patient experience with some precision as part of the intervention, we may end up collecting up to 13,000 exit surveys across the study period. Patients will be informed of the the minimal risk involved in asking about their experience at the facility. Lay health care worker will then administer the tablet-based survey in a private area, guiding individuals through each question and the response options. Exit surveys will be conducted in participants preferred language (i.e., English, Nyanja, or Bemba). Results from the exit-interviews will be submitted via cellular network to a secure central database on a local secure server in Zambia.

*Table 4*: *Exit Survey*. Cell values represent minimum number of measurements per wave, per period

| **Waves** | **Period 1** | **Period 2** | **Period 3** | **Period 4** |  |
| --- | --- | --- | --- | --- | --- |
| **1** | **375** | **375** | **375** | **375** |  |
| **2** | **125** | **125** | **125** | **125** |  |
| **3** | **125** | **125** | **125** | **125** |  |
| **4** | **375** | **375** | **375** | **375** |  |
|  | **1000** | **1000** | **1000** | **1000** | **Total = 4000** |

Measuring Patient-Provider Communication using RIAS

We will use the RIAS method to objectively measure patient-provider communication in Zambia. RIAS is a thoroughly validated, well-established and extensively used approach (including in resource limited settings) to objectively parse the patient-provider communication into standardized dimensions and quantities(53–61). We will enroll patient and provider pairs from participating clinics, and we will audio-record patient-provider interactions during routine clinical consultations in their entirety. These will subsequently be coded using the RIAS coding system(61). The RIAS coding schema classifies each patient and provider utterance into one of 37 mutually exclusive categories of communication including statement types (encouragement, empathy, criticism, etc.), question types (open vs. close ended, question topics), and types of information giving (medical, psychosocial, etc.).

*Table 5:*

| **Approach** | **Depth** | **Reach** | **Purpose/Use** | **Sample Size** | **Implementation metrics** |
| --- | --- | --- | --- | --- | --- |
|  |  |  |  |  |  |
| QIP Survey | +++ | + | Welcome back experience of LTFU patients and clinic experience of informed patients | We anticipate an average of 10 per site, per study period for a total of approximately 1360 encounters | Proportion who complete QIP training and return to care visit |
| RIAS | +++ | + | Patient provider communications | 480 patient provider interactions from approxiamely 34 facilities during the 4 study waves periods. | Proportion of patient-provider pairs that have successful audio-recorded visit |
| HCW Experience | +++ | + | Measure experience of health care workers | Up to 30% of HCWs (or approximately 50 HCWs) per health facility | Proportion of HCWs reporting their experience on various domains |

Analysis for Objective 2

Overall analysis for objective 2 will seek to assess the impact of the intervention on each of these outcomes.

*Differences in patient experience as measured by exit interview*

We will assess changes in the mean level of satisfaction using a validated composite of the questions from the patient exit surveys. We will use mixed effects linear regression with clustering at the facility level to estimate the effect of the intervention on change in the patient satisifaction with their clinic visit. We will also assess changes in patient responses on key component variables such as proportion who report being happy with their visit, witnessing rude behavior, and having missing laboratory data using mixed effects linear or logistic regression, as appropiate. We will use the Hussey-Hughes method to account for the stepped-wedge study design.

*Difference in patient experience as measured by trained exit interviews*

We will also assess changes in the mean level of satisfaction using a composite of the questions from the trained patient exit surveys. We will use mixed effects regression with clustering at the facility level to estimate the effect of the intervention on change in the patient satisifaction with their clinic visit. We will also assess changes patient responses on key component variables such as proportion who report being happy with their visit, witnessing rude behavior, and having missing laboratory data using mixed effects linear or logistic regression, as appropiate. Among those returning to clinic, we will also assess changes in patient reports of being welcomed back at clinic and whether they were reinitiated on ART. We will use the Hussey-Hughes method to account for the stepped-wedge study design.

*Differences in the nature of patient-provider conversations as measured by RIAS*

Audio-recorded encounters will be coded using the RIAS coding system^38^. These codes will then be used to calculate a composite patient-centered communication score using previously validated methods (53,61). We will use mixed effects linear regression with clustering at the provider and facility level to estimate the effect of the intervention on change in the composite patient-centered communication score^29^. We will also assess changes in the percent of utterances by the provider versus patient, increases in the use of open-ended questions, and increases in the use of shared decision making. We will use the Hussey-Hughes method to account for the stepped-wedge study design.

*Changes in health care worker experience*

We will assess changes in the mean level of health care worker satisfaction using a validated composite of the questions from the health care worker surveys. We will use mixed effects linear regression with clustering at the facility level to estimate the effect of the intervention on change in the health care worker satisifaction. We will use the Hussey-Hughes method to account for the stepped-wedge study design for wave and period.

## 8.7 Overview of Objective 3

In objective 3, we will seek to assess the downstream effects of targeted facility improvement strategy in PCC on retention, treatment success (a composite metric that includes viral suppression, care engagement, and mortality) and mortality alone.

The target population will be all patients who have an encounter in the first six months of wave 1 and 4. We restrict to waves 1 and four because we will not be able to assess the effect of the intervention at on year in any one individual who has experience both the intervention and control conditions. The fact that retention and viral load are the product of a relatively long-term experiences, we will avoid using waves 2 and 3 where few, if any, patients will experience a year of the intervention or the control conditions. We restrict to the first year because this will allow one 18 months of follow up.

The source population will be a sub-cohort because in the entire clinic population, record keeping and loss to follow-up often lead to misclassification of outcomes. We will therefore enrol a sub-cohort at the onset and actively assess outcomes in this group at approximately 18 months. This will allow superior classification of outcomes.

*8.7.1 Population for Objective Three*

*8.7.1.1 Sub-cohort for treatment success*

The population for primary study endpoint will be a probabilistic sample of patients who meet the following inclusion criteria: make a visit to one of the study facilities in the first 6 months of the 24 month study period, ever been on ART (including those starting on that very visit not planning on leaving the area, resident of the catchment area of the clinic, and they agree to having a viral load done 12-18 months after enrolment in this viral load sub-cohort. We will also restrict to facilities in wave 1 and 4 in order to allow at a minimum of 12 months of exposure the intervention or control conditions at the time out outcome assessment. We will seek written informed consent for enrolment in the treatment failure the sub-cohort. We will select up to approximately 60 patients at each clinic at each of the 16 facilities in waves 1 and 4, for a total maximum of approximately 960 viral loads..The reason for choosing a subcohort is that in large health systems trials outcome classification in the setting where record keeping can be a challenge and misclassification an issue, identification of a sub cohort who will be representative of all, but in whom more intensive outcome ascertaintment can be done if needed will be useful.

*8.7.1.2 Full cohort for retention in care*

We will, nevertheless, also assess endpoints on the entire clinic based cohort represented in the EMR, using outcomes that can be assessed in the electronic medical record, specifically retention using visits, transfer and passively collected vital status. Since all patients who experience an encounter during the 24-month study period will be examined for the retention endpoint, which we anticipate based on current program data to be as many as 170,000. We seek a waiver of consent for measurement of clinical care and outcome data in this cohort. Reviewing patient outcomes as recorded in routine medical records is of minimal risk. It is not possible to seek written consent from the entire cohort of patients. We will treat known deaths as lapses in treatment.

*Figure 1*

Collect baseline VL

*8.7.2 Measurements for Objective 3*

*8.7.2.2 Measurements ~~for Viral Load~~of treatment failure*

~~At the time of follow-up to be assessed a~~At a target of 15 months, we will assess treatment failure. We use 15 months for practical reasons. Although our original target was to assess treatment outcomes at 12 months, we instituted an additional three month period for ascertainment of viral load because 12 months after initial study start coincided with a period of health systems emergency in Zambia during the first wave of COVID-19 that made assessment of patient outcomes a challenge and also led to concomitant delay in study roll out (and extension of period two). We will accept data for a 15-month endpoint using information in at a minimum approximately a six month window around the 15-month point – recognizing that we will leverage and use clinically obtained viral loads when possible, and the timing of such measurements may or may not coincide with observation time in this study. Treatment failure is a defined as a composite of (1) unsuppressed viral load (> 400 copies/ mL), (2) documented disengagement in care, (3) switch of regimen for elevated viral load, or (4) death. We will treat documented prospective transfers to another facility as censored observations. For those who are traced and found to be transferred to another facility, if first visit is 14 or more days after last assigned return date at original clinic, patient will be considered a treatment fai~~la~~ure, as well.

Viral load will be assessed using the Cobas CAP/CTM assay. We will collect baseline viral load from our cohort in wave 1. At the end of one year, we will check to see if patients have a viral load done in the course of routine clinical care in the correct window of time. If they do, then we will use that as the outcome value. If they do not, we will either encourage VL collection by clinic if clinical indications exist, or ask the participant to return to the facility and have their blood collected as part of the facility’s routine collection. We will explore whether the facility has the capacity to collect samples in the field and will make use of that capacity if it exists. If this does not coincide with a routine clinical visit, the study will cover costs of transportation and time incurred by the patient to have their viral load measured. We will use the viral load measurement closest to 15 months of follow-up if multiple exist.

If patient does not have a viral load recorded in the interval of interest, we will seek to ascertain engagement through patient tracing to assess care status. Extended outcome ascertainment will trace patients intensively, which will leverage our previous experience identifying patient vital status and care status. Informants to assess patient outcomes can include health care workers, community members, family and others. In this process if the patient is found to have died, is out of care or off treatment, they will be considered a treatment failure as well. If the patient if found alive and in care at another facility, we will treat that outcome as a failure if first visit to new clinic is more than 14 days late than anticipated return to original clinic. For self-reported transfers, we will validiate with outreach to a reported facility in a sample to verify patient report.

*8.7.2.1 Measurements for Retention*

For analyses using the full cohort of individuals in the EMR, we will assess retention using appointment and visits as recorded in the EMR.

## 8.8 Analysis for Objective 3

The primary outcome for objective 3 is treatment success 15 months after initial visit during period 1 of the study. For the analysis of this primary outcome we will assess the fraction with treatment success or not at 15 months using logistic regression. Random effects will be used to address within site correlation. Adjustment will be made for composition of the cohort recruited, in particular for age, sex, and time on ART at first visit during the study period. Sensitivity analyses for the primary analysis will include altering interval of absence considered a failure, and classifying all transfers out as either succeses for failures.

For retention, we will calculate the cumulative incidence of a missed visit by 30 or more days at up until 15 months of follow-up time. We will use the Kaplan Meier approach, stratified by the intervention with a log rank test for significance. We will explore competing risks for transfers and mortality if those outcomes are not trivial in magnitude. In additional analyses we will use a multivariable regression, starting with Cox proportional hazards model if assumptions about data are met, to assess the effect of the intervention on retention. We will address clustering by site with a random effect term. In secondary analysis, we will adjust for facility and patient characteristics. data are prevalent, we will consider the use of inverse probability weights or imputation to address missing outcomes.

## 8.9 Design of Objective 4

## *8.9.1 Study population for Objective 4*

A primary objective of this study is to evaluate the cost-effectiveness of the PCC intervention, from both a health systems and a patient perspective. Primary components of the cost of the PCC intervention are expected to include:

Implementation Costs: Up-front costs required to implement the PCC intervention, including: (a) development and implementation of exit interviews; (b) development and implementation of an SMS platform to perform surveys describing the patient experience; (c) development of BetterInfo Lite to trace and interview patients who are lost to follow up; (d) tailoring of a patient-centered skills curriculum to the local context; and (e) costs (i.e., time and training) of hiring and training practice facilitators (“coaches”) to feed data back to clinics.

Ongoing Delivery Costs: Costs of maintaining the PCC intervention to a high level of fidelity once implemented, including: (a) delivery of exit interviews (patient and provider time, data collection apparatus); (b) delivery of SMS-based surveys (SMS costs, patient time, analysis time); (c) delivery of BetterInfo Lite (provider time to contact patients, patient time after being contacted); (d) practice facilitator salary and any required supplies; (e) delivery of patient-centred care principles and skills curriculum; and (f) provision of incentives to providers and facilities based on performance feedback.

Quality Assurance/Improvement Costs: Costs to modify and improve the PCC intervention over time, in response to implementation, including: (a) any modifications of the PCC intervention that might need to be made to tailor to the local context; and (b) ongoing monitoring and evaluation (excluding research-based costs, but assuming that any implementation would require some M&E). Changes in Activities as a Result of the PCC Intervention: If effective, the PCC intervention will result in some changes in both (a) provider and (b) patient experience.

## *8.9.2 Data Collection for Objective 4*

We will collect costs in three different manners: (a) one-time, centrally, among PCC staff; (b) one-time, at each of the 48 clinics; and (c) once per wave of the stepped wedge trial, in each of eight clinics that will be purposively sampled to provide a range of urban/rural status, healthcare system level, staffing ratio (e.g., number of vacant positions), geography, and patient volume.

Data collected one time, centrally:

Key informant interviews: We will interview key personnel (primarily study staff) involved in implementing PCC (for example, those responsible for developing and implementing the SMS platform, BetterInfo Lite, exit interviews, etc). This will be done from a top-down perspective, utilizing existing templates from other similar studies. The key objective of these activities is to estimate the total cost of implementing each component of the PCC intervention, in isolation and in combination. Costing will include both the development and the implementation of the PCC intervention.

Budgetary analysis: We will use a standardized cost data collection form to estimate the resources required to 1) develop the PCC intervention program and 2) implement and deliver each component of the PCC intervention. This will be done in parallel with the key informant interviews, as the key informants are likely those who can also help to evaluate the relevant budgets. Costs will be categorized into standard groupings (e.g., overheads, building space, equipment, human resources, consumables) and will be extracted from relevant financial records.

Data collected one time, at each of the 34 study clinics:

Budgetary analysis: We will modify the standardized cost data collection form used at the central level to also estimate the resources required to implement and deliver each component of the PCC intervention in each clinic. Costs will be categorized into standard groupings (e.g., overheads, building space, equipment, human resources, consumables) and will be extracted from relevant financial records.

Coaching log review: We will train coaches to complete log entries every time they visit a facility, capturing the total amount of time spent, number of staff and patients visited, actitivies conducted and all issues that arose. These data will be used to inform variability in practice and resource use across all clinics.

Data collected in each phase of the stepped wedge, at approximately 6 purposively sampled clinics:

Observation of resources used: We will directly observe the delivery of each component of the PCC intervention (or control condition) in each of approximately 6 clinics, for a total of one week per clinic per phase of the trial. Observation will include staff time and documentation of any other resources (e.g., tablets, building space) used. This activity will involve observing clinic staff members (not patients per se) and will enable us to assess variability in delivery, both clinic-to-clinic and day-to-day within and across the clinics included in this study. We will employ time and motion studies where feasible and time-use surveys otherwise. By coding activities into specific categories, we will be able to estimate changes in health care workers’ workflow (e.g., direct patient interaction times and use of time for non-patient activities). We will verify these observations against estimates of patient time spent within the clinic (accomplished by asking patients to record their arrival time, time spent waiting between respective services, and time of exit). These data will also be checked against data from the coaching logs as described above. We will work with the qualitative data collection team to incorporate questions relevant to costing into focus group discussions and in-depth interviews.

***8.9.3 Data analysis for Objective 4***

We will inflate all costs to year 2020 using the Zambian GDP deflator and then convert to US dollars using contemporary exchange rates. We will estimate the unit cost of each activity, disaggregated to the extent possible (for example, cost of implementation will be reported as a total cost, but also as a cost per clinic and cost per patient reached, as a function of the duration of the intervention and the number of patients in each clinic who are eligible). Where feasible, unit costs will be shared with the Global Health Cost Consortium (GHCC) and compared to existing estimates from other settings. We will then perform a cost-effectiveness analysis, according to the principles laid out in the Gates Foundation Reference Case for Economic Evaluation. Cost-effectiveness will be evaluated from both a healthcare and modified societal (i.e., healthcare plus patient) perspective. We will estimate the incremental cost and incremental effectiveness of the PCC intervention as a whole, enabling us to calculate the incremental cost-effectiveness ratio (ICER) for this intervention. For our effectiveness estimate, our primary analysis will reflect the analysis performed for the CommART study, in which we use a Markov model of patient outcomes to estimate disability-adjusted life years (DALYs) averted by the intervention. To arrive at this estimate, we will use the primary outcome of the study (treatment failure~~)~~ and project patient outcomes over a five-year horizon under the intervention, compared against a counterfactual of no intervention. We will discount both costs and effectiveness by 5% per year. We will conduct extensive sensitivity analyses, based on the effectiveness results of the primary study, to consider components of the PCC intervention that are most important to cost-effectiveness and to evaluate the potential drivers of cost-effectiveness. We will also use our estimates of clinic-level variability in a probabilistic uncertainty analysis to describe the level of uncertainty in our estimates of cost-effectiveness.

9.0 ETHICAL CONSIDERATIONS

This protocol, the informed consent documents, and any subsequent modifications will be submitted for review and approval by the UNZA Biomedical Research Ethics Committee (BREC). Also, the University of Alabama in Birmingham (UAB) Institutional Review Board (IRB) will provide regulatory oversight through a reliance agreement with the other collaborating US institutions. We request an expedited review. This is a minimal risk study designed to evaluate the effect of a three-part PCC intervention (described in section 6.0 under intervention design above) through a stepped wedge cluster randomized trial design. We will evaluate the implementation, cost effectiveness of the intervention on patient experience and clinical outcome measures. Procedures with health care professionals will utilize their expertise and experience to understand how patient-centred care can be delivered in public health settings. We will not collect HCWs’ personal or sensitive information. We will collect audio, paper, electronic and SMS data on patient perceptions of their clinic experience. We will implement reasonable and appropriate protections to ensure minimal risks related to invasion of privacy and breach of confidentiality for SMS, QIP client and tracing methods as described below. Further information on participant risks, benefits and other considerations for human participants are addressed in Section 5.1 below.

We will address the ethical dilemmas of using mHealth in research involving people living with HIV in both design of access portal and data collection methods (13). To ensure that the data generated and communicated through Mhealth are useful and interpretable to end users, we will design information access portals for navigability, quality, presentation, and accuracy. Collection of mobile numbers will follow Zambia Information and Communications Technology Authority (ZICTA) guidelines that require patients to opt into the study to receive and send data by phone (14). We will implement strategies to overcome barriers to participation such as inability to use mobile phone features, for e.g., short messages services (SMS) or unstructured supplementary service data (USSD). As this could exclude some older or less educated patients - indeed, perhaps those most in need of supplemental patient support – we will implement strategies such as training patients on using the SMS system. In addition, we will also consider including patients with less education background (semi illiterate) to participate in the SMS Survey. Patients who opt out will have the option to participate in the exit interviews or serve as as will patients using no or shared mobile phones. We will validate ownership of mobile numbers by sending an SMS at enrolment and requesting an immediate response confirming ownership of the mobile number. Mobile numbers will be kept separate from other identifying information such as name and ART number, and will be linked by codes held by the study site manager. Depending on feasibility and patient preference, we may use USSD to collect information. The USSD will be initiated by the patient who will dial a number that starts with * and ends with #. The USSD allows for patient autonomy, privacy and confidentiality being patient-initiated and limiting transactions to one session unlike SMS that can be retrieved at any time. If using SMS, the first message will ask the patient to provide parts of the linking code to establish that the messages are being read by the intended recipient. This will also ensure that questions regarding clinic visits do not result in unintended disclosure of the presence of a medical condition even if HIV is not specifically mentioned. While the study will ensure participant confidentiality is maintained from transmission of data to local storage of data through restricted entry into rooms and electronic databases, participants will be advised to delete the SMS survey from their phone once completed. To avoid infringement on personal time, we shall set time limits for data collection (e.g., 9 a.m. to 9 p.m.). Participants who enrol into the SMS component of the study may be rewarded with a small amount of talk time. All costs relating to the sending and receiving of SMSes will be covered by the study.

Our proposed Quality Improvement Patient Exit Survey methodology does not expose HCWs and QIP Clients to additional cost, risks or burdens and is designed to produce scientifically valid data that will greatly increase our understanding of patient experience and provide facilities with the information they need to prioritize and initiate quality improvement efforts. The main ethical issues with use of QIP are those of deception, privacy and consent from HCWs. To address these problems, we will seek approval to conduct QIP visits from the Ministry of Health, as well as assent from provincial, district and facility authorities. Before commencing field work, facility-in-charges will be informed at a central sensitization meeting of impending QIP Client study visits, but the details of the dates and names of the specific health facilities will not be provided (15). At this meeting, information regarding the study and procedures will be presented. All facilities will have the option to opt out. It will be explained to the facility-in-charges that they will be responsible for explaining facility participation in this study to their staff, and that the purpose of using a QIP is to better understand the patient experience. Facility-in-charges will be asked to explain to their staff that no individual health care provider would be identified. Nor would it be possible to identify individual HCWs since the questions are about the patients’ overall visit experience from triage to ART pick-up during which time they could interact with any combination of lay health care workers, adherence counsellors, nurse, clinical officer, doctor, laboratory personnel, pharmacy technician and other support staff such as cleaners. Further, codes will be used to identify facilities during fieldwork and analyses. Nonetheless, staff members uncomfortable with being part of the QIP procedures would have the right to opt out. They would communicate this to study staff via the in-charge. Study staff will ensure that QIP do not reflect on interaction with staff who decline participation.

We will be training patients to be QIP in order to get an objective review of patient experience in the clinic. QIP will present as per usual for their routine care in their ART clinic but will be trained in the exit interview and SMS instruments. This is because in the context of Zambian HIV care and treatment program, patients cannot rotate through facilities without disrupting systems. With QIP undergoing routine without any simulation or manipulation, many ethical protections are provided – the length of the appointment would be appropriate, clinical resources and time would not be wasted, patients will not be exposed to unnecessary costs, procedures and treatment, and the interaction will be relevant, credible, practical and safe. Hence, patients will be trained not to alter their behaviour and not to manipulate the interaction in any way to allow for collection of more complete and accurate data on patient experience. Using up to 50 patients for 100 observations will also provide information on diverse experiences based on type of visit, day of the week, number of HCWs involved in the interaction, and patient volume. Patients who express interest in serving as QIP will be asked for their consent informed by full disclosure including on associated risks (16). To further protect patient and provider privacy, exit and SMS interviews conducted by QIP will be presented as aggregate data containing no personal identifiers.

Patient tracing will follow the tracing procedures that are currently implemented in standard clinical practice. This includes finding the patients, identifying their outcomes, and encouraging them to return to care. When relevant, this also means speaking with family members of the deceased to determine the patient outcome. As demonstrated by the Better Info Study, we conduct tracing with the utmost care and concern for patient confidentiality. This includes practices such as training of patient tracers, stating only health-related purposes or other socially acceptable, non-identifiable reasons for any visit, and consulting health care workers and key stakeholders at the study-affiliated clinics to identify other important tracing practices to maintain confidentiality. Any further activities occur only if informed, voluntary consent is granted by the potential participant.

The following sub-sections elaborate the human subject protections by which this study will abide.

9.1 Informed Consent

Permission will be sought from national, provincial, district and facility Ministry of Health authorities to conduct the study in public health facilities. The study will follow standard Ministry of Health guidelines to engage with staff, patients and patient records.

Written informed consents will be procured at two levels (See Table 6). Individual level written informed consent will be sought from patients and HCWs for participation in FGDs and in-depth, cognitive, SMS and exit interviews. As outlined in Section 1.5, QIP will also be asked for their written informed consent. Facilities through the facility in charge will have the option to opt out of the direct observations and quality improvement procedures. .We seek waiver of written informed consent for the following study activities:

- SMS and Exit Surveys: The questions posed in the SMS and Exit Surveys involve minimal risk to the participants and do not reveal anything about individual HIV care.
- Direct Observations which take place in private setting in the facility since the the focus of the observation relates to the manner in which the services are provided. This is explained in detail under *section 8.4.6 above*.
- Collection of clinical care and outcome data from Smart Care: We seek a waiver of consent for measurement of clinical care and outcome data in the population for retention outcome cohort. Reviewing patient outcomes as recorded in routine medical records is of minimal risk. It is not possible to seek written consent from the entire cohort of patients, yet all records are required to yield complete and unbiased outcome data(*section 8.7.2)*

Table 6. Summary of study activities requiring written informed consent (facility-level highlighted)

| **Study Activity** | **Participants** | **Approximate Sample Size** | **Informed Consent Procedure** | **Risk Level** |
| --- | --- | --- | --- | --- |
| Patient experience SMS: collect phone number, voluntarily respond to questions about experience at health facility, HIV not explicitly mentioned | Patients | 40,800  (300 x 34 facilities x 4 6-month periods) | Waiver of consent | Minimal |
| Patient experience exit survey: Upon leaving facility, respond to questions about experience that day, survey done on tablet computer administered by community liaison officer | Patients | 13,600  (sub-sample of those recruited for SMS survey, 100 x 34 facilities x 4 6-month periods) | Waiver of consent | Minimal |
| Viral load | Patients | Approximately 2400 | Voluntary, individual, written consent | Minimal |
| RIAS | Patients and Health Workers | 480 patient-health worker pairs | Voluntary, individual, written consent from health care workers and patients | Minimal |
| Patient survey with traced patients beyond standard tracing procedures (BetterInfo ‘lite’) | Patients | 10% of lost to follow up patients every 6 months at all 34 facilities | Voluntary, individual, written consent | Minimal |
| QIP visits | Patients | 1,360 | Voluntary, individual, written consent | Minimal |
| Quality Improvement efforts, including QIPs, patient centred care curriculum, use of PPEMS data, coaching, human-centred design methods, facility quality improvement implementation and evaluations of the above | Health care workers | Up to 34 facilities | Study MoH approval letter | Minimal |
| In depth Interview with patients to capture patient experience of intervention | Patients | Up to 40 IDIs | Voluntary, individual, written consent | Minimal |
| Focus Group Discussion with Healthcare workers to understand the interventions | Health care workers | 24 FGDs | Voluntary, individual, written consent | Minimal |
| Direct Observations | Patients and Healthcare workers | Up to 20 direct observations | Facility-level verbal consent/approval for public space observations  Written consent from providers in screening and counselling rooms, Waiver of consent from patients in screening and counselling rooms with right to refuse participation | Minimal |
| Collection of clinical care and outcome data from SmartCare | Patients | All patients seeking HIV care at facility | Waiver of consent | Minimal |
| Measuring provider experience | Health care workers | Up to 30% of HCWs (or 50 HCWs) per health facility per health facility | Waiver of consent | Minimal |
| Measuring provider experience during Covid19 | Health care workers | Up to 30% of HCWs (or 50 HCWs) per health facility per health facility | Waiver of consent | Minimal |

Ministry of Health approval will be sought to enable facility staff to participate in study procedures. The sister-in-charge or designee will confirm willingness to allow the facility to participate in the privacy of their office. If willing, they will be given information to provide to their staff including on the main aim of the study which is to increase patient engagement. They will be asked to inform staff of the objectives of all the procedures, which is to provide management and HCWs with information on participant’s perspectives, health system issues and clinic performance. These perspectives along with coaching will help facilities prioritize and initiate quality improvement programs. Facilities and HCW will be made aware of the types of questions we will ask of the QIP, number of visits, and that they will not be able to tell QIP from other patients as they will be given the same Exit and SMS surveys. For all procedures, they will be told of the reporting formats, channels of communication, basis of patient recruitment and the benefits to their work and facility (63). Facility staff will have the option to decline being part of QIP procedures.

Individual consents will be sought from HCWs and patients. HCWs who express interest post-staff meeting announcement and during follow-up, will be individually consented in a private room. Patients will be recruited from the clinic waiting room. Research staff will approach each patient, provide an outline of the study and if interested, invite them to a private room to complete informed consent processes. QIP will be recruited in a similar manner. They may also be identified through ART clinic appointment schedules for the four months of data collection. Those identified through appointment schedules will be contacted using standard practices followed in ART clinics. QIP will also be identified from among LTFU patients traced for this study. Prior to informed consent, the study tracing teams will only conduct activities as outlined in the current tracing by phone and in person procedures. Patient tracing will be adapted from existing procedures that are currently implemented in standard clinical practice in Ministry of Health facilities. This includes finding the patients, identifying their outcomes, and encouraging them to return to care/remain engaged in care. When patients who are traced are found, they will be encouraged to re-engage in care if they are disengaged, supporting ART programmatic objectives. In all cases, study activities will occur only if informed, voluntary consent is granted by the potential participant.

Participants who meet eligibility criteria, express willingness to participate, and provide written informed consent will be enrolled. Consent processes will be done in English, Nyanja and Bemba as per patient/HCW preference. The informed consent will describe the purpose of the study, the procedures to be followed, and the risks and benefits of participation. The potential participant will be given a chance to answer all questions. A copy of the consent form will be offered to the study participant. Participants may choose to participate in none, all the study procedures – FGDs, IDIs, Exit Interviews, SMS Interviews, Direct Observations and QIP visits.

9.2 Risks to Participants

All interviews and discussion have the potential to make participants embarrassed, worried, anxious, or uncomfortable when reporting on their interactions, the clinic vital signs or when they learn regarding the possibility of an unannounced QIP visit.

Use of SMS poses a risk to privacy in that patients may not have control over the extent, timing and circumstances of participation. Confidentiality could be compromised should the SMS data entry be observed or, if retained as SMS, read by or forwarded by an unintended recipient. Questions referring to clinic visit may disclose the presence of a medical condition even if HIV is not specified.

QIP procedures pose a risk to privacy of HCWs, who will not have control over the scope, timing and frequency of visits. Confidentiality could be compromised if individual patient and HCW can be identified by the data collected. Recall and Report bias is possible if surveys are too long or complicated. Validity maybe further compromised only factual (e.g., cleanliness) or only subjective (e.g., feelings) data are collected; or a small range of experiences are captured.

Possible risk of involuntary disclosure of HIV status and inadvertent ‘outing’ of participants may occur when tracing LTFU patients in their community settings. There might be discomfort among relatives discussing patients who died in the 12 months leading up to the interview.

9.3 Methods to Minimize Risks

We will train all research staff to be discrete and professional, ensuring that patient confidentiality is maintained at all times. Research staff will be trained to ensure participants are comfortable and to assure continual informed consent during study procedures. They will be trained in appropriate implementation methods. We will hold a sensitization with facility in charges to alleviate any concerns relating to staff performance and its impact on employment security. We will inform the facility that no individual level information or identifiers will be collected. While conducting surveys, interviews and FGDs, the research staff will be trained to be sensitive to patient concerns and conduct procedures away from a public setting.

The form, content and frequency of SMS messaging will be determined in consultation with MoH, patients and HCWs. As mentioned in Section 1.5, we will set limits to the hours during which data will be collected (e.g., 9 a.m. to 9 p.m.) to protect patient privacy. We will delink SMS data on patient experience from patient medical records and identifying data. If using SMS, patients will be provided with unique passwords that they can easily recall to begin the SMS survey. All data will be protected as per CIDRZ, MoH, ZICTA and international SMS security and the International Organization for Standardization (ISO) guidelines and directives on appropriate safeguards of data collected for mHealth research and implementations (64).

As mentioned in Section 1.5, we will get permission from MoH authorities at provincial, district levels and informed consent from facility -in-charges for QIP procedures. We will not collect HCWs or patient’s identifiable data or medical records. Patients will be trained to observe events and respond to Exit and SMS surveys in a uniform, systematic manner (65). Both surveys will be validated, short, and simple and will reflect information off each other making recall and reporting easy. They will include both objective (e.g., did HCW greet you today) and subjective (e.g., satisfaction) questions will be included to help with interpretation of the experience. Responses will be placed in unidentifiable database for analysis. All the data will be collected and protected according to standard procedures and analysed in the aggregate without any reference to the individual HCW's and patient’s information. Finally, all identifying information is destroyed at the conclusion of the study.

We will use our previous experience with patient tracing to ensure patient confidentiality is maintained at all times. We will consult health care workers in facilities where we are working for other local considerations to ensure patient confidentiality at all times. Tracers will be well trained and will only state health-related purposes or other socially acceptable, non-identifiable reasons for any household visits. We will also employ same gender trackers or mixed gender tracker pairs whenever possible. They will not wear any identifiable clothing or badge when making household visits. Different tracers will rotate through defined geographical areas so that the community members do not identify them as related to the study. The research staff will be trained to be sensitive to disclosure and discomfort, for example, as discussing deceased household members.

9.4 Anticipated Benefits to Participants

No direct benefits to participants are expected. Participants may benefit from improvements in quality of care provided and may decide to remain engaged in care. Those disengaged from care may benefit from tracing and follow up by trained counsellors and may decide to re-engage in care. Health care workers and facilities may benefit from understanding patient expectations and from learning how data can be used to interpret and improve clinic practices. Patients may also benefit from the knowledge that their perspectives are valued for improving care delivery. Though benefits to the patient are minimal, the associated risks with participation in our study are equally small and thus reasonable in comparison. The Zambian Ministry of Health will benefit from the coaching provided by the research team.

9.5 Privacy of Individuals

Project staff will be trained in fundamental ethical principles and good research practices. As such, the need to respect persons and their privacy will be emphasized and shall constitute part of the Standard Operating Procedures (SOPs) for field work. Patients will have full disclosure on who will access the data they provide, where it will stored, how it will be reported and how the results will be used so that they can decide if they want to share their information(66). Participants will have the right to refuse to participate or set an alternate time. They will have the right to decline to answer any question that feels obtrusive. Audio-tapes will only be used with participant permission. Only that information that will be used in analysis will be maintained in study-specific electronic databases that shall not capture any individually identifying data. No unauthorized transmission of such information shall be allowed. As earlier indicated, information collected will be restricted to the study team, and if needed, the ethics review committees. To the extent allowable by law, clinical information will not be released without written permission of the participant, except as necessary for monitoring by the FDA, the Office for Human Research Protections (OHRP), the local IRB or Ethics Committee (EC). All datasets created will include complete codebook/s and metadata dictionaries providing necessary context for appropriate use of a given dataset. All data will be stored securely on CIDRZ servers and access to intervention data will be restricted to select CIDRZ and study staff.

9.6 Confidentiality of Data

Completed questionnaires and all study information including patient phone numbers shall be kept confidential. All study-specific evaluation forms, reports, and other records will be identified only by a coded number to maintain participant confidentiality, unless being shared for purposes of clinical care as outlined in this protocol. All records, including audio will be kept in a secured area that will be locked at all times. All computer entry and networking programs will be done with coded numbers only. All staff involved in this work will sign the CIDRZ confidentiality agreement, which prohibits them from sharing confidential information.

9.7 Study Discontinuation

The study may be discontinued at any time by the sponsors, the Ministry of Health, or CIDRZ as part of their duties to ensure that research participants are protected. Only the data collected up to this point will be used for analyses. CIDRZ and protocol-affiliated investigators and staff members will not use these data for other, unrelated analyses without the express permission of the MoH.

9.8 Data Use

These data will be used to help facilities implement patient centred care using human centred design. The planned procedures will facilitate the monitoring of data quality and service delivery, thereby providing opportunities for local capacity building. Information will be continually shared with the facility. Interim and final findings will be shared with MoH and other relevant stakeholders. Donors, MoH and IRBs will receive reports as per their stipulated report schedule. Findings will be disseminated at national and international meetings and through abstracts, oral presentations and peer-reviewed papers. We will de-identify the data before sharing with MoH, other stakeholders,donors and IRBs. We will seek additional, separate ethical review for any analyses falling outside the objectives of our current protocol.

10.0 TIMELINES

The study timeline is as follows:

| **Description** | Y1 Wave 1 | Y1 Wave 2 | Y2 Wave 3 | Y1 Wave 4 |
| --- | --- | --- | --- | --- |
| Study protocol approved |  |  |  |  |
| Study Staff trainining |  |  |  |  |
| Sensitization of all intervention and control facilities |  |  |  |  |
| Intervention Sensitization |  |  |  |  |
| Phased implementation of Objective 1, 2, 3, 4 |  |  |  |  |
| Data collection evaluating the effect of PCC intervention |  |  |  |  |
| Viral load |  |  |  |  |

| **Month of Study** | **Activities** |
| --- | --- |
| 1 - 2 | Regulatory approval staff training, study sensitization and facility deployment |
| 3 - 8 | Wave 1 implementation of Objective 1, 2, 3 and 4 |
| 9 - 14 | Wave 2 implementation of Objective 1, 2, 3 and 4 |
| 15 - 20 | Wave 3 implementation of Objective 1, 2, 3 and 4 |
| 21 - 26 | Wave 4 implementation of Objective 1, 2, 3 and 4 |

11.0 BUDGET

| **Item** | **24-month Primary Budget** |
| --- | --- |
| Study Staff Costs | K 20,000,000 |
| Travel | K910,000 |
| Consulting | K300, 000 |
| Equipment and Supplies | K480,000 |
| **Total** | **K21,690,000** |

12.0. REFERENCES

1.	Duncombe C, Rosenblum S, Hellmann N, Holmes C, Wilkinson L, Biot M, et al. Reframing HIV care: putting people at the centre of antiretroviral delivery. Tropical Medicine & International Health [Internet]. 2015 Apr [cited 2017 Jun 13];20(4):430–47. Available from: http://doi.wiley.com/10.1111/tmi.12460

2.	Holmes CB, Sikazwe I, Sikombe K, Eshun-Wilson I, Czaicki N, Beres LK, et al. Estimated mortality on HIV treatment among active patients and patients lost to follow-up in 4 provinces of Zambia: Findings from a multistage sampling-based survey. Rosen S, editor. PLOS Medicine [Internet]. 2018 Jan 12 [cited 2018 Feb 14];15(1):e1002489. Available from: http://dx.plos.org/10.1371/journal.pmed.1002489

3.	Topp SM, Mwamba C, Sharma A, Mukamba N, Beres LK, Geng E, et al. Rethinking retention: Mapping interactions between multiple factors that influence long-term engagement in HIV care. PLOS ONE [Internet]. 2018 Mar 14;13(3):e0193641. Available from: https://doi.org/10.1371/journal.pone.0193641

4.	Musheke M, Bond V, Merten S. Individual and contextual factors influencing patient attrition from antiretroviral therapy care in an urban community of Lusaka, Zambia. Journal of the International AIDS Society [Internet]. 2012 Jun 14 [cited 2018 Mar 16];15(3(Suppl 1)). Available from: http://doi.wiley.com/10.7448/IAS.15.3.17366

5.	Merten S, Kenter E, McKenzie O, Musheke M, Ntalasha H, Martin-Hilber A. Patient-reported barriers and drivers of adherence to antiretrovirals in sub-Saharan Africa: a meta-ethnography. Tropical Medicine & International Health [Internet]. 2010 Jun [cited 2018 Mar 16];15:16–33. Available from: http://doi.wiley.com/10.1111/j.1365-3156.2010.02510.x

6.	Man J De, Mayega R, Sarkar N, Waweru E. Patient-Centered Care and People-Centered Health Systems in Sub-Saharan Africa: Why So Little of Something So Badly Needed? International Journal of [Internet]. 2016 [cited 2017 Sep 18]; Available from: http://www.ijpcm.org/index.php/ijpcm/article/view/591

7.	Bedelu M, Ford N, Hilderbrand K, Reuter H. Implementing Antiretroviral Therapy in Rural Communities: The Lusikisiki Model of Decentralized HIV/AIDS Care. The Journal of Infectious Diseases [Internet]. 2007 Dec 1 [cited 2017 Dec 14];196(s3):S464–8. Available from: https://academic.oup.com/jid/article-lookup/doi/10.1086/521114

8.	Beach MC, Keruly J, Moore RD. Is the Quality of the Patient‐Provider Relationship Associated with Better Adherence and Health Outcomes for Patients with HIV? Journal of General Internal Medicine [Internet]. 2006 Jun 1 [cited 2017 Sep 18];21(6):661–5. Available from: http://onlinelibrary.wiley.com/doi/10.1111/j.1525-1497.2006.00399.x/full

9.	Flickinger T, Saha S, Moore R. Higher quality communication and relationships are associated with improved patient engagement in HIV care. Journal of acquired [Internet]. 2013 [cited 2017 Sep 18]; Available from: https://www.ncbi.nlm.nih.gov/pmc/articles/PMC3752691/

10.	Stewart M, Brown JB, Donner A, Mcwhinney IR, Oates J, Weston WW, et al. The Impact of Patient-Centered Care on Outcomes. 2000 [cited 2017 Dec 14];49(9). Available from: https://www.researchgate.net/profile/Wayne_Weston/publication/12292586_The_Impact_of_Patient-Centered_Care_on_Outcomes/links/004635260327e8a432000000/The-Impact-of-Patient-Centered-Care-on-Outcomes.pdf

11.	Stewart MA. Effective physician-patient communication and health outcomes: a review. CMAJ: Canadian Medical Association Journal [Internet]. 1995 May 1;152(9):1423–33. Available from: http://www.ncbi.nlm.nih.gov/pmc/articles/PMC1337906/

12.	Kruk ME, Riley PL, Palma AM, Adhikari S, Ahoua L, Arnaldo C, et al. How Can the Health System Retain Women in HIV Treatment for a Lifetime? A Discrete Choice Experiment in Ethiopia and Mozambique. PLOS ONE [Internet]. 2016 Aug 23;11(8):e0160764. Available from: https://doi.org/10.1371/journal.pone.0160764

13.	Geng EH, Glidden D V, Bwana MB, Musinguzi N, Emenyonu N, Muyindike W, et al. Retention in Care and Connection to Care among HIV-Infected Patients on Antiretroviral Therapy in Africa: Estimation via a Sampling-Based Approach. PLOS ONE [Internet]. 2011 Jul 26;6(7):e21797. Available from: https://doi.org/10.1371/journal.pone.0021797

14.	Ware NC, Wyatt MA, Geng EH, Kaaya SF, Agbaji OO, Muyindike WR, et al. Toward an Understanding of Disengagement from HIV Treatment and Care in Sub-Saharan Africa: A Qualitative Study. PLOS Medicine [Internet]. 2013 Jan 8;10(1):e1001369. Available from: https://doi.org/10.1371/journal.pmed.1001369

15.	Shubber Z, Mills EJ, Nachega JB, Vreeman R, Freitas M, Bock P, et al. Patient-Reported Barriers to Adherence to Antiretroviral Therapy: A Systematic Review and Meta-Analysis. PLOS Medicine [Internet]. 2016 Nov 29;13(11):e1002183. Available from: https://doi.org/10.1371/journal.pmed.1002183

16.	Geng EH, Odeny TA, Lyamuya R, Nakiwogga-Muwanga A, Diero L, Bwana M, et al. Retention in Care and Patient-Reported Reasons for Undocumented Transfer or Stopping Care Among HIV-Infected Patients on Antiretroviral Therapy in Eastern Africa: Application of a Sampling-Based Approach. Clinical Infectious Diseases [Internet]. 2016 Apr 1 [cited 2017 Dec 14];62(7):935–44. Available from: https://academic.oup.com/cid/article-lookup/doi/10.1093/cid/civ1004

17.	Larson E, Vail D, Mbaruku GM, Kimweri A, Freedman LP, Kruk ME. Moving Toward Patient-Centered Care in Africa: A Discrete Choice Experiment of Preferences for Delivery Care among 3,003 Tanzanian Women. PLOS ONE [Internet]. 2015 Aug 11;10(8):e0135621. Available from: https://doi.org/10.1371/journal.pone.0135621

18.	Peltzer K. Patient experiences and health system responsiveness in South Africa. BMC Health Services Research [Internet]. 2009 Jul;9(1):117. Available from: https://doi.org/10.1186/1472-6963-9-117

19.	Geng EH, Odeny TA, Lyamuya RE, Nakiwogga-Muwanga A, Diero L, Bwana M, et al. Articles Estimation of mortality among HIV-infected people on antiretroviral treatment in east Africa : a sampling based approach in an observational , multisite , cohort study. The Lancet HIV [Internet]. 2012 Mar 1 [cited 2018 Apr 12];2(3):10–2. Available from: https://www.sciencedirect.com/science/article/pii/S2352301815000028

20.	Mead N, Bower P. Patient-centredness: a conceptual framework and review of the empirical literature. Social Science & Medicine [Internet]. 2000;51(7):1087–110. Available from: http://www.sciencedirect.com/science/article/pii/S0277953600000988

21.	Scholl I, Zill JM, Härter M, Dirmaier J. An Integrative Model of Patient-Centeredness – A Systematic Review and Concept Analysis. PLOS ONE [Internet]. 2014 Sep 17;9(9):e107828. Available from: https://doi.org/10.1371/journal.pone.0107828

22.	Yehia BR, Mody A, Stewart L, Holtzman CW, Jacobs LM, Hines J, et al. Impact of the outpatient clinic experience on retention in care: Perspectives of HIV-infected patients and their providers. AIDS Patient Care and STDs [Internet]. 2015;29(7):365–9. Available from: http://search.ebscohost.com/login.aspx?direct=true&db=psyh&AN=2015-29378-001&site=ehost-live%5Cnhttp://byehia@upenn.edu

23.	Dang BN, Westbrook RA, Black WC, Rodriguez-Barradas MC, Giordano TP. Examining the Link between Patient Satisfaction and Adherence to HIV Care: A Structural Equation Model. PLoS ONE. 2013;8(1).

24.	Nayiga S, DiLiberto D, Taaka L, Nabirye C, Haaland A, Staedke SG, et al. Strengthening patient-centred communication in rural Ugandan health centres: A theory-driven evaluation within a cluster randomized trial. Evaluation [Internet]. 2014;20(4):471–91. Available from: http://journals.sagepub.com/doi/10.1177/1356389014551484

25.	Wachira J, Middlestadt S, Reece M, Peng CYJ, Braitstein P. Psychometric assessment of a physician-patient communication behaviors scale: The perspective of adult HIV patients in Kenya. AIDS Research and Treatment. 2013;2013.

26.	Grimsrud A, Bygrave H, Doherty M, Ehrenkranz P, Ellman T, Ferris R, et al. Reimagining HIV service delivery : the role of differentiated care from prevention to suppression. J Acquir Immune Defic Syndr. 2016;(19):10–2.

27.	Robone S, Rice N, Smith PC. Health systems’ responsiveness and Its characteristics: A cross-Country comparative analysis. Health Services Research. 2011;46(6 PART 2):2079–100.

28.	Murray CJL, Frenk J. A framework for assessing the performance of health systems. Bulletin of the World Health Organization [Internet]. 2000;78(6):717–31. Available from: http://www.scielosp.org/scielo.php?script=sci_arttext&pid=S0042-96862000000600004&lng=en&nrm=iso&tlng=e

29.	Geng EH, Nash D, Kambugu A, Zhang Y, Braitstein P, Christopoulos KA, et al. Retention in Care among HIV-Infected Patients in Resource-Limited Settings: Emerging Insights and New Directions. Current HIV/AIDS Reports [Internet]. 2010 Nov 7 [cited 2016 Aug 25];7(4):234–44. Available from: http://link.springer.com/10.1007/s11904-010-0061-5

30.	Camlin CS, Neilands TB, Odeny TA, Lyamuya R, Nakiwogga-Muwanga A, Diero L, et al. Patient-reported factors associated with reengagement among HIV-infected patients disengaged from care in East Africa. AIDS [Internet]. 2015;1. Available from: http://content.wkhealth.com/linkback/openurl?sid=WKPTLP:landingpage&an=00002030-900000000-97934

31.	Bassett I V, Coleman SM, Giddy J, Bogart LM, Chaisson CE, Ross D, et al. Barriers to care and 1-year mortality among newly-diagnosed HIV-infected people in Durban, South Africa. JAIDS Journal of Acquired Immune Deficiency Syndromes [Internet]. 2016;1. Available from: http://content.wkhealth.com/linkback/openurl?sid=WKPTLP:landingpage&an=00126334-900000000-97050

32.	Institute of Medicine & Committee on Quality of Health Care in America. Crossing the Quality Chasm: A New Health System for the 21st Century. Vol. 323, BMJ : British Medical Journal. 2001. 1192 p.

33.	Chimbindi N, Bärnighausen T, Newell M-L. Patient satisfaction with HIV and TB treatment in a public programme in rural KwaZulu-Natal: evidence from patient-exit interviews. BMC Health Services Research [Internet]. 2014;14(1):32. Available from: http://bmchealthservres.biomedcentral.com/articles/10.1186/1472-6963-14-32

34.	ICT Facts and Figures 2017 [Internet]. [cited 2017 Dec 15]. Available from: https://www.itu.int/en/ITU-D/Statistics/Pages/facts/default.aspx

35.	Mechael P, Batavia H, Kaonga N, Searle S, Kwan A, Goldberger A, et al. Barriers and Gaps Affecting mHealth in Low and Middle Income Countries: Policy White Paper. 2010 [cited 2017 Dec 15]; Available from: http://41.77.4.164:6510/www.globalproblems-globalsolutions-files.org/pdfs/mHealth_Barriers_White_Paper.pdf

36.	The Mobile Economy Sub-Saharan Africa 2017 [Internet]. [cited 2017 Dec 15]. Available from: https://www.gsmaintelligence.com/research/?file=7bf3592e6d750144e58d9dcfac6adfab&download

37.	ICT SURVEY REPORT - HOUSEHOLDS AND INDIVIDUALS [Internet]. [cited 2017 Dec 15]. Available from: https://www.zicta.zm/Views/Publications/2015ICTSURVEYREPORT.pdf

38.	Peter JE, Barron P, Pillay Y. Using mobile technology to improve maternal, child and youth health and treatment of HIV patients. South African Medical Journal [Internet]. 2015 Nov 16 [cited 2018 Jan 10];106(1):3. Available from: http://www.samj.org.za/index.php/samj/article/view/10209

39.	USAID Health Care Improvement Project (HCI) | Zambia | URC [Internet]. [cited 2017 Dec 15]. Available from: http://www.urc-chs.com/projects/usaid-health-care-improvement-project-hci-zambia

40.	mHealth: Zambia | World Vision International [Internet]. [cited 2017 Dec 15]. Available from: http://wvi.org/health/mhealth-zambia

41.	Agarwal S, Rosenblum L, Goldschmidt T, Carras M, Goal N, Labrique AB. Mobile Technology in Support of Frontline Health Workers. John Hopkins University Global mHealth Initiative 2016. 2016;86.

42.	McNabb M, Chukwu E, Ojo O, Shekhar N, Gill CJ, Salami H, et al. Assessment of the Quality of Antenatal Care Services Provided by Health Workers Using a Mobile Phone Decision Support Application in Northern Nigeria: A Pre/Post-Intervention Study. PLOS ONE [Internet]. 2015 May 5;10(5):e0123940. Available from: https://doi.org/10.1371/journal.pone.0123940

43.	Principles for Data Development. Principles | Principles for Digital Development [Internet]. 2018 [cited 2017 Dec 15]. Available from: https://digitalprinciples.org/principles/

44.	Agarwal S, LeFevre AE, Lee J, L’Engle K, Mehl G, Sinha C, et al. Guidelines for reporting of health interventions using mobile phones: mobile health (mHealth) evidence reporting and assessment (mERA) checklist. BMJ [Internet]. 2016 Mar 17;352. Available from: http://www.bmj.com/content/352/bmj.i1174.abstract

45.	Hickey MD, Omollo D, Salmen CR, Mattah B, Blat C, Ouma GB, et al. Movement between facilities for HIV care among a mobile population in Kenya: transfer, loss to follow-up, and reengagement. AIDS Care [Internet]. 2016 Nov 1;28(11):1386–93. Available from: https://doi.org/10.1080/09540121.2016.1179253

46.	Ministry of Health Z. Zambia Population-based HIV Impact Assessment (ZAMPHIA) 2016: First Report. 2017; Available from: http://phia.icap.columbia.edu/wp-content/uploads/2017/11/FINAL-ZAMPHIA-First-Report_11.30.17_CK.pdf

47.	I. Sikazwe, I. Eshun-Wilson, K. Sikombe, N. Czaicki, P. Somwe, A. Mody, S. Simbeza, D.V. Glidden, E. Chizema, L.B. Mulenga, N. Padian, C. Duncombe, C. Bolton-Moore, L.K.Beres, C.B. Holmes1 EG. Retention and viral suppression in a cohort of HIV patients on antiretroviral therapy: regionally representative estimates using a multi-stage sampling approach (In Press). To be published in PlosMed. 2019;

48.	Mwamba C, Sharma A, Mukamba N, Beres L, Geng E, Holmes CB, et al. ‘They care rudely!’: resourcing and relational health system factors that influence retention in care for people living with HIV in Zambia. BMJ Global Health [Internet]. 2018 Oct 25 [cited 2018 Nov 9];3(5):e001007. Available from: http://gh.bmj.com/lookup/doi/10.1136/bmjgh-2018-001007

49.	Zanolini A, Sikombe K, Sikazwe I, Eshun-Wilson I, Somwe P, Moore CB, et al. Understanding preferences for HIV care and treatment in Zambia: Evidence from a discrete choice experiment among patients who have been lost to follow-up. PLOS Medicine [Internet]. 2018 [cited 2018 Aug 21];15(8):e1002636. Available from: http://journals.plos.org/plosmedicine/article?id=10.1371/journal.pmed.1002636

50.	Copas AJ, Lewis JJ, Thompson JA, Davey C, Baio G, Hargreaves JR. Designing a stepped wedge trial: Three main designs, carry-over effects and randomisation approaches. Trials. 2015;

51.	English M, Irimu G, Wamae A, Were F, Wasunna A. Europe PMC Funders Group Health systems research in a low income country - easier said than done. 2009;93(6):540–4.

52.	Ortblad KF, Musoke DK, Chanda MM, Ngabirano T, Velloza J, Haberer JE, et al. Knowledge of HIV Status Is Associated with a Decrease in the Severity of Depressive Symptoms among Female Sex Workers in Uganda and Zambia. Journal of Acquired Immune Deficiency Syndromes. 2020;

53.	Beach MC, Saha S, Korthuis PT, Sharp V, Cohn J, Wilson I, et al. Differences in patient-provider communication for hispanic compared to non-hispanic white patients in HIV care. Journal of General Internal Medicine. 2010;25(7):682–7.

54.	Beach MC, Saha S, Korthuis PT, Sharp V, Cohn J, Wilson IB, et al. Patient-provider communication differs for black compared to white HIV-infected patients. AIDS and Behavior. 2011;15(4):805–11.

55.	Labhardt ND, Aboa SM, Manga E, Bensing JM, Langewitz W. Bridging the gap: How traditional healers interact with their patients. A comparative study in Cameroon. Tropical Medicine and International Health. 2010;15(9):1099–108.

56.	Labhardt ND, Schiess K, Manga E, Langewitz W. Provider-patient interaction in rural Cameroon-How it relates to the patient’s understanding of diagnosis and prescribed drugs, the patient’s concept of illness, and access to therapy. Patient Education and Counseling [Internet]. 2009 Aug 1 [cited 2018 Jun 14];76(2):196–201. Available from: http://www.ncbi.nlm.nih.gov/pubmed/19168317

57.	Jenson A, Gracewello C, Mkocha H, Roter D, Munoz B, West S. Gender and performance of community treatment assistants in Tanzania. International Journal for Quality in Health Care. 2014;26(5):524–9.

58.	Kim YM, Kols A, Mwarogo P, Awasum D. Differences in counseling men and women: Family planning in Kenya. Patient Education and Counseling. 2000;39(1):37–47.

59.	MacLachlan EW, Shepard-Perry MG, Ingo P, Uusiku J, Mushimba R, Simwanza R, et al. Evaluating the effectiveness of patient education and empowerment to improve patient-provider interactions in antiretroviral therapy clinics in Namibia. AIDS Care - Psychological and Socio-Medical Aspects of AIDS/HIV [Internet]. 2016 May 3 [cited 2017 Dec 14];28(5):620–7. Available from: http://www.tandfonline.com/doi/full/10.1080/09540121.2015.1124975

60.	Kilian S, Swartz L, Chiliza B. Doing their best: strategies used by South African clinicians in working with psychiatric inpatients across a language barrier. Global Health Action [Internet]. 2015 Dec 1;8(1):28155. Available from: https://doi.org/10.3402/gha.v8.28155

61.	Roter D, Larson S. The Roter interaction analysis system (RIAS): utility and flexibility for analysis of medical interactions. Patient Education and Counseling [Internet]. 2002;46(4):243–51. Available from: http://www.sciencedirect.com/science/article/pii/S0738399102000125

62.	Frangakis CE, Rubin DB. Addressing an Idiosyncrasy in Estimating Survival Curves Using Double Sampling in the Presence of Self-Selected Right Censoring. Biometrics [Internet]. 2001 Jun 1 [cited 2017 Nov 8];57(2):333–42. Available from: http://doi.wiley.com/10.1111/j.0006-341X.2001.00333.x

63.	Guidelines for Mystery Shopping [Internet]. 2011 [cited 2018 Jan 24]. Available from: www.mspa-global.org

64.	Martínez-Pérez B, de la Torre-Díez I, López-Coronado M. Privacy and Security in Mobile Health Apps: A Review and Recommendations [Internet]. Vol. 39, Journal of Medical Systems. 2014. p. 181. Available from: http://link.springer.com/10.1007/s10916-014-0181-3

65.	Rhodes K V., Miller FG. Simulated patient studies: An ethical analysis. Milbank Quarterly. 2012;90(4):706–24.

66.	INSTITUTIONAL REVIEW BOARD FOR THE PROTECTION OF HUMAN SUBJECTS IN RESEARCH [Internet]. [cited 2018 Jan 24]. Available from: https://www.unh.edu/research/sites/www.unh.edu.research/files/docs/RIS/anonymity_confidentialityprivacy.pdf
